# A Phase II study of neoadjuvant axitinib for reducing the extent of venous tumour thrombus in clear cell renal cell cancer with venous invasion (NAXIVA)

**DOI:** 10.1038/s41416-022-01883-7

**Published:** 2022-06-23

**Authors:** Grant D. Stewart, Sarah J. Welsh, Stephan Ursprung, Ferdia A. Gallagher, James O. Jones, Jacqui Shields, Christopher G. Smith, Thomas J. Mitchell, Anne Y. Warren, Axel Bex, Ekaterini Boleti, Jade Carruthers, Tim Eisen, Kate Fife, Abdel Hamid, Alexander Laird, Steve Leung, Jahangeer Malik, Iosif A. Mendichovszky, Faiz Mumtaz, Grenville Oades, Andrew N. Priest, Antony C. P. Riddick, Balaji Venugopal, Michelle Welsh, Kathleen Riddle, Lisa E. M. Hopcroft, NAXIVA Trial Group, Niki Couper, Lisa E. M. Hopcroft, Robert Hill, Athena Matakidou, Cara Caasi, James Watson, Lauren Wallis, Ruby Cross, Sarah W. Burge, Anne George, Tobias Klatte, Tevita F. Aho, James N. Armitage, Sabrina Rossi, Charlie Massie, Shubha Anand, Tiffany Haddow, Marc Dodd, Wenhan Deng, Ezequiel Martin, Philip Howden, Stephanie Wenlock, Evis Sala, Stefan Symeonides, Lynn Ho, Jennifer Baxter, Stuart Leslie, Duncan McLaren, John Brush, Marie O’Donnell, Alisa Griffin, Ruth Orr, Catriona Cowan, Thomas Powles, Anna Pejnovic, Sophia Tincey, Lee Grant, Martin Nuttall, Lucy Willsher, Christian Barnett, David Nicol, James Larkin, Alison Fielding, Robert J. Jones

**Affiliations:** 1 University of Cambridge, Cambridge, UK; 2 Cambridge University Hospitals NHS Foundation Trust, Cambridge, UK; 3 MRC Cancer Unit, University of Cambridge, Cambridge, UK; 4 CRUK Cambridge Institute, Cambridge, UK; 5 Wellcome Sanger Institute, Cambridge, UK; 6 Royal Free London NHS Foundation Trust, London, UK; 7 Scottish Clinical Trials Research Unit, Public Health Scotland, Edinburgh, UK; 8 Broomfield Hospital, Chelmsford, UK; 9 Western General Hospital, Edinburgh, UK; 10 Institute of Genetics and Cancer, University of Edinburgh, Edinburgh, UK; 11 NHS Greater Glasgow and Clyde, Glasgow, UK; 12 University of Glasgow, Glasgow, UK; 13 Royal Marsden Hospital, London, UK; 14 Present Address: School of Cancer and Pharmaceutical Sciences, King’s College London, London, UK; 15 Present Address: Nuffield Department of Primary Care Health Sciences, University of Oxford, Oxford, UK

**Keywords:** Surgical oncology, Renal cell carcinoma, Predictive markers

## Abstract

**Background:**

Surgery for renal cell carcinoma (RCC) with venous tumour thrombus (VTT) extension into the renal vein (RV) and/or inferior vena cava (IVC) has high peri-surgical morbidity/mortality. NAXIVA assessed the response of VTT to axitinib, a potent tyrosine kinase inhibitor.

**Methods:**

NAXIVA was a single-arm, multi-centre, Phase 2 study. In total, 20 patients with resectable clear cell RCC and VTT received upto 8 weeks of pre-surgical axitinib. The primary endpoint was percentage of evaluable patients with VTT improvement by Mayo level on MRI. Secondary endpoints were percentage change in surgical approach and VTT length, response rate (RECISTv1.1) and surgical morbidity.

**Results:**

In all, 35% (7/20) patients with VTT had a reduction in Mayo level with axitinib: 37.5% (6/16) with IVC VTT and 25% (1/4) with RV-only VTT. No patients had an increase in Mayo level. In total, 75% (15/20) of patients had a reduction in VTT length. Overall, 41.2% (7/17) of patients who underwent surgery had less invasive surgery than originally planned. Non-responders exhibited lower baseline microvessel density (CD31), higher Ki67 and exhausted or regulatory T-cell phenotype.

**Conclusions:**

NAXIVA provides the first Level II evidence that axitinib downstages VTT in a significant proportion of patients leading to reduction in the extent of surgery.

**Clinical trial registration:**

NCT03494816.

## Introduction

Venous tumour thrombus (VTT) extension into the renal vein (RV) and/or inferior vena cava (IVC) occurs in 4–15% cases of renal cell cancer (RCC) [1]. Peri-surgical mortality is high (5–15%) and increases with the height of the VTT [1, 2]. Following this extensive surgery, the cure is possible with 5-year survival rates of ~40–65% for patients with non-metastatic RCC [3, 4]. The concept of using targeted therapies, to downstage VTT prior to surgery is appealing. It is hypothesised that by reducing the level of the VTT and the extent of surgery, morbidity and mortality would be reduced.

There is no Level I or II evidence of pre-surgical targeted therapy in non-metastatic or metastatic RCC VTT. Four retrospective studies focused on mixed groups of vascular endothelial growth factor receptor (VEGFR) tyrosine kinase inhibitors (TKI) [5–8]: sunitinib [9, 10], axitinib [11] and pazopanib [12]. VTT level decreased in a median of 22.6% patients (range 14.9–32.9%), remained stable in 73.6% (64.1–81.4%) and increased in 7.2% (3.4–14.3%). Results were most favourable for sunitinib and axitinib [5, 7, 11]. There are several prospective studies on VEGFR TKIs in the pre-nephrectomy setting [13–15], but none specifically addresses the question of surgical downstaging of vein-involved local extension. Wood et al. reported on four patients with IVC VTT but reported no change in surgical management, and did not report specifically about change in the extent of venous involvement [13]. In a Phase II trial of 12 weeks of neoadjuvant axitinib in clear cell RCC (ccRCC; all patients were cT3a), the median reduction in primary tumour diameter was 28% [15]. Most of the reduction in tumour size had occurred within 7 weeks of axitinib treatment. The results of these small studies in non-metastatic RCC patients suggest that neoadjuvant VEGFR TKI treatment of RCC patients is safe and reduces tumour size. However, the effect of these drugs on the extent of the VTT and the effect on the surgical approach has not been confirmed.

The objective of NAXIVA was to determine safety, efficacy and effect of neoadjuvant axitinib on VTT.

## Patients and methods

### Study design

NAXIVA was a single-arm, single agent, open-label, multi-site, UK-based, Phase II feasibility study of 8 weeks axitinib treatment in M0 and M1 patients with resectable ccRCC primary tumours with VTT. NAXIVA was prospectively, publicly registered (ISCRTN96273644; EudraCT Number 2017-000619-17; NCT03494816) and approved by an independent ethics committee (REC reference: 17/EE/0240). See Appendix for the full study protocol.

### Endpoints

**Fig. 1:**
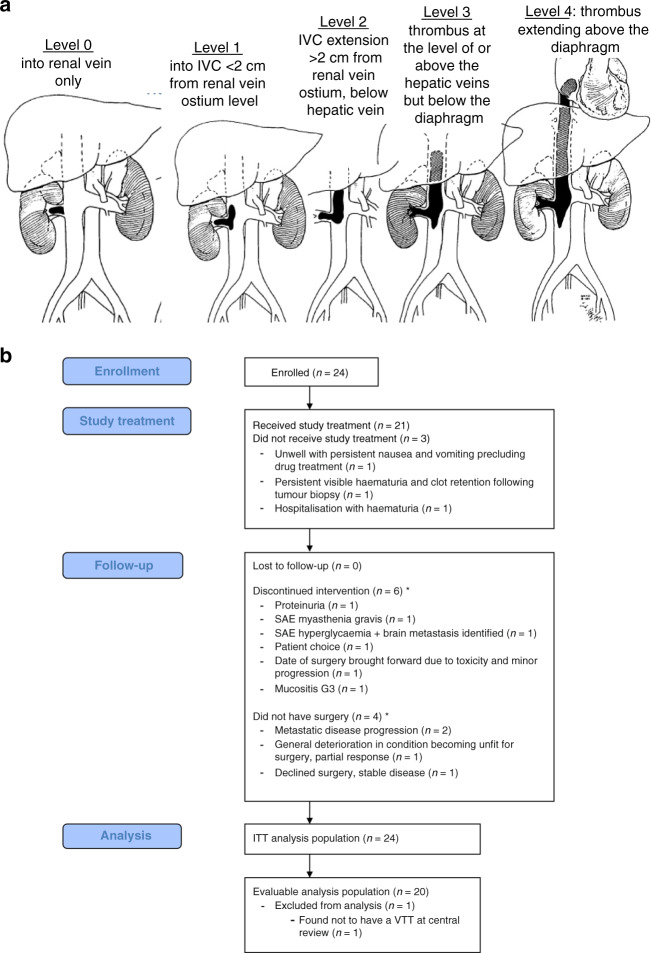
Description of Mayo level and study cohort. **a** Summary of Mayo level, figure adapted from ref. [37]. **b** Consort diagram. *Participants who had at least one dose of the study drug were included in the evaluable population, irrespective of whether surgery was performed.

The primary endpoint was the percentage of evaluable patients with a reduction in the extent of VTT by Mayo level after 8 weeks of axitinib therapy. Definitions of the Mayo level (levels are ordered by increasing extensiveness; Fig. 1a) as previously described [2]:

- Level 0: thrombus limited to the renal vein (RV);
- Level 1: into IVC < 2 cm from RV ostium level;
- Level 2: IVC extension >2 cm from RV ostium but below hepatic veins;
- Level 3: thrombus at the level of or above the hepatic veins but below the diaphragm;
- Level 4: thrombus extending above the diaphragm.

Secondary endpoints were percentage change in surgical approach, the percentage change in VTT length, response rate by RECIST version 1.1, and evaluation of surgical morbidity assessed by Clavien–Dindo classification [16]. Exploratory endpoints were translational studies correlating changes in molecular markers with the response to axitinib in the VTT and primary tumour.

### Participants/eligibility criteria

Key inclusion criteria were RV (cT3a) or IVC VTT (cT3b/c), N0/1, M0/1, biopsy-proven ccRCC, over 18 years of age, suitable for immediate surgical resection of the primary tumour. Participants had to be Eastern Cooperative Oncology Group (ECOG) performance status <2; have urinalysis <2+ protein, urinary protein <2 g/24 h or protein:creatinine ratio (PCR) < 200 mg/mmol; and serum creatinine ≤1.5 × ULN or estimated creatinine clearance ≥30 mL/min calculated using the Cockcroft–Gault equation. Key exclusion criteria were Memorial Sloan Kettering Cancer Center (MSKCC) poor-risk disease (M1 participants) and recent history of cardiac or vascular events.

### Drug treatment

The starting dose of axitinib was 5 mg BD, escalated to 7 mg BD and then 10 mg BD every 2 weeks, as tolerated. Dose reductions were allowed. Patients stopped axitinib a minimum of 36 h and a maximum of 7 days prior to surgery in week 9.

### Assessments

Patients had clinical and safety assessments according to the Schedule of Assessments (see Protocol in the Appendix). Axitinib-related toxicity was assessed using Common Terminology Criteria for Adverse Events version 4 (CTCAEv4) criteria. MRI scans were performed before treatment, during week 3 and before surgery (see Supplementary Methods for MRI protocol). CT scans were performed before treatment, week 3 (M0 patients only to assess for development of chest metastases), week 9- and 3-months post surgery.

### Surgery

Surgeons were asked to report their planned approach to the VTT after reviewing the baseline MRI scan and record the performed approach after axitinib therapy, plus planned and performed adjuvant venous procedures. RV/IVC level of control planned/performed intraoperatively was recorded:

- Thrombus milked into RV and side clamped;
- Infrahepatic IVC clamping with no liver mobilisation;
- Retrohepatic IVC clamping below hepatic veins, with liver mobilisation;
- Retrohepatic IVC clamping above hepatic veins, with liver mobilisation;
- Suprahepatic, infradiaphragmatic clamping;
- Suprahepatic, supradiaphragmatic clamping.

### Outcome measures

Mayo level and VTT length were assessed using the baseline and week-9 MRI scans, if no week-9 scan was undertaken, the week 3 scan (if available) was used; calculation details are provided in Supplementary Methods. In order to minimise reporter bias due to the inability to the blind, the primary and relevant secondary endpoint data was based on a consensus by two central uroradiologists’ (SU and FAG) review of the MRI images.

The response rate was determined at local sites using RECIST version 1.1 comparing the screening (baseline) and pre-surgical CT scans. Primary tumour measurements were included in RECISTv1.1 measurements in all patients. Surgical morbidity was assessed by Clavien–Dindo classification [16].

### Method of calculating primary endpoints

The definition of an improvement varied according to the patient’s Mayo level as captured at screening. For patients presenting at screening with a Mayo level 1 or above, an improvement in disease was represented by a reduction in their Mayo level at week 9. For patients presenting at the screening with Mayo-level 0, an improvement in disease was represented by either: a change of VTT from the main renal vein to branches of the renal vein (on the right); or a change of VTT from main renal vein to the renal vein lateral to the gonadal vein (on the left), or if the VTT was lateral to the gonadal vein at screening, a change from the main renal vein lateral to the gonadal vein to the branches of the renal vein. This response designation for RV-only patients was developed as such changes would enable minimally invasive surgery to be undertaken. The number and percentage of patients with no change in VTT status or extension of the VTT into the inferior vena cava between screening and week 9 was recorded.

### Method of calculating secondary endpoint of percentage change in VTT length

Percentage change in VTT length was calculated using the following methodology for each timepoint as follows:

1. Calculate the sum of (i) length of RV thrombus; (ii) the length of IVC tumour thrombus ABOVE RV (measured from midpoint of the ostium of RV + IVC to tip of tumour thrombus); (iii) the length of IVC tumour thrombus BELOW RV (measured from midpoint of the ostium of RV + IVC to the tip of tumour thrombus) at timepoint T. Note that in RV-only patients only distance (i) is measurable;
2. Calculate the percentage reduction at each timepoint T as follows: 1-(*Sum*_*T*_/*Sum*_0_), where *Sum*_*T*_ is the sum calculated as in Step 1 for timepoint T, and *Sum*_0_ is the sum calculated as in Step 1 at baseline.

### Method of calculating secondary endpoint of percentage change in surgical approach

Percentage change in surgical approach was determined by comparing the surgeon-reported planned vs performed surgical approaches using three pieces of data:

1. Change from “Open Surgery” to “Minimally invasive surgery”;
2. Change from a more invasive open to a less invasive open surgical approach (between that planned by surgeons based on the baseline MRI scan and that actually performed);
3. Less extensive surgical incision used.

### Statistical plan

A Simon two-stage minimax design [17] was used to distinguish a ≤5% from a ≥25% cohort improvement in the Mayo level this required 20 evaluable patients (90% power, 10% one-sided). For the clinical trial to be considered a success, at least three evaluable patients would demonstrate an improvement in disease on treatment between screening and week 9.

In the two-stage design, 13 patients were to be recruited in the first stage. If no patients demonstrated an improvement in their Mayo level between screening and week 9, accrual to the clinical trial would stop. If one or more patients demonstrated an improvement in the Mayo level between screening and week 9, the final seven patients would be recruited.

The intention-to-treat (ITT) population included all patients registered in the study. The evaluable and safety populations included all patients in the ITT population who received at least one dose of the study drug (including any patients who were enrolled in error, received the study drug and/or were subsequently found to be ineligible).

In all, 80% two-sided confidence intervals (to correspond to the 10% one-sided sample size calculation) for the proportions relevant to the efficacy endpoints were calculated using the approach of Koyama and Chen [18].

All analyses were carried out using R v3.5.1 and reporting was heavily supported by the CTutils package (https://github.com/LisaHopcroft/CTutils). The trial data upload to EudraCT was enabled, in part, by the EudraCT package (https://eudract-tool.medschl.cam.ac.uk/).

### Biosampling

Blood, urine and tissue (fresh frozen and formalin-fixed paraffin-embedded (FFPE)) samples for translational studies were taken prior to, during and after therapy to evaluate biomarkers of treatment response according to the Schedule of Assessments in the Protocol; see Appendix). Samples were processed and stored according to the NAXIVA Laboratory Manual (see Appendix).

### Immunofluorescence

Formalin-fixed paraffin-embedded sections were dewaxed in xylene and rehydrated in graded alcohols prior to antigen retrieval in Tris-EDTA pH9. Slides were blocked and incubated with primary antibodies at 4 °C overnight (CD31 (JC/70A, Abcam), Ki67 (EPR3610, Abcam), CD8 (SP16, Invitrogen), Granzyme B (NCL-L-GRAN-B, Leica), PD-1 (AF1086, RnD Systems), CD4 (EPR6855, Abcam), FOXP3 (236A/E7, Abcam), SMA (ab5694, Abcam), CD68 (KP1, Invitrogen)). Samples were washed and incubated in fluorescently conjugated secondary antibodies; nuclei were counterstained with DAPI. Whole slides were scanned at ×40 magnification on the Zeiss Axio Scan Z1 system. Image analysis was performed using HALO Software (Indica Labs, analysis algorithms: HighPlex FL v3.1.0, Object Colocalization FL v1.0, Area Quantification FL v2.1.5).

### ctDNA analysis

ctDNA analysis was carried out as published previously [19]. Briefly, cell-free DNA was extracted from blood and urine using the QIASymphony platform (QIAGEN). Libraries were prepared from DNA using the Thruplex Tag-Seq protocol (Takara) and sequenced on the Illumina HIseq4000 platform. Sequence data were analysed using an ‘in-house’ pipeline that consists of the following: paired-end sequence reads were aligned to the human reference genome (GRCh37) after removing any contaminant adapter sequences. Duplicate reads or reads of with low mapping quality/secondary alignments were excluded from downstream analysis. Data were analysed with the ichorCNA algorithm, version 0.2.0, using default parameters [20]. Samples were deemed to have ‘detected ctDNA’ if the predicted tumour fraction score was >0.025, and visual inspection of copy number plots confirmed somatic copy number aberrations.

## Results

### Patient characteristics

Figure 1b and Table 1 detail patients recruited between December 2017 and January 2020. In total, 21 participants at five centres made up the evaluable population. On central review of imaging, one of the 21 patients was found not to have a VTT, making 20 patients who were both eligible and evaluable and in whom the study endpoints are reported.

**Table 1:** Baseline characteristics of the evaluable population.

Characteristic	*n*	%
Number of patients	21	100
Median age, year (range)	69 (49–78)	
Sex		
Male	15	71
Female	6	29
Median BMI, kg/m^2^ (range)	27.7 (19.4–44.6)	
ECOG grade		
0	13	61.9
1	8	38.1
Clinical T-stage		
T3a	6	28.6
T3b	13	61.9
T3c	2	9.5
M-stage		
M0	11	52.4
M1	10	47.6
Median number of metastases (range)	1 (1–4)	
Site of metastases		
Lymph nodes	2	18.2
Adrenal	1	9.1
Lung	7	63.6
Bone	1	9.1
MSKCC classification (M1 patients only)		
Intermediate	9	90
Poor^*^	1	10
Histological subtype on baseline biopsy		
ccRCC	21	100
ISUP grade on baseline biopsy		
1	2	9.5
2	10	47.6
3	2	9.5
4	4	19.0
No data	3	14.3
Mayo level of VTT on baseline imaging^+^		
RV only (level 0)	4	20
Level 1	3	15
Level 2	9	45
Level 3	2	10
Level 4	2	10

^*^For eligibility M1 participants had to be an intermediate risk by MSKCC criteria. This patient was entered into the trial when they were thought to have M0 disease. Central imaging review following completion of the trial identified M1 disease at baseline and retrospectively the patient was found to have MSKCC poor-risk disease (newly diagnosed RCC, haemoglobin, LDH). However, as they received study drug and had a VTT they were in the evaluable population and remain in the study analysis.

^+^One evaluable patient was found on central imaging review to be ineligible for NAXIVA as they did not have a VTT; thus the baseline VTT level is only available for the 20 eligible and evaluable patients.

### Primary endpoint-reduction in Mayo level

**Fig. 2:**
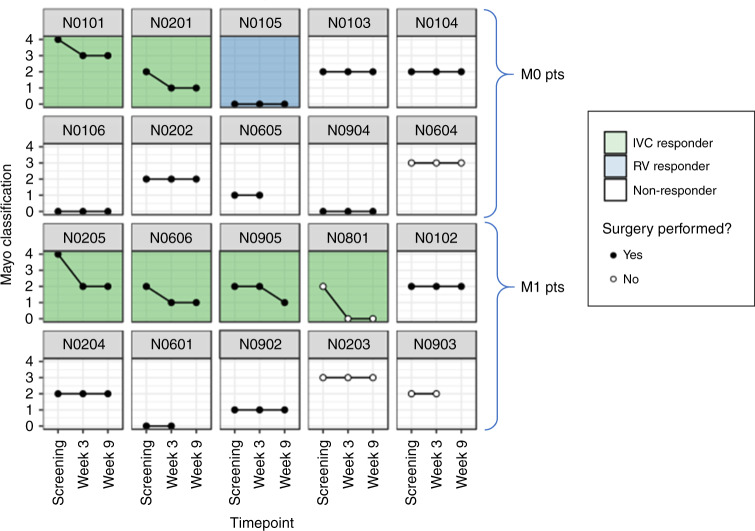
Mayo level at baseline, week 3 and week 9 for eligible and evaluable patients. Note that N105 had a RV-only VTT response receding from the medial to the insertion of the gonadal vein to lateral to it. Supplementary Fig. S1 shows examples of two IVC responder patients.

Of the 20 eligible and evaluable patients, 37.5% (6/16) IVC VTT patients had a reduction in Mayo level and 25% (1/4) patients with RV-only VTT responded (Fig. 2). Hence, the overall response rate in evaluable and eligible patients with VTT was 35.0% (7/20). The remaining 13 patients had a stable Mayo level (65%), and none had an increase in Mayo level. By the inference procedures for Simon's two-stage minimax design there was a response rate of 32.8% [80% CI 20.7%, 46.7%]. This was a statistically significant result (*P* = 3.395 × 10^−5^), where the null hypothesis that the true response rate is <5% can be rejected in favour of the alternative hypothesis of a 'good' (>25%) response.

### Secondary endpoint-percentage change in venous tumour thrombus length

**Fig. 3:**
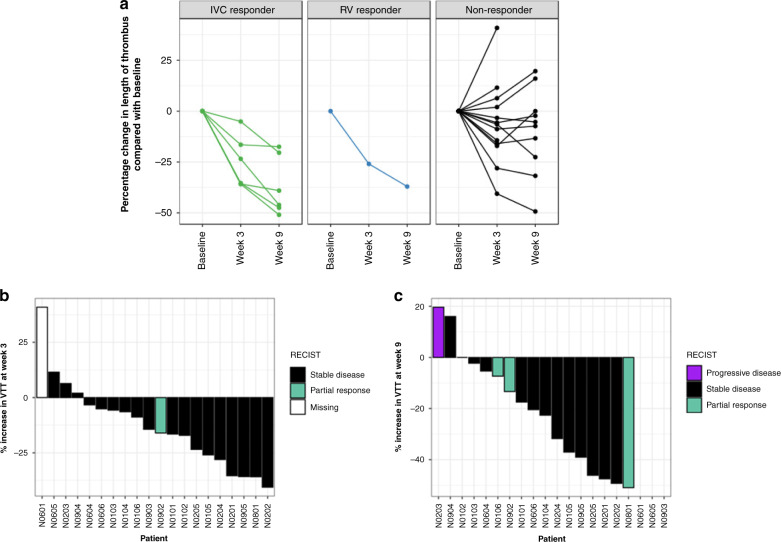
Percentage change in VTT length over the axitinib treatment period. **a** Line chart showing percentage change in VTT length for IVC responders, RV responders and non-responders. Waterfall plot of VTT response against tumour response at (**b**) 3 and (**c**) 9 weeks of treatment. N0601 (surgery expedited), N0605 (surgery expedited) and N0903 (exited trial due to new brain metastasis) did not have scans at week 9. Bar colour indicates the patient’s overall RECIST status distinct from VTT assessment.

Although 65% (13/20) patients had a stable Mayo level (Fig. 2; classed as ‘non-responders’), seven of these 13 patients had a percentage reduction in the VTT length after 8 weeks of axitinib, therefore 15 of 20 patients (75%) had any degree of reduction in VTT length (range 2–51%) (Fig. 3). One patient (5%) had no change in VTT length. At week 3, four patients (20%) had an increase in VTT length, two had surgery expedited as detailed below. For all patients, the direction of change in VTT on the week 3 safety MRI was predictive of the response at 9 weeks (Figs. 2 and 3).

There was a 15.2% (range −41% to 41%; negative numbers indicating an increase in length) and 27.2% (range −20% to 51%) median reduction in VTT length at weeks 3 and 9, respectively.

### Absolute changes in VTT length

The percentage change in VTT length, equated to an absolute median reduction in VTT length at weeks 3 and 9 of 10 mm (range −12 to 56 mm) and 20 mm (range −34 to 68 mm), respectively. In four patients who had an increase in length of VTT at 3 weeks, increases were 1 mm, 9 mm, 11 mm and 12 mm and at 9 weeks for the two patients with an increase in VTT these were 8 mm and 34 mm. IVC VTT was identified and measured both above and below the ostium with the RV in 14 of 16 patients with IVC VTT (Supplementary Fig. S2). Changes in IVC VTT length on axitinib below the RV ostium trended with the changes of VTT above the RV ostium.

### Secondary endpoint-RECIST response

At week 3, one patient (5% of those having scan) had a RECIST-defined partial response (PR), 19 patients (95%) had stable disease (SD) and data were missing for one patient (N0601) who failed to attend the MRI (Supplementary Table S1). By week 9, 3 patients (16.7%) had a PR, 13 (72.2%) had SD, 2 (11.1%) had PD, and data were missing for three patients as they had exited the trial. None of the M0 patients became M1 during the trial.

At week 9, 7 of 17 patients (41.2%) had a PR in their VTT (i.e. >30% reduction in length) (Fig. 3b, c).

### Secondary endpoint-surgical approach

In total, 17 patients underwent surgery. Despite an inclusion criterion for NAXIVA being suitability for surgery, four patients did not have surgery (19.0%; three M1 and one M0). Of the M1 patients, reasons for not having surgery were the progression of metastatic disease despite axitinib (*n* = 2) and partial response but a general performance status decline resulting in becoming unfit for surgery (*n* = 1). One M0 patient had stable disease at week 9 but declined surgery. Surgery was brought forward in two patients from the planned surgery date of week 9. One patient stopped drug after 16 days and another after 33 days.

Improvement in the ‘level of control’ of IVC/renal vein was observed in five out of 17 (29.4%) patients (Supplementary Table S2). No patients had deterioration in ‘level of control’ of IVC/renal vein performed relative to that planned. Two patients had a change of approach from planned open to performed minimally invasive surgery (one also had an improved, lower venous ‘level of control’). One additional patient had a substantially smaller incision (planned thoracoabdominal & midline laparotomy to perform subcostal and midline laparotomy). Therefore, 7/17 (41.1%) patients had a less extensive surgery performed than was planned prior to axitinib treatment. Four Mayo ‘responders’ also had a reduction in extent of surgery. In 16 patients, the VTT tissue was macroscopically cleared.

### Planned and performed surgery

Supplementary Tables S2 and S3 detail the planned and performed surgery in terms of correlation between Mayo change and change in surgery. Four Mayo ‘responders’ also had a reduction in the extent of surgery (N0205, N0101, N0105 and N0201). Two Mayo responders did not have change in surgery (N0905 and N0606); these were both reduction from level 2 to level 1 and for both the surgeon predicted and performed ‘Infrahepatic (IVC clamping with no liver mobilisation)’. Cardiac surgery and performing a Pringle manoeuvre are both morbid and in NAXIVA two patients (N0101 and N0205) had supradiaphragmatic surgery and/or hypothermic cardiac arrest predicted and both had a reduction to infradiaphragmatic surgery performed (N0205 to Retrohepatic (liver mobilisation and clamping below hepatic veins; N0101 to Suprahepatic (infradiaphragmatic)). There were no suprahepatic/infradiaphragmatic cases predicted at baseline. One patient was planned to have a venovenous bypass and one to have hypothermic cardiac arrest but following treatment, neither of these manoeuvrers was needed. In terms of patients with infrahepatic (IVC clamping with no liver mobilisation) planned at baseline, two (N0201 and N0904) actually had thrombus milked back into the renal vein and side clamping performed. A further three patients had improvement in surgery but no change in Mayo level (N0103, N0904 and N0901). One patient with a Mayo response did not have surgery as described above (N0801).

### Intra- and post-operative details and complications

Median operation time was 240 min (range 120–720 min). Median estimated blood loss was 1000 ml (range 50–7000 ml). Six patients had an intraoperative complication, five related to bleeding with two patients requiring a transfusion and one patient had an intraoperative cerebrovascular accident (CVA; identified post-operatively). Six patients had a post-operative complication of any grade (35.3%). Four complications were Clavien–Dindo 1/2 (expected CPAP post operation, persistent wound pain, one chest and one wound infection) and two were Grade 3 or above (11.8%). Poor wound healing is a concern during VEGFR TKI use, but all patients had discontinued axitinib prior to surgery and no issues of wound healing were reported. One patient had a cardiorespiratory arrest requiring one round of CPR to resuscitate (IVa), another had a CVA intraoperatively and died (V) (1/17 = 5.9% mortality rate). None of these events was considered to have been caused by axitinib. Seven patients had planned or unplanned ITU admissions post-operatively (41.2%). No patients had a delayed surgical complication at 6 or 12-weeks post-surgery follow-up.

### Axitinib dose delivered and duration of therapy

Supplementary Fig. S3a illustrates the axitinib dose received per patient. Axitinib dose was escalated in 12 of 21 patients (57%), two patients (9.5%) required dose reduction from the 5 mg b.d. starting dose. The median daily dose received (excluding breaks) was 5.8 mg b.d. (range 3.1–8.0 mg b.d.). The total dose of axitinib was not significantly different between patients with or without a Mayo-level response (*P* = 0.405). However, patients who did not have an improvement in Mayo level or a RECIST response received a significantly lower total dose of axitinib (*P* = 0.030) (Supplementary Fig. S3b) and had a shorter duration of axitinib treatment (excluding breaks) compared to patients who had a Mayo-level improvement (*P* = 0.026) (Supplementary Fig. S3c) or had either a Mayo or a RECIST response (*P* = 0.007) (Supplementary Fig. S3d). There was no correlation between the total dose of axitinib and VTT reduction at week 9 (Pearson’s *r*(16) = 0.07, *P* = 0.78).

### Adverse events (AEs)

Serious AEs whilst on axitinib were myasthenia gravis (recovered following nephrectomy, not after stopping axitinib), pathological fracture, hyperglycaemia, left cerebellar mass development, wound pain, confusion and hyperkalaemia. None were judged by local investigators to be related to axitinib. Table 2 and Supplementary Fig. S4 detail AEs related to axitinib by CTCAEv4 grade. AEs were consistent with previous data and did not delay surgery. No grade 4 or 5 AEs were observed. Correlations with clinical features are detailed in supplementary results.

**Table 2:** Drug toxicity by CTCAEv4 grade.

Event, %	Any grade	Grade 3*
Treatment-related adverse events in ≥10% of patients	100	52
Hypertension	86	24
Fatigue	67	10
Proteinuria	48	5
Voice alteration	48	0
Mucositis	43	10
Diarrhoea	38	0
Constipation	33	0
Back pain	29	0
Cough	29	0
Weight loss	29	0
Insomnia	24	0
Muscular weakness	24	5
Abdominal pain	19	0
Dry skin	19	0
Dysgeusia	14	0
Epistaxis	14	0
Headache	14	0
Hypothyroidism	14	0
PPE syndrome	14	0
Stomatitis	14	0
Vomiting	14	0

*No grade 4 or 5 AEs were observed.

Patients with either a Mayo-level response (*P* = 0.0034) and/or those with a RECIST response (*P* = 0.0003) had significantly lower maximum levels of proteinuria during treatment than non-responders (range 0–1 in responders vs 0–3 in non-responders). Baseline proteinuria was not significantly different between responders and non-responders (*P* = >0.05). Neither mean baseline systolic or diastolic blood pressure (BP), change in systolic or diastolic BP during treatment, nor maximum systolic or diastolic BP reached during treatment correlated with Mayo response (*P* = >0.05).

### Translational analyses

**Fig. 4:**
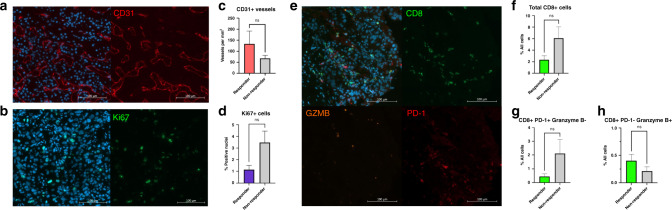
Immumofluorescence analysis of baseline biopsies. Representative images of baseline biopsies stained for **a** blood vessels (CD31), **b** proliferating cells (Ki67) and **e** CD8 + T-cell activation status (Granzyme B and PD-1). Whole slides were scanned and quantified using automated computer image analysis on HALO (**c**, **d**, **f**–**h** two-tailed Student *t* test).

Baseline biopsies, available from 17 patients, were assessed for the presence of markers associated with treatment outcome in ccRCC [21–23]. There was a trend for higher CD31 microvessel density in responders (Fig. 4a, c) and higher Ki67 index in non-responders (Fig. 4b, d).

Non-responders exhibited trends toward higher T-cell infiltration but populations shifted towards exhausted (PD-1 + ) or regulatory (FOXP3 + ) phenotypes compared to an activated (PD-1- granzyme B + ) phenotype in responders (CD8 + cells: Fig. 4e–h; CD4 + cells: Supplementary Fig. S5a–c). No differences were observed in other stromal markers (Supplementary Fig. S5d, e).

Consistent with previous studies showing low detection of ctDNA in RCC, only 25% (5/20) of patients (two in plasma, three in urine) had detectable ctDNA at baseline. There was no concordance in the levels or composition of ctDNA between the plasma and urine. Only 20% (1/5) of patients with detectable ctDNA at baseline showed an improvement in Mayo level or RECIST response.

## Discussion

NAXIVA is the first prospective study to evaluate drug treatment in managing RCC VTT, a frequently discussed question in clinical practice. The trial met its primary and secondary endpoints demonstrating that it is feasible to use systemic therapy to downstage VTT of all Mayo levels and reduce the extent of surgery in patients with resectable M0 and M1 ccRCC. Importantly, axitinib and surgical toxicity, morbidity and mortality were as expected [2] and no patient had clinically relevant VTT progression.

Ordinarily, surgery for patients with VTT would be expedited because of concern about disease progression and metastasis. In NAXIVA no participants progressed from non-metastatic to metastatic disease. Two patients did not proceed to surgery due to the progression of their known metastatic disease, suggesting that, consistent with results from the SURTIME trial [24], pre-surgical systemic therapy in M1 ccRCC may allow time for very aggressive disease to declare itself and ultimately enable patients to avoid inappropriate surgery. Reassuringly, the patterns of eventual VTT response at week 9 were mirrored on the 3-week safety MRI scan (originally included to ensure that any patient with clinically relevant progression could undergo surgery immediately); indeed, two patients had surgery expedited following a 3-week scan showing extension of VTT. If confirmed in future studies, this suggests that scans performed early during treatment could be a useful strategy as both a response prediction and reassuring safety feature for neoadjuvant systemic therapy [25, 26]. A shorter duration of neoadjuvant treatment may also be possible for an adequate response.

Patients with M0 and M1 disease and all levels of VTT, from those within the RV only to those with VTT extending to the right atrium were included in NAXIVA because all were hypothesised to benefit from a reduction in VTT extent if axitinib treatment reduced the extent of surgery and the associated surgical morbidity. The broad inclusion criteria in a small feasibility study limits firm conclusions on each subgroup, but conversely allowed signal seeking from each stage of the disease which informs future trials. The positive results showing significant reductions in VTT length (regardless of M0 or M1 status) are clinically relevant as they are linked to subsequent changes in surgical approach in 7/17 patients (41.1%). Importantly, axitinib treatment resulted in less extensive surgery such as avoidance of open nephrectomy in favour of laparoscopic/robotic procedures, and reduced requirement for intrathoracic approaches, cardiac bypass or Pringle manoeuvre which are associated with significant morbidity [2]. Conversely, reduction from Level 2 to Level 1 VTT appear less significant in changing the surgery undertaken, while the patient is still exposed to drug toxicity. The Mayo levels at which downstaging of VTT make most clinical difference are levels 0, 1, 3 and 4, although further investigation would be prudent given the relatively small numbers of such patients investigated within NAXIVA. Although no unexpected perioperative complications were reported, future studies should specifically measure this using the EAU Intraoperative Adverse Incident Classification (EAUiaiC) [27].

In NAXIVA, axitinib was used, a potent TKI, with an established aggressive dose escalation regime which has previously been demonstrated to have proven effect in non-metastatic and metastatic ccRCC [15]. After 8 weeks of axitinib, 16.7% patients had a partial response (10% in M0 patients). This compares with 45.8% in the Phase 2 trial of Karam et al. where axitinib treatment was given for 12 weeks. This suggests that a longer period of treatment is needed for deeper response, although by 9 weeks 41.1% of patients had >30% response in the VTT, downstaging of which was the aim of NAXIVA, suggesting this was an adequate treatment duration to assess the endpoints of this trial. Interestingly, results from NAXIVA are superior to previous retrospective studies, 37.5% vs 14.9–32.9% reduction in Mayo Levels 1–4 [5–12]. Despite permissive product labels in advanced disease, VEGFR TKIs do appear less active in non-ccRCC [28], and we caution against extrapolation of the findings of NAXIVA to patients in whom there is not pre-treatment histological proof of ccRCC.

An important question is whether baseline information or that obtained early during treatment can be used to select patients that may benefit, or not, from a period of neoadjuvant treatment. Previous studies have identified a number of molecular, genetic and other factors correlating with response to TKI [29]. We saw similar trends in predictive markers of angiogenesis, immune infiltrate and proliferation to those seen in large scale published datasets [21, 23]. We reconfirmed ctDNA is challenging to detect in RCC [19] and our finding that detectable ctDNA at baseline generally predicts poor response to axitinib may be clinically relevant and warrants investigation in larger cohorts. Additionally, although previous studies have shown that TKI-related AEs may correlate with response [30], we showed that non-responders received a significantly lower total dose of axitinib and had a shorter duration of treatment, with responders having significantly lower maximum levels of proteinuria during treatment than non-responders. This highlights the importance of active management of TKI-related AEs during neoadjuvant treatment to ensure patients remain on drug to enable effective tumour control.

A limitation of NAXIVA is that axitinib is now used in combination with immunotherapy in the first-line metastatic setting, and only used as a single agent in subsequent lines of treatment. Coupled with our finding that the immune profile in non-responders is consistent with an exhausted and regulatory T-cell phenotype suggests future trials should evaluate combinations such as IO-TKI where there is potential to improve the response rate in patients unlikely to respond to TKI alone, and enable both rapid downstaging with the TKI component and immune priming which could have longer-term survival implications [31–33]. However, we hypothesise that the downstaging effect may not be significantly greater with an IO-TKI combination compared with TKI alone. In the Neoavax neoadjuvant study of 12 weeks of axitinib/avelumab, there was a 30% PR, compared with 43% in the 12-week axitinib neoadjuvant protocol of Karam et al. [15, 34]. In addition, none of 17 patients treated with three every-2-week doses of neoadjuvant nivolumab had a PR [35]. Future randomised studies should explore the impact on overall survival, differences in the extent of surgery and optimisation of treatment schedule and duration.

In conclusion, the results from NAXIVA showed the feasibility that systemic therapy, such as axitinib, can be used to downstage RCC VTT in 35% of patients and reduce the extent of surgery to a less morbid option in 41%. As newer combination therapies are associated with higher response rates in advanced ccRCC, the study of these combinations in patients with operable locally advanced disease should now be prioritised.

## Supplementary information

Supplementary methods

Supplementary figures

Study protocol

Sampling handling manual

Submission checklist

## Data Availability

The datasets generated and analysed during NAXIVA are available from the corresponding author following assessment of a brief research proposal which will form the basis of a data-sharing agreement. All reasonable requests will be granted.
