# Supplementary material for: A Phase II study of neoadjuvant axitinib for reducing the extent of venous tumour thrombus in clear cell renal cell cancer with venous invasion (NAXIVA)
Source: Br J Cancer. 2022 Jun 23;127(6):1051–60. doi: 10.1038/s41416-022-01883-7 (PMC9470559; doi:10.1038/s41416-022-01883-7)
Supplement: Supplementary file 1 — Supplementary methods [file 41416_2022_1883_MOESM1_ESM.docx]

**Supplementary material**

***MRI scan protocol***

MRI scans were acquired on 1.5 T scanners from different manufacturers depending on on-site availability. The protocol included an axial T_1_w dual-echo in-phase/opposed-phase gradient echo sequence, coronal and axial balanced steady-state free precession (bSSFP; T_2_/T_1_-weighted) sequences, breath-hold axial and coronal or sagittal T_2_w single-shot turbo spin echo (TSE) sequences, free-breathing respiratory-navigator-triggered axial T_2_w turbo spin echo TSE with fat suppression and spin-echo echo-planar imaging diffusion-weighted imaging (EPI DWI) with b-values of 0 and 600 s/mm^2^. Following the injection of 0.1 ml of 1M Gadobutrol (Gadovist, Bayer, Reading, UK) per kilogram of patient body weight, eight phases of a coronal multi-phase Dixon sequence were acquired with a temporal resolution of approximately 15s. Axial Dixon images were also acquired pre- and post-contrast. Apparent diffusion coefficient (ADC) maps were reconstructed from the DWI data using mono-exponential fitting as implemented by the scanner manufacturer. The individual technological capabilities at the participating sites dictated the exact implementation of the imaging protocol. As a reference, this section describes the protocol employed at Addenbrookes Hospital, Cambridge University Hospitals NHS Foundation Trust. All patient scans were acquired on a clinical 1.5 T MRI scanner (Discovery MR 450 or Optima MR450w, GE Healthcare, Waukesha, WI, USA). Supplementary table 5 details the imaging parameters as implemented at Addenbrooke’s Hospital.

**Supplementary tables**

**Table S1.** Overall disease burden RECIST response by M-status

|  | **M0** | | **M1** | |
| --- | --- | --- | --- | --- |
|  | **Week 3** | **Week 9** | **Week 3** | **Week 9** |
| Complete response | 0 | 0 | 0 | 0 |
| Partial response | 0 | 1 | 1 | 2 |
| Stable disease | 11 | 9 | 8 | 4 |
| Progressive disease | 0 | 0 | 0 | 2 |
| Non-evaluable, specify | 0 | 0 | 0 | 0 |
| Missing | 0 | 1 | 1 | 2 |
| Total | 11 | 11 | 10 | 10 |

**Table S2.** Changes in planned control of IVC or renal vein in patients with a reduction in level of control of IVC.

| **Patient** | **Surgery planned** | **Surgery performed** | **Outcome for level of venous control** |
| --- | --- | --- | --- |
| N0101 | Supra-hepatic (supradiaphragmatic) | Supra-hepatic (infradiaphragmatic) | Improvement |
| N0201 | Infra-hepatic (IVC clamping with no liver mobilisation) | Thrombus - Milked back into renal vein and side clamped | Improvement |
| N0103 | Retro-hepatic (liver mobilisation and clamping below hepatic veins) | Infra-hepatic (IVC clamping with no liver mobilisation) | Improvement |
| N0904 | Infra-hepatic (IVC clamping with no liver mobilisation) | Thrombus - Milked back into renal vein and side clamped | Improvement |
| N0205 | Supra-hepatic (supradiaphragmatic) | Retro-hepatic (liver mobilisation and clamping below hepatic veins) | Improvement |

**Table S3.** Details of planned and performed surgery

|  | **Planned** | **Performed** |
| --- | --- | --- |
| **Surgical approach** |  |  |
| Minimally invasive surgery | 1 | 3 |
| Open Surgery | 16 | 14 |
| **Open surgical incision** |  |  |
| Flank | 0 | 0 |
| Subcostal | 3 | 4 |
| Midline Laparotomy | 2 | 2 |
| Rooftop | 7 | 5 |
| Mercedes Benz incision | 1 | 2 |
| Thoraco-abdominal | 3 | 1 |
| Midline Sternotomy | 0 | 0 |
|  |  |  |
| **Control of IVC/RV** |  |  |
| Thrombus milked back into renal vein and side clamped | 4 | 7 |
| Infra-hepatic (IVC clamping, no liver mobilization) | 7 | 5 |
| Retro-hepatic (liver mobilization, clamping below hepatic veins) | 4 | 4 |
| Retro-hepatic (liver mobilization, clamping above hepatic veins) | 0 | 0 |
| Supra-hepatic (infradiaphragmatic) | 0 | 1 |
| Supra-hepatic (supradiaphragmatic) | 2 | 0 |
| **IVC/RV maneuvers** |  |  |
| Clamping of contralateral renal vein* | 10 | 8 |
| IVC Ligation | 2 | 1 |
| Caval patch/replacement | 1 | 4 |
| **Adjuvant thrombus related procedures** |  |  |
| Veno venous bypass | 1 | 0 |
| Open heart surgery | 0 | 0 |
| Cardio-pulmonary bypass | 0 | 0 |
| Hypothermic cardiac arrest | 1 | 0 |
| **Non-thrombus related procedures** |  |  |
| Ipsilateral adrenalectomy | 11 | 10 |
| Extended lymph node dissection | 3 | 1 |
| Lymph node sampling | 6 | 7 |
| **Other specialities participating in surgery** |  |  |
| Vascular surgeon | 1 | 0 |
| Liver transplant surgeon | 3 | 3 |
| Hepato-Pancreato-Biliary surgeon | 3 | 4 |
| Cardiothoracic surgeon | 1 | 0 |

* Median duration of clamping of contralateral renal vein 22 minutes 30 seconds (range 10-63 minutes).

**Table S4**. Planned and performed incisions, matched by patient.

| **Patient** | **Incision planned** | **Incision performed** | **Outcome of planned versus performed incision** |
| --- | --- | --- | --- |
| N0101 | Mercedes Benz incision | Rooftop & Mercedes Benz incision | No change |
| N0102 | Rooftop | Mercedes Benz incision | No change |
| N0103 | Rooftop | Subcostal | Improvement |
| N0104 | Rooftop | Rooftop | No change |
| N0105 | Rooftop | Minimally invasive surgery | Improvement |
| N0106 | Rooftop | Rooftop | No change |
| N0201 | Subcostal | Subcostal | No change |
| N0202 | Subcostal | Subcostal | No change |
| N0204 | Subcostal | Subcostal | No change |
| N0205 | Midline Laparotomy | Rooftop | No change |
| N0601 | Minimally invasive surgery | Minimally invasive surgery | No change |
| N0605 | Rooftop | Rooftop | No change |
| N0606 | Rooftop | Rooftop | No change |
| N0901 | Midline Laparotomy | Midline Laparotomy | No change |
| N0902 | Thoraco-abdominal | Thoraco-abdominal | No change |
| N0904 | Thoraco-abdominal & Midline Laparotomy | Minimally invasive surgery | Improvement |
| N0905 | Thoraco-abdominal & Midline Laparotomy | Subcostal & Midline Laparotomy | Improvement |

**Table S5.** NAXIVA MRI Parameters. Field of view (FoV) was occasionally increased if needed due to patient size.

| Sequence | TE  [ms] | TR  [ms] | Flip  [°] | FoV  [mm] | resolution [mm^2^] | Slice/gap  [mm] | Orientation | Type | Comments |
| --- | --- | --- | --- | --- | --- | --- | --- | --- | --- |
| T1w FIESTA | 1.3 | 3.0 | 45 | 340 x 238 | 2.1 x 0.9 | 8 / 2 | Ax | 2D | BH |
| T1w FIESTA | 1.5 | 3.3 | 55 | 340 x 340 | 1.8 x 0.9 | 8 / 2 | Cor | 2D | BH |
| T2w SSFSE | 60 | N/A | 90/155 | 340 x 238 | 1.3 x 1.3 | 8 / 2 | Ax | 2D | BH |
| T2w SSFSE | 60 | N/A | 90/155 | 340 x 340 | 1.3 x 1.3 | 8 / 2 | Cor | 2D | BH |
| 2D SPGR dual echo | 2.1 / 4.3 | 150 | 60 | 340 x 238 | 1.3 x 2.7 | 8 / 2 | Ax | 2D | BH |
| T2w FRFSE | 75 | 1 breath | 90 / 160 | 360 x 288 | 1.1 x 1.4 | 8 / 2 | Ax | 2D | RT, ETL 13, ASSET 2, Nex 4, FatSat |
| DWI | 49 | 2000–2250 | 90 / 180 | 380 x 380 | 4.8 x 3.0 | 8 / 2 | Ax | 2D | BH, b-values 0, 600 mm/s^2^, ASSET 2, Nex 4 |
| T1w Lava-Flex ±C | 2.1 / 4.3 | 6.4 | 12 | 400 x 320 x 32 | 1.3 x 1.8 | 6 / 0 | Ax | 3D | BH, pre- and post-contrast |
| T1w Lava-Flex ±C | 2.1 / 4.3 | 6.5–6.9 | 12 | 400 x 400 x 26 | 1.3 x 1.8 | 4 / 0 | Cor | 3D | BH, pre- and post-contrast, angiographic triggering, multi-phase (8 phases) |

Ax: axial, BH: breath hold, C: contrast-enhanced, Cor: Coronal, ETL: echo train length, NEX: number of excitations, RT: respiratory triggered (navigator), T1w: T1-weighted, T2w: T2-weighted.

**Supplementary figure legends**

**Figure S1**. Examples of VTT response to axitinib. (a) Reduction of the tumour thrombus length from above diaphragm (Mayo 4) to below hepatic inflow (Mayo 2). (b) Reduction of the tumour thrombus length from Mayo 2 to Mayo 1

**Figure S2.** Longitudinal lengths of IVC VTT above and below the ostium of the RV

**Figure S3.** a) Axitinib dose schedules (b.d.) per evaluable patient (N=21). b) Total axitinib dose taken according to Mayo Level or RECIST response; *p=0.03. c) Duration (days) of axitinib treatment according to Mayo Level response; *p=0.026. d) Duration (days) of axitinib treatment according to either Mayo Level or RECIST response; **p=0.007.

**Figure S4**. Dynamic tracking of adverse events over the course of NAXIVA.
Data are shown for those toxicities that affect more than one patient in at least one summary period. Toxicities are ordered by decreasing maximum % patients affected. Note that all data are shown here (i.e., any grading > 0); the reporting threshold as given above (10%) is indicated as a horizontal line. Any measurements that exceed 10% are shown in red.

**Figure S5.** Translational correlates of response status (microenvironment). (a) Representative image of biopsies co-stained for CD4 and FOXP3. Whole slides were scanned and quantified using automated computer image analysis on HALO for total CD4+ T cells (b) and CD4+ FOXP3+ T-regs (c). Area coverage was quantified using HALO for alpha smooth muscle actin (SMA) (d) and CD68 (e) (two tailed student t-test).

**Other supplementary material**

1. **NAXIVA trial protocol**
2. **NAXIVA trial translational sampling manual**
