# Supplementary material for: A Phase II study of neoadjuvant axitinib for reducing the extent of venous tumour thrombus in clear cell renal cell cancer with venous invasion (NAXIVA)
Source: Br J Cancer. 2022 Jun 23;127(6):1051–60. doi: 10.1038/s41416-022-01883-7 (PMC9470559; doi:10.1038/s41416-022-01883-7)
Supplement: Supplementary file 2 — Supplementary figures [file 41416_2022_1883_MOESM2_ESM.pptx]

## Slide 1
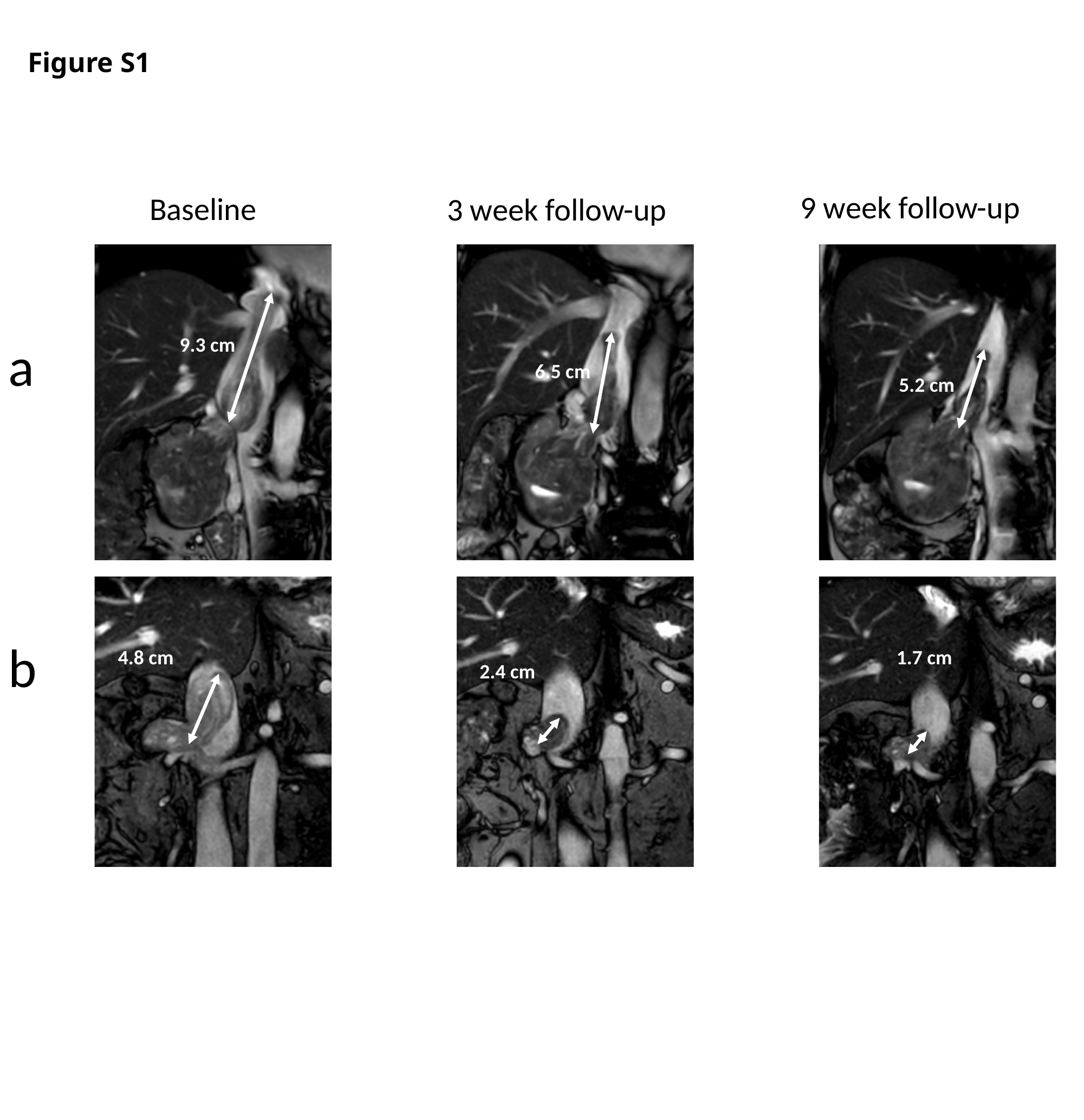

# Figure S1
9 week follow-up
Baseline
3 week follow-up
6.5 cm
5.2 cm
9.3 cm
a
1.7 cm
4.8 cm
2.4 cm
b

## Slide 2
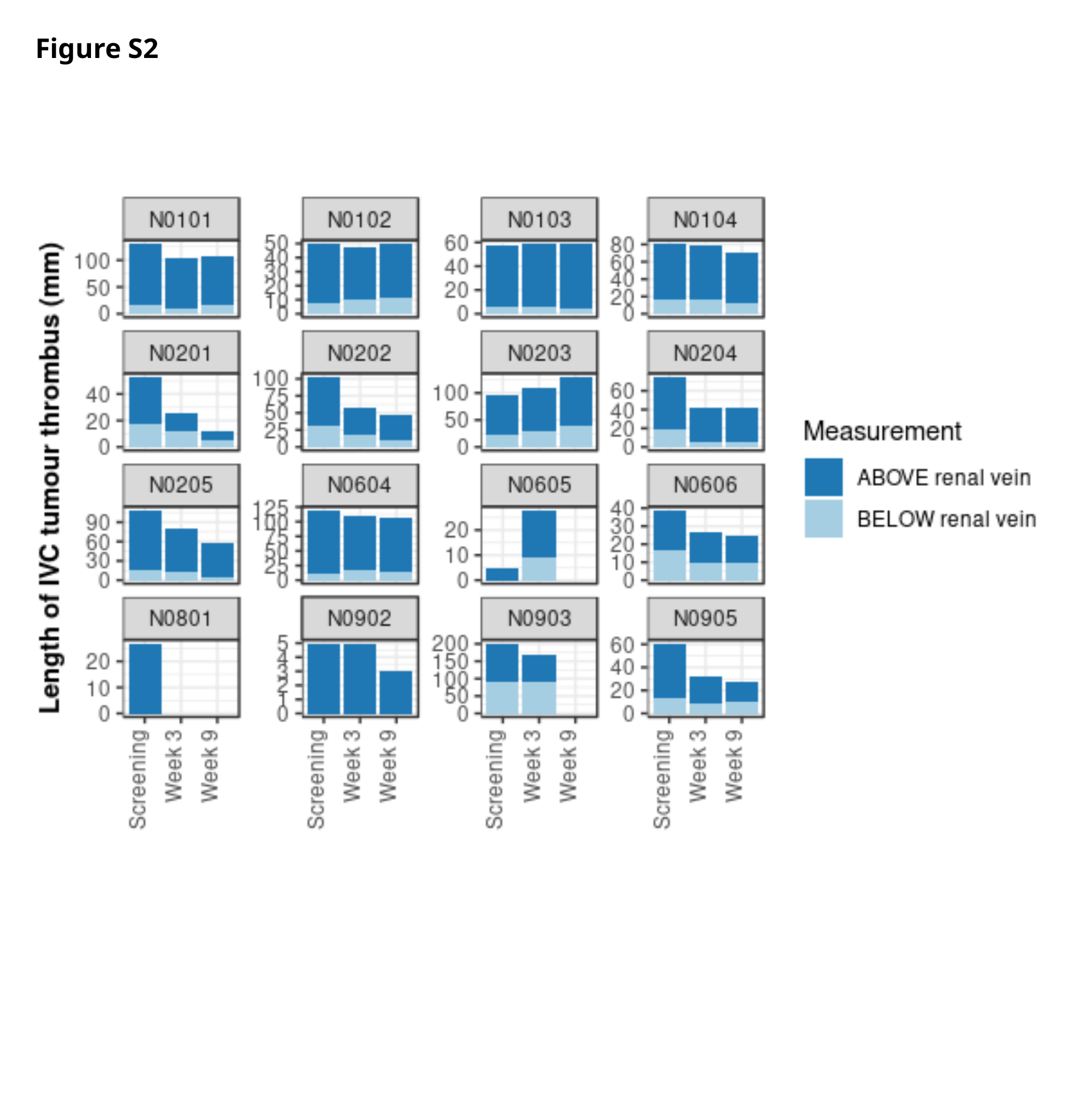

# Figure S2

## Slide 3
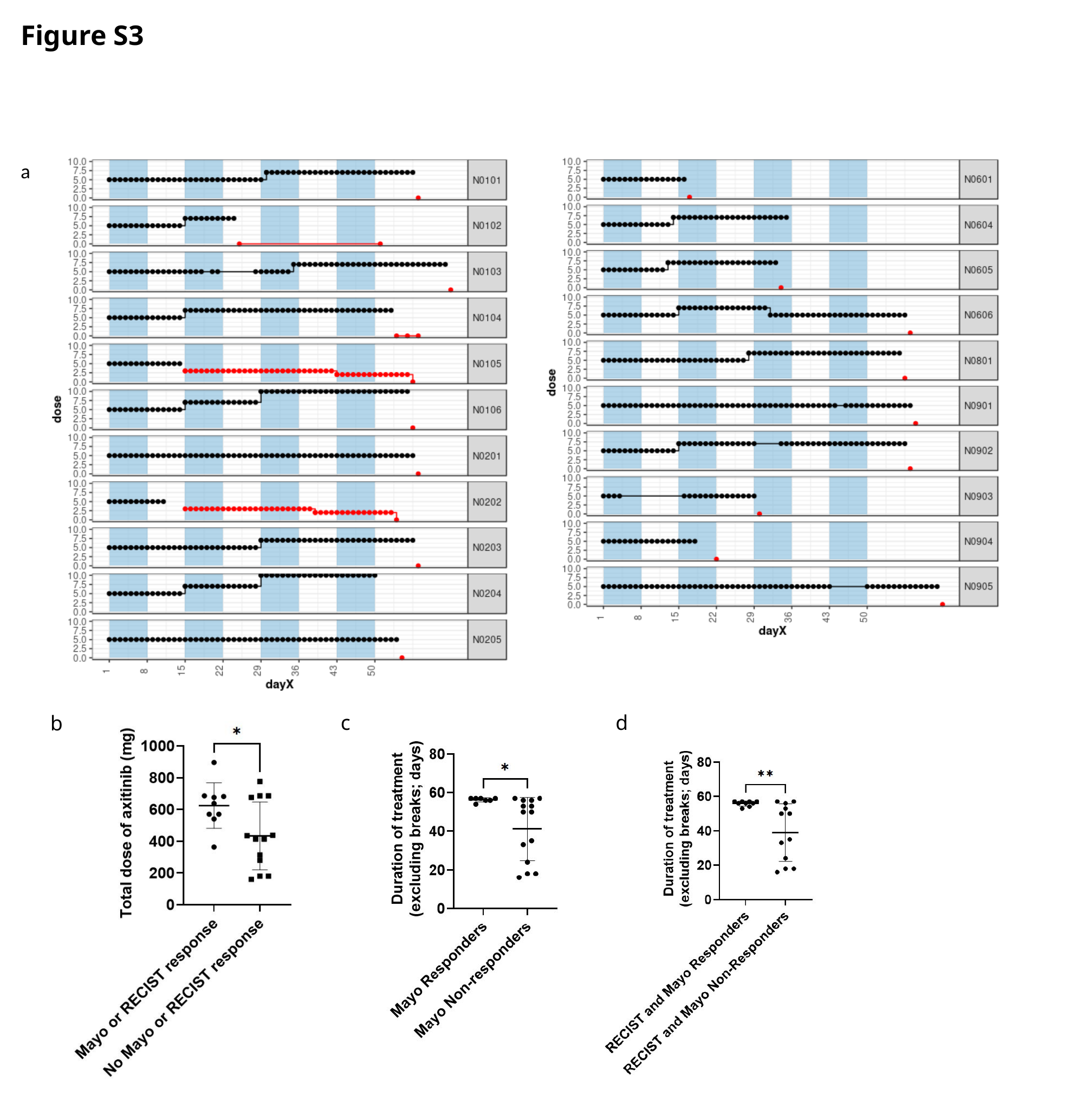

# Figure S3
a
c
d
b

## Slide 4
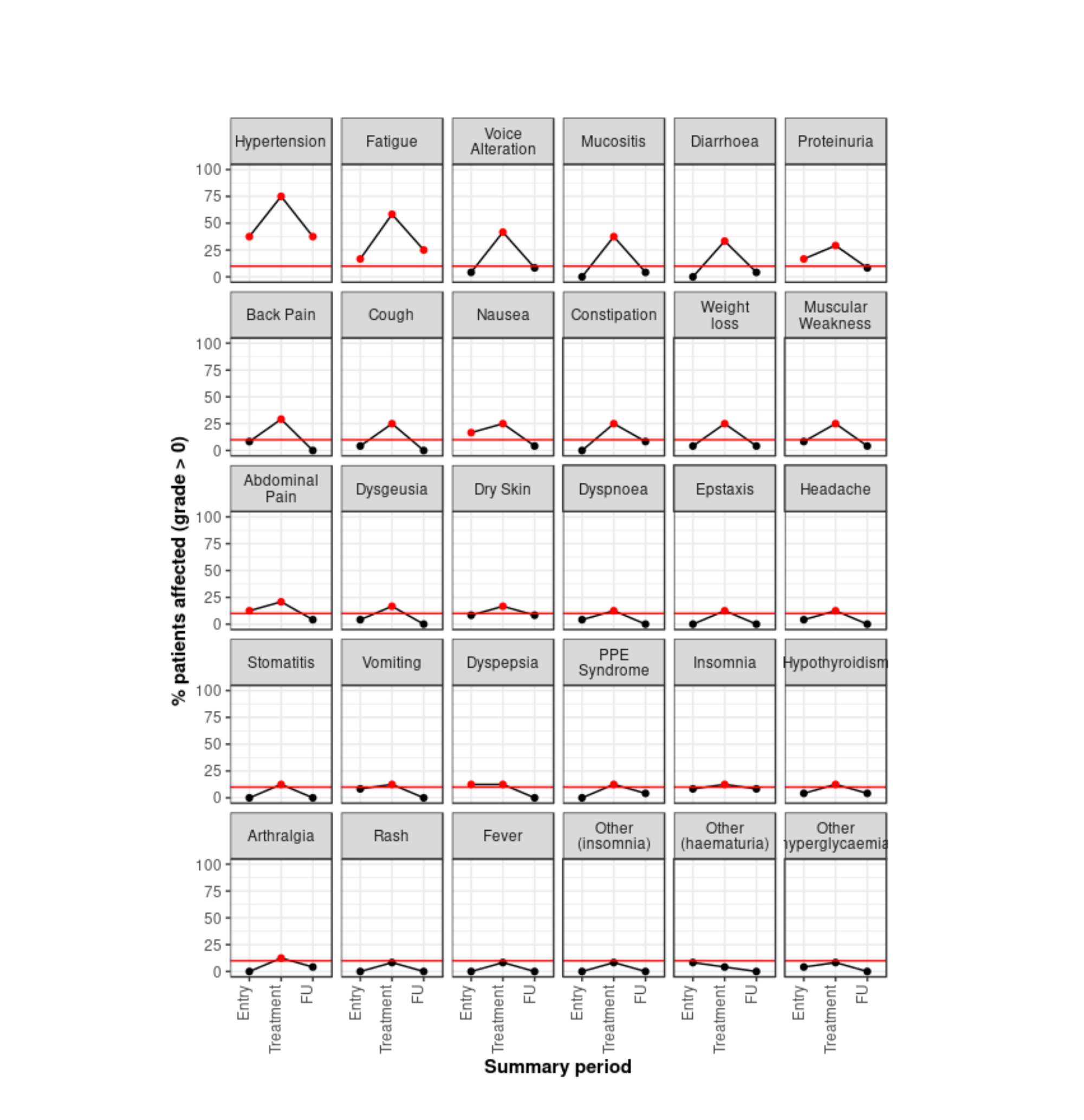

# Figure S4

## Slide 5
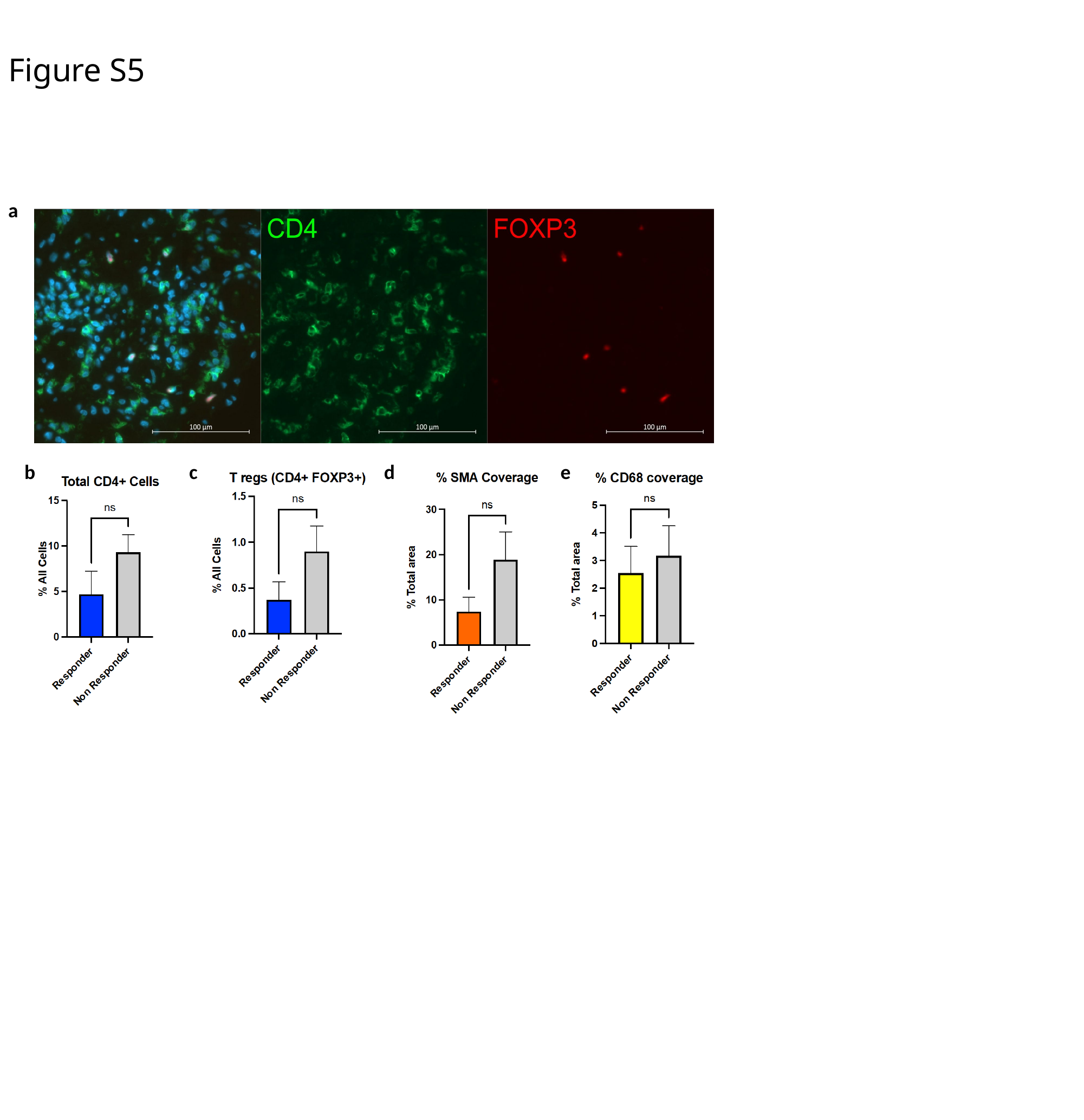

# Figure S5
a
b
c
d
e
