# Supplementary material for: A Phase II study of neoadjuvant axitinib for reducing the extent of venous tumour thrombus in clear cell renal cell cancer with venous invasion (NAXIVA)
Source: Br J Cancer. 2022 Jun 23;127(6):1051–60. doi: 10.1038/s41416-022-01883-7 (PMC9470559; doi:10.1038/s41416-022-01883-7)
Supplement: Supplementary file 3 — Study protocol [file 41416_2022_1883_MOESM3_ESM.pdf]

NAXIVA

PHASE II NEOADJUVANT STUDY OF AXITINIB FOR REDUCING EXTENT OF VENOUS TUMOUR THROMBUS IN CLEAR CELL RENAL CELL CANCER WITH VENOUS INVASION (NAXIVA)

Version number: 2.0 Date: 6th June 2018

Main Sponsor: Common Services Agency (CSA)

Funders: Pfizer Limited UK

Trial Coordinating Centre: Scottish Clinical Trials Research Unit (SCTRU),
NHS National Services Scotland, Edinburgh.

**ISRCTN:**	ISRCTN96273644
**EudraCT Number:**	2017-000619-17
**REC reference:**	17/EE/0240

Protocol authorised by:

Name: Mr Grant Stewart
Role in Trial: Chief Investigator

Signature: 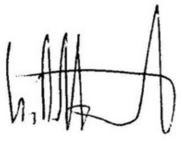 Date: 6th June 2018

KEY CONTACTS

Chief Investigator
Mr Grant Stewart
University of Cambridge
Academic Urology Group, Box 43,
Addenbrooke's Hospital, Cambridge Biomedical Campus, Hill's Road, Cambridge, CB2 0QQ

Co-Chief Investigator
Professor Robert Jones
University of Glasgow
Suite 3A, Beatson West of Scotland Cancer Centre, 1053 Great Western Road, Glasgow, G12 0YN

Translational Lead
Dr Sarah Welsh
University of Cambridge
Department of Oncology, Box 143, Addenbrooke's Hospital, Hill's Road, Cambridge, CB2 0QQ

Sponsor
The Common Services Agency
NHS National Services Scotland, Gyle Square, 1 South Gyle Crescent
Edinburgh, EH12 9EB

Funder
Pfizer Limited UK
Walton Oaks, Dorking Road, Walton-on-Hill
Tadworth, Surrey, KT20 7NS

Trial Coordination
Scottish Clinical Trials Research Unit
NHS National Services Scotland
Gyle Sqaure, 1 South Gyle Crescent, Edinburgh EH12 9EB
NSS.NAXIVA@nhs.net

TRIAL DEVELOPMENT GROUP

Mr Grant Stewart	University of Cambridge
Dr James Larkin	The Royal Marsden Hospital
Professor Robert Jones	University of Glasgow
Professor Thomas Powles	Bart's Cancer Institute
Mr Grenville Oades	Beatson West of Scotland Cancer Centre
Mr Michael Aitchison	The Royal Free Hospital
Professor David Nicol	The Royal Marsden Hospital
Dr Kate Fife	Addenbrooke's Hospital, Cambridge
Dr Iosif Mendichovszky	University of Cambridge
Dr Ferdia Gallagher	Addenbrooke's Hospital, Cambridge
Dr Sarah Welsh	University of Cambridge
Alison Fielding	Consumer Representative
Mr Robert Hill	SCTRU
Mrs Tracy McEleney	SCTRU
Mrs Niki Couper	SCTRU

Any questions relating to this protocol should be addressed in the first instance to the NAXIVA Team within SCTRU:

Email: NSS.NAXIVA@nhs.net
Telephone: 0131 275 7178
Fax: 0131 275 7512

SYNOPSIS

Protocol ID	NAXIVA
Protocol Title	Phase II **N**eoadjuvant study of **AXI**tinib for reducing extent of venous tumour thrombus in clear cell renal cell cancer with **V**enous inv**A**sion (NAXIVA)
Development Phase	Phase II Feasibility
Primary Endpoint	The percentage of evaluable patients with an improvement in the Mayo classification. The Mayo Classification will be assessed using the MRI/CT abdomen scans at screening and at week 9, prior to surgery.<br><br>Mayo Classification;<br>• Level 0: thrombus limited to the renal vein<br>• Level 1: into IVC <2cm from renal vein ostium level<br>• Level 2: IVC extension >2cm from renal vein ostium and below hepatic vein<br>• Level 3: thrombus at the level of or above the hepatic veins but below the diaphragm<br>• Level 4: thrombus extending above the diaphragm.
Secondary Endpoints	% change in surgical approach.<br>% change in venous tumour thrombus (VTT) height.<br>Response rate (RECIST).<br>Evaluation of morbidity assessed by Clavian-Dindo classification.
Study Design	NAXIVA is a single arm, single agent, open label, phase 2 feasibility study of axitinib in patients with both metastatic and non-metastatic renal cell carcinoma of clear cell histology prior to nephrectomy and thrombectomy.
Patient Accrual	20 patients will be recruited over a 24 month period at 7 sites across the UK.
Interim Analysis	Will be performed after thirteen patients have been recruited. If no patients show an improvement in their Mayo classification the trial will be stopped for futility.

TABLE OF CONTENTS

1. INTRODUCTION 7
1.1. BACKGROUND 7
1.2. INVESTIGATIONAL MEDICINAL PRODUCT 7
1.3. PRE-CLINICAL DATA 7
1.4. CLINICAL DATA 7
1.5. TRIAL RATIONALE 9
2. TRIAL OBJECTIVES 11
3. TRIAL DESIGN 11
3.1. GENERAL DESIGN 11
3.2. INCLUSION CRITERIA 11
3.3. EXCLUSION CRITERIA 12
3.4. ENDPOINTS 13
4. TREATMENT 14
4.1. TREATMENT SCHEDULE 14
4.2. DOSE MODIFICATIONS 14
4.3. DURATION OF TREATMENT 16
4.4. CONCOMITANT THERAPY 16
4.5. COMPLIANCE WITH TREATMENT 17
4.6. DRUG SUPPLIES AND LABELLING 18
4.7. DRUG STORAGE ACCOUNTABILITY 18
5. ASSESSMENT OF EFFICACY 20
5.1. TREATMENT AND EXAMINATION SCHEDULE 20
5.2. SCHEDULE OF ASSESSMENTS 21
5.3. WITHDRAWAL OF SUBJECTS 27
6. TRANSLATIONAL RESEARCH 27
6.1. CIRCULATING TUMOUR DNA (CTDNA) 27
6.2. METABOLOMICS 27
6.3. PREDICTIVE BIOMARKER STUDIES 28
6.4. COMPARATIVE STUDY OF IVC VTT IN UNTREATED AND TREATED PATIENTS 28
6.5. IMAGING 28
6.6. IMMUNE ANALYSIS 28
7. PHARMACOVIGILANCE 29
7.1. DEFINITIONS 29
7.2. RECORDING OF ADVERSE EVENTS 29
7.3. RECORDING AND REPORTING OF SERIOUS ADVERSE EVENTS 30
7.4. DEVELOPMENTAL SAFETY UPDATE REPORT 31
7.5. PREGNANCIES 31
8. DATA MANAGEMENT 31
8.1. DATA COLLECTION 31
8.2. RECORD KEEPING AND ARCHIVING 31

9. STATISTICS 31
9.1. SAMPLE SIZE 31
9.2. ANALYSIS PLAN 32
9.3. END OF STUDY 32
10. ACCESS TO SOURCE DATA/ DOCUMENTS 32
11. QUALITY CONTROL AND QUALITY ASSURANCE 32
11.1. MONITORING VISITS 33
11.2. DATA MONITORING AND ETHICS COMMITTEE 33
11.3. TRIAL STEERING COMMITTEE 33
12. ETHICAL CONSIDERATIONS 33
12.1. PATIENT CONFIDENTIALITY 33
12.2. INFORMED CONSENT 34
13. RESEARCH GOVERNANCE 34
14. FINANCING AND INSURANCE 35
15. PUBLICATION POLICY 35
REFERENCE LIST 36
APPENDIX 1 – ECOG AND KARNOFSKY PERFORMANCE SCORES 38
APPENDIX 2 – CAUSALITY 39
APPENDIX 3A – INVESTIGATOR STATEMENT (SCTRU COPY) 40
APPENDIX 3B – INVESTIGATOR STATEMENT (INVESTIGATOR COPY) 41
APPENDIX 4- ASSOCIATED DOCUMENTS 42
APPENDIX 5– THE PRINCIPLES OF ICH GOOD CLINICAL PRACTICE 43

GLOSSARY OF ABBREVIATIONS

AE	Adverse Event
AIDS	Acquired Immunodeficiency Syndrome
ALT	Alanine Transaminase
AR	Adverse Reaction
AST	Aspartate Transaminase
BID	Twice a day
BP	Blood Pressure
ccRCC	Clear Cell Renal Cell Cancer
CI	Chief Investigator
CRF	Case Report Form
CRP	C-Reactive Protein
CRUK	Cancer Research United Kingdom
CSA	Common Services Agency
CT	Computed Tomography
CTCAE	Common Toxicity Criteria for Adverse Events
ct DNA	Circulating Tumour DNA
DCE	Dynamic Contrast Enhanced
DMC	Data Monitoring Committee
DWI	Diffusion Coefficient
EDTA	Ethlenediaminetetraacetic Acid
EMT	Epithelial Mesenchymal Transition
EU	European Union
GCP	Good Clinical Practice
GDPR	General Data Protection Regulation
GP	General Practitioner
HIV	Human Immunodeficiency Virus
ICH	International Conference on Harmonisation
IC50	50% Inhibitory Concentration
IMP	Investigational Medicinal Product
IVC	Inferior Vena Cava
LDH	Lactate Dehydrogenase
M0	Non-metastatic
M1	Metastatic
MHRA	Medicines and Healthcare products Regulatory Agency
mRCC	Metastatic Renal Cell Carcinoma
MRI	Magnetic Resonance Imaging
MSKCC	Memorial Sloan Kettering Cancer Centre
NSCLC	Non Small Cell Lung Cancer
ORR	Objective Response Rate
PBS	Phosphate Buffered Saline
PCR	Protein: Creatinine Ratio
PDGF-R	Platelet-Derived Growth Factor Receptors
PI	Principal Investigator
PIL	Patient Information Leaflet
PMBC	Peripheral Blood Mononuclear Cells
PRES	Posterior Reversible Encephalopathy Syndrome
PSA	Prostrate-specific antigen
QP	Qualified Person
RCC	Renal Cell Cancer
R&D	Research and Development
REC	Research Ethics Committee
RECIST	Response Evaluation Criteria in Solid Tumours
SAE	Serious Adverse Event

SCTRU	Scottish Clinical Trials Research Unit
SDV	Source Data Verification
SmPC	Summary of Product Characteristics
SOP	Standard Operating Procedures
SUSAR	Suspected Unexpected Serious Adverse Reaction
TKI	Tyrosine Kinase Inhibitor
TMG	Trial Management Group
TSC	Trial Steering Committee
TSH	Thyroid Stimulating Hormone
TTP	Thrombotic Thrombocytopenic Purpura
TV	Tumour Thrombus Volume
UKCRC	United Kingdom Clinical Research Collaboration
ULN	Upper Limits of Normal
VEGF	Vascular Endothelial Growth Factor
VEGFR	Vascular Endothelial Growth Factor Receptor
VTT	Venous Tumour Thrombus

1. INTRODUCTION

1.1. Background

Venous tumour thrombus (VTT) extension into the renal vein and/or inferior vena cava (IVC) occurs in 4-15% cases of renal cell cancer (RCC) (1). If the patient is fit enough to undergo surgery, in an attempt to excise all disease with curative intent, the surgical risk is high due to the need to perform a cavotomy and/or open heart surgery to excise the VTT (5-15% mortality) (1). Complications are established to increase with the height of the VTT (1). After such surgery, the 5 year survival rates are poor; ~40-65% in non-metastatic RCC to 0-17% for patients with concomitant metastatic RCC (2, 3). As such, the concept of using targeted therapies, which are standard of care in metastatic RCC, to downstage the VTT prior to extirpative surgery is appealing. If it were possible to reduce the extent of the surgery an individual patient with RCC VTT required it is likely morbidity and mortality would be reduced and potentially survival time improved.

1.2. Investigational Medicinal Product

Axitinib is a potent oral VEGFR2 and 3 inhibitor at picomolar concentrations and VEGFR1, PDGFRs and c-KIT inhibitor at low nanomolar concentrations. In phase II clinical trials, axitinib has shown efficacy in sorafenib and cytokine refractory mRCC patients. A recently reported phase III trial, showed superiority over sorafenib as second line therapy (4), leading to the licensing of axitinib in this indication by the United States Food and Drug Administration (FDA) agency in January 2012. Axitinib was subsequently licensed for the same indication by the European Medicines Agency. Furthermore, the efficacy of axitinib is under evaluation in other tumour types, and has shown activity in lung (5), thyroid (6), and pancreatic (7) cancers, and melanoma (8).

1.3. Pre-Clinical Data

In vitro, axitinib inhibits cellular phosphorylation of VEGFR2 and 3 with an IC50 of about 0.2 nmol/L. It has a higher IC50 for VEGFR1 (1.2 nmol/L), PDGFR-β (1.6 nmol/L), PDGFR-α (5 nmol/L) and c-KIT (1.7 nmol/L). It reduces phosphorylation of downstream signalling molecules mediated by vascular endothelial growth factor (VEGF) in a rapid and dose-dependent manner, including Akt, endothelial nitric oxide synthase and extracellular signal regulated kinase (ERK).
In mouse xenografts, twice daily oral axitinib inhibited the primary tumour and controlled metastases in human melanoma (M24mwt), colorectal cancer (HCT-116) and RCC (SN12C) models. Within axitinib treated tumours, there was reduced CD31 and Ki-67 staining and increased caspase-3 staining. Microscopic examination of tumour vasculature in preclinical models of pancreatic islet cell tumours and Lewis lung carcinomas has shown that axitinib reduces patency and flow. Axitinib has its greatest effects on endothelial cells and fenestrated vessels, resulting in normalisation of the surviving tumour vasculature.

1.4. Clinical Data

1.4.1 Efficacy Data

Phase I
In an initial phase I study, 3 of 36 patients had partial responses determined by Response Evaluation Criteria in Solid Tumours (RECIST) (9) including 2 patients with RCC and 1 patient with adenoid cystic carcinoma (10). In a Japanese phase I trial, none of 12 recruited patients had a partial response but 3 patients had stable disease at 24 weeks, seen in one

patient each with colorectal carcinoma, thymic cancer and non-small cell lung cancer (NSCLC) (11).

Phase II: Axitinib in Cytokine-refractory Metastatic RCC

In a single arm, open label, phase II trial in cytokine-refractory mRCC, axitinib was administered at a starting dose of 5mg twice daily in the fasted state as 28 day treatment cycles until disease progression or significant toxicity (12). Fifty-two patients were recruited with a median age of 59 years and performance status 0 or 1. Forty-nine patients (95%) had previously undergone a nephrectomy and none of the patients had previously received tyrosine kinase inhibitors (TKIs). All patients had clear cell histology, except one patient who had papillary carcinoma. The objective response rate (ORR) was 44.2% (95% CI: 30.5-58.7%); there were 2 complete and 21 partial responses. Twenty-two patients had stable disease for at least 8 weeks, including the patient with papillary carcinoma. Four patients had early progression and 3 patients could not be assessed for response. The median thrombotic thrombocytopenic purpura (TTP) was 15.7 months and median overall survival was 29.9 months. The median duration of axitinib therapy was 9.4 months and median dose was 8.83 mg/day, as 15 patients had dose reductions for adverse events (AE) including fatigue, hypertension and diarrhoea. Axitinib was discontinued due to adverse events in ten patients. The most frequent grade 1-4 axitinib related adverse events were diarrhoea, hypertension, fatigue, nausea and hoarseness. The most common grade 3-4 adverse events were hypertension (15.4%), diarrhoea (9.6%) and fatigue (7.7%). Thirty patients had axitinib induced hypertension, eight of whom had grade 3-4 hypertension. Hypertension was resistant to anti-hypertensive therapy in 8 patients, seven of whom had hypertension at baseline. There were no detectable haematological toxicities. Four patients had treatment related proteinuria which resolved once axitinib was stopped.

Phase II: Axitinib in Sorafenib Refractory Metastatic RCC

In a single arm, open label, phase II study, patients with sorafenib refractory mRCC received a starting dose of axitinib 5mg twice daily with food until disease progression or unmanageable toxicity (13). Sixty-two patients were recruited; all had undergone prior nephrectomy and 59 patients had clear cell /mixed histology. Median age was 60 years and all patients were performance score of 0 or 1. The most common prior therapies in addition to sorafenib were sunitinib and cytokine therapy, received by 14 (22.6%) and 38 (61.3%) patients, respectively.

The ORR was 22.6% (95% CI 12.9-35.0%), with 14 patients experiencing a partial response and no complete responses. Tumour responses were observed in patients who received 5 mg twice daily or higher, and also in patients whose dose was reduced to below 5 mg twice daily. Patients who had a partial response received doses ranging from <4mg to 9-10mg twice daily. Eleven patients (17.8%) had stable disease. The median progression-free survival was 7.4 months (95% CI 6.7-11.0 months) and median overall survival was 13.6 months (95% CI 8.4-18.8 months).

The most common grade 3-4 adverse events included hypertension (16.1%), fatigue (16.1%), hand-foot syndrome (16.1%), dyspnoea (14.5%) and diarrhoea (14.5%). Twelve patients stopped axitinib due to treatment related adverse events. Two patients developed congestive heart failure, both of whom had a history of cardiovascular disease, and two patients had cerebral haemorrhages during the study. One of the latter patients was subsequently diagnosed with a cerebral metastasis at the site of haemorrhage. Most haematological toxicities were mild or moderate (grade 1 or 2) except grade 3 lymphopaenia experienced by 9 of 55 evaluable patients (16.4%).

1.4.2 Safety Data

Phase I

The primary dose limiting toxicity in the initial phase I trial was hypertension (10); in most cases it responded to anti-hypertensive therapy and resolved after axitinib was stopped. The incidence and severity of hypertension was dose-dependent, all patients with axitinib-induced hypertension at dose 5mg twice daily were managed with standard anti-hypertensives. Prior to blood pressure monitoring two patients had uncomplicated seizures in the absence of brain metastases at doses 10mg and 20mg twice daily, these may have been related to hypertensive crises.
Three bleeding events were reported during the phase I study. A patient with central NSCLC had a fatal haemoptysis attributed to axitinib. A patient with peripheral NSCLC had grade 1 haemoptysis while taking axitinib which was subsequently stopped. Two weeks later the patient had grade 4 haemoptysis and died, this patient's death was reported as secondary to disease progression and concurrent infection. Finally, there was 1 episode of grade 1 rectal bleeding.
Asymptomatic proteinuria was detected in seven of the first ten patients. Consequently, patients with proteinuria >0.5gram/24 hours were not recruited and treatment reviews were required for all patients with proteinuria ≥1gram/24 hours. These amendments reduced the incidence and severity of proteinuria.

Thrombocytopenia was the only haematological toxicity in the phase I trial, with grade 2 thrombocytopenia affecting one patient taking 20mg twice daily.

Phase II
The commonest non-haematological adverse events grade 1-4 reported in phase II axitinib monotherapy trials were hypertension, fatigue, diarrhoea, anorexia, nausea and hoarseness. The main grade 3-4 adverse events were similar, except hand-foot syndrome and proteinuria were also reported. Most adverse events were manageable. Hypertension usually responded to anti-hypertensive therapy and resolved once axitinib was stopped.
The starting dose in all but one of the phase 2 studies conducted to date was 5mg twice daily of axitinib. In one metastatic breast cancer study, axitinib dose was titrated up from a starting dose of 5mg twice daily in 1–3mg increments in patients tolerating axitinib. Those subjects who could tolerate axitinib with no adverse events related to axitinib above CTCAE (14) grade 2 for consecutive 2 week periods were permitted to increase their dose step-wise to 7mg twice daily and then to 10mg twice daily, unless their BP was >150/90mm Hg or the subject was receiving antihypertensive medication. All studies ongoing at the time of writing allow axitinib dose reductions to as low as 2mg twice daily for treatment-related adverse events. Except for an increase in hand-foot syndrome and slight increase in the incidence of hypertension, it appears that patients whose dose is titrated to between 6-10mg twice daily doses do not experience increased toxicities if they have previously tolerated 5mg twice daily starting dose. Axitinib dose titration will be permitted within NAXIVA, consistent with previous clinical trial protocols and the drug development programme for axitinib.

1.5. Trial Rationale

There is no level I or II evidence of neoadjuvant studies of targeted therapies in non-metastatic RCC VTT or upfront therapies in metastatic RCC VTT. There is level III evidence of mixed populations of RCC VTT patients (including both metastatic and non-metastatic patients) treated with a heterogeneous group of drug treatments (15, 16). The results of these studies suggest that regression in VTT is limited to sunitinib therapy (seen in 3/12=25% patients; regression not seen for bevacizumab, temsirolimus or sorafanib). With sunitinib treatment none of the patients (0/12) had an increase in the VTT level (15).

A recent prospective phase II of 12 weeks neoadjuvant axitinib in ccRCC T2-T3b, has been reported by Karam and colleagues (17). All of the patients in this study were cT3a (i.e. there were no patients with VTT). It was possible to titrate all patients up to 10mg axitinib with no grade 4/5 toxicities. 100% of patient's tumours showed response (46% patients had partial

response, and 54% stable disease) and the median reduction in primary tumour diameter was 28% by week 12. The median tumour diameter reduced from 10cm to 6.9cm. The vast majority of reduction in tumour size had occurred at 7 weeks of axitinib treatment.

The results of these small studies in non-metastatic RCC patients suggest that neoadjuvant TKI treatment of RCC patients is safe. However, the effect of these drugs on the extent of the VTT and the surgical approach that must be applied has not been confirmed. Authors in this field agree that a prospective trial is required to answer this important clinical question (15,16,18).

In NAXIVA we will study the response of VTT to axitinib. The primary outcome is percentage of patients with reduction in the Mayo Classification (19). This study will address the feasibility of patient recruitment in this setting. In order to ensure adequate recruitment both metastatic and non-metastatic patients will be recruited to assess the effect of axitinib therapy on the extent of the tumour thrombus, rather than to primarily assess a prolonged survival. A reduction in the extent of the VTT, as assessed by the Mayo classification, will potentially result in less extensive and less morbid surgical approach with immediate patient benefits of reduced operative mortality/morbidity, potential shorter hospital stay and shorter recuperation period to return to full activities of daily living. There is also potential for a longer disease free survival in patients treated with axitinib prior to nephrectomy and tumour thrombectomy. The risks of the treatment of patients with VTT with axitinib over an 8 week period include progression of their disease (from operable to non-operable or non-metastatic to metastatic). However, based on the studies outlined above there is no evidence that progressive disease will occur over this period of time. Treating patients with axitinib over an 8 week period may also increase the incidence of adverse events which could have a significant impact on the patient's fitness for surgery. This risk will be minimised as dose reduction and axitinib cessation will be managed as per section 4.

Axitinib has been chosen for this study as it is a potent TKI with proven effect in non-metastatic ccRCC (17). The dose regimen (starting at 5mg bd, increasing dose up to 10mg twice daily (BID), as rapidly as possible, as tolerated by patients) is a well established regimen in metastatic ccRCC and was shown to be effective in the previous non-metastatic ccRCC neoadjuvant study (18). 8 weeks has been chosen as the treatment duration as in the study by Karam et al maximum effect on the primary tumour size was observed by 7 weeks and no significant further advantage was seen by continuing treatment to 12 weeks.

Patients having biopsy proven ccRCC will be included in this study, as this is histological subtype of RCC shown to have a benefit from axitinib therapy. Patients with VTT confined to the renal vein (cT3a) will be included as they would benefit from a reduction in the extent of the VTT, regressing from the renal vein back into the kidney, as this would allow curative surgery by laparoscopic rather than more morbid open surgical approaches. Patients with more extensive IVC VTT (infrahepatic, intrahepatic or atrial) will be included, as for patients in each of these groups reducing the extent of the thrombus i.e. from intrahepatic to infrahepatic would potentially reduce extent of surgery and the associated surgical morbidity. Both non-metastatic and metastatic patients will be eligible for recruitment as both groups of patients are likely to benefit, in terms of reduction of level of VTT and surgical morbidity, if axitinib shows efficacy in this patient cohort. It is accepted that there may be different biological processes at play in these 2 cohorts. However, this will be further explored in a fully powered phase 3 randomised control trial, if this phase 2 trial is positive.

2. TRIAL OBJECTIVES

Aims: to assess the reduction in the Mayo Venous Tumour Thrombus Classification of patients with clear cell renal cell cancer who have a renal vein or inferior vena cava venous tumour thrombus treated with neoadjuvant (non-metastatic patients) or upfront (metastatic patients) axitinib therapy.

Primary outcomes:

- The percentage of evaluable patients with an improvement in the Mayo Classification. The Mayo Classification will be assessed using the MRI/CT abdomen scans at screening and at week 9, prior to surgery.

Mayo Classification;

- Level 0: thrombus limited to the renal vein
- Level 1: into IVC <2cm from renal vein ostium level
- Level 2: IVC extension >2cm from renal vein ostium and below hepatic vein
- Level 3: thrombus at the level of or above the hepatic veins but below the diaphragm
- Level 4: thrombus extending above the diaphragm.

Secondary outcomes:

- % change in surgical approach
- % change in VTT height
- Response rate (RECIST)
- Evaluation of surgical morbidity assessed by Clavian-Dindo classification

3. TRIAL DESIGN

3.1. General Design

NAXIVA is a single arm, single agent, open label, phase II feasibility study of axitinib in patients with both metastatic and non-metastatic renal cell carcinoma of clear cell histology. 20 patients will be recruited from multiple centres within the United Kingdom.

Patients who have signed informed consent and who have met all eligibility criteria will be registered into the trial.

The starting dose of axitinib will be 5mg BID and escalated to 7mg BID and then 10mg BID. A dose modification assessment will take place every 2 weeks in clinic during the 8 week pre-surgical treatment period and will be dependent on tolerability of treatment. Patients will follow an aggressive axitinib dose escalation process within the 8 week period to a maximum of 10mg BID. Patients should stop axitinib a minimum of 36 hours and a maximum of 7 days prior to surgery in week 9.

Blood, urine and tissue samples will be taken prior to and during therapy to evaluate biomarkers of treatment response. Nephrectomy and IVC tumour thrombectomy will be planned for all patients on the trial.
Response to axitinib in VTT, primary tumour and any RECIST measureable lesion will be correlated with changes in molecular markers.

Patients will be followed up in clinic at 6 & 12 weeks post surgery.

3.2. Inclusion Criteria

1. Age ≥ 18.

2. Histologically proven clear cell RCC.
3. Immediate resection of the primary tumour considered technically possible.
4. Suitable for and willing to undergo nephrectomy (either cytoreductive or with curative intent)
5. cT3b, cT3c, cT3a (main renal vein)
6. N0, N1, or Nx
7. M0, or M1
8. ECOG performance status 0 – 1
9. Urinalysis <2+ protein. If dipstick is ≥2+ then a 24-hour urine collection or urinary protein creatinine ratio (PCR) should be performed and the patient may enter NAXIVA only if urinary protein is <2g per 24 hours or PCR <200mg/mmol.
10. Serum creatinine ≤1.5xULN or estimated creatinine clearance ≥30mL/min as calculated using the Cockcroft-Gault (CG) equation
11. All female patients with reproductive potential must have a negative serum or urine pregnancy test within a maximum of 14 days prior to starting trial treatment.

3.3. Exclusion Criteria

1. For M1 patients: poor risk on Memorial Sloan Kettering Cancer Centre (MSKCC) score and deemed suitable for cytoreductive nephrectomy at time of enrolment.
2. Other invasive malignancy within the last 2 years. Patients with previous history of malignancies with a negligible risk of metastasis or death and treated with expected curative intent are eligible, for example but not exclusively :
    - Carcinoma in situ of the cervix.
    - Basal or squamous cell skin cancer.
    - Localized low to intermediate risk prostate cancer treated with curative intent and absence of prostate-specific antigen (PSA) relapse; or prostate cancer (Stage T1/T2a, Gleason ≤6 and PSA <10ng/mL) undergoing active surveillance and treatment naïve.
3. Women who are pregnant or are breastfeeding. Female patients must be surgically sterile, be postmenopausal, or must agree to use effective contraception during the period of therapy and up to 1 week after treatment.
   Male patients must be surgically sterile or must agree to use effective contraception during the period of therapy and for 6 months after completion of study drug (Patients who do not meet this will not be are not eligible).
4. Current signs or symptoms of severe progressive or uncontrolled hepatic, endocrine or pulmonary disease other than directly related to RCC.
5. Gastrointestinal abnormalities including: a. inability to take oral medication; b. requirement for intravenous alimentation; c. prior surgical procedures affecting absorption including total gastric resection; d. treatment for active peptic ulcer disease in the past 6 months; e. active gastrointestinal bleeding, unrelated to cancer, as evidenced by hematemesis, hematochezia or melena in the past 3 months without evidence of resolution documented by endoscopy or colonoscopy; f. malabsorption syndromes.
6. Current use or anticipated need for treatment with drugs that are known potent CYP3A4 inhibitors (see section 4.4, concomitant therapy).
7. Current use, or anticipated need for treatment with, drugs that are known CYP3A4 inducers or substrates for CYP1A2 (see section 4.4, concomitant therapy).
8. Requirement of anticoagulant therapy with oral vitamin K antagonists. Low-dose anticoagulants for maintenance of patency of central venous access device or prevention of deep venous thrombosis is allowed. Therapeutic use of low molecular weight heparin is allowed.
9. Active seizure disorder, spinal cord compression, or carcinomatous meningitis.

10. Any of the following within 12 months prior to study entry: myocardial infarction, uncontrolled angina, coronary/peripheral artery bypass graft, symptomatic congestive heart failure, cerebrovascular accident or transient ischemic attack.
11. Uncontrolled hypertension (>160/100 mmHg despite optimised antihypertensive treatment).
12. Known human immunodeficiency virus (HIV) or acquired immunodeficiency syndrome (AIDS)-related illness.
13. Total serum bilirubin ≥1.5 x ULN; Aspartate aminotransferase (AST) and alanine aminotransferase (ALT) ≥2.5 x ULN.
14. Neutrophil count < 1.0 x 10^9/L; platelet count < 100 x 10^9/L; Hb ≤ 90g/L
15. Known severe hepatic impairment (Child-Pugh class C)
16. Known hypersensitivity to axitinib or any of its excipients. Specifically patients with hereditary galactose intolerance, Lapp lactase deficiency or glucose-galactose malabsorption should not enter the study.

3.4. Endpoints

Primary:

- The percentage of evaluable patients with an improvement in the Mayo Classification.

Mayo Classification;

- Level 0: thrombus limited to the renal vein
- Level 1: into IVC <2cm from renal vein ostium level
- Level 2: IVC extension >2cm from renal vein ostium and below hepatic vein
- Level 3: thrombus at the level of or above the hepatic veins but below the diaphragm
- Level 4: thrombus extending above the diaphragm.

Secondary:

- % change in surgical approach
- % change in VTT height
- Radiological response (RECIST)
- Evaluation of morbidity assessed by Clavian-Dindo classification

Primary Safety Endpoints

Thirteen patients will be recruited at first; if at least 1 patient has an improvement in the Mayo classification a further 7 patients would be recruited (to the final total of 20 patients who have received study drug), to receive study drug. At least 3 patients showing reductions in Mayo score are required for a positive trial. The early stopping rules are:

(1) If a single M0 patient progresses to the point where surgery is no longer possible, then recruitment of new patients will be suspended following a review by the data monitoring committee (DMC).
(2) If a single M0 patient becomes M1 recruitment of new patients will be suspended following a review by the DMC.
(3) If in 3 patients the VTT extends but patients remain surgically resectable the trial will close. If the VTT has extended the patient will be expedited and operated on as soon as possible.

Where there is diagnostic doubt at site, the week 3 MRI abdomen scans (and CT chest for M0 patients) a central review will be conducted to determine if a patient has progressed as per RECIST 1.1 criteria. In addition those particular patient cases will be discussed on an individual basis by the trial steering committee (TSC).
The central review for final analysis will be conducted by radiologists based at Addenbrooke's Hospital.

4. TREATMENT

4.1. Treatment Schedule

Patients should start axitinib treatment on day 1, week 1 of the study and continue for 8 weeks.
Patients will be prescribed 1 pack (28 tablets) of 5mg BID at week 1. Patients will then be prescribed further axitinib every 2 weeks following the dose modification schedule detailed in section 4.2 (depending on toxicities and blood pressure). Doses should be taken appropriately 12 hours apart and patients should be instructed to take their doses at approximately the same time each day with or without food as per instruction. On clinic days only, patients will be advised to fast for 6 hours prior to their clinic visit.
Patients should be advised to stop axitinib treatment a minimum of 36 hours and maximum of 7 days prior to week 9 surgery and tissue collection.
If a patient vomits or misses a dose, an additional dose should not be taken. The next prescribed dose should be taken at the usual time. If the patient reports having missed a dose or vomiting, this should be recorded in the source documents and case report forms (CRFs).

4.2. Dose Modifications

Dose Level	Dose	Dispensed As
**+2**	**10mg BID**	**2 x 5mg tablets BID (2 x 5mg packs)**
**+1**	**7mg BID**	**1 x 5mg tablet BID + 2 x 1mg tablets BID (1x 5mg + 2 x 1mg packs)**
**0 (starting dose)**	**5mg BID**	**1 x 5mg tablet BID (1 x 5mg pack)**
**-1**	**3mg BID**	**3 x 1mg tablets BID (3 x 1mg packs)**
**-2**	**2mg BID**	**2 x 1mg tablets BID (2 x 1mg packs)**

Dose Escalation
Patients who tolerate axitinib with no adverse events related to study drug above CTCAE grade 2 for a consecutive 2 week period should follow the aggressive dose escalation schedule above and have their dose increased by one dose level to a maximum of 10 mg BID. The only exception to this would be if the patient's blood pressure (BP) is >150/90 mm Hg or the patient is receiving antihypertensive medication. If the patient is receiving antihypertensive medication and a dose escalation is clinically indicated this should be considered by the onsite clinical team.

Dose Interruption and Reduction
Patients experiencing non-haematological drug reactions greater than CTCAE Grade 2 or haematological reactions greater than CTCAE Grade 3 should undergo dose modification. The current version of the Summary of Product Characteristics (SmPC) should be used to assess whether any adverse event is attributable to the drug.
Axitinib should be stopped in the event of significant toxicity and restarted if appropriate when toxicities have resolved. If the patient has had an interruption of axitinib for more than 2 weeks in total or has had multiple interruptions, it must be discussed with the Chief Investigator (CI) before the patient restarts study drug.
All patients who are permanently removed from treatment for intolerable toxicity should receive a post treatment CT chest, abdomen and pelvis scan within 2 weeks of a decision to

stop treatment. The CT scan should be assessed according to RECIST 1.1 criteria, and the patient should revert to standard of care.

The criteria for dose modification for axitinib related adverse events are summarised in the table below:

Criteria for dose modification for axitinib- related adverse events (other than hypertension or proteinuria)

Related Adverse Events	Intervention
Any grade: bleeding where medical intervention is required	Temporarily interrupt the axitinib dose.
Any grade: posterior reversible encephalopathy syndrome (PRES)	Patients with signs or symptoms should temporarily interrupt or permanently discontinue axitinib treatment. In case of severe or persistent arterial hypertension and symptoms suggestive of posterior reversible encephalopathy syndrome, a diagnostic brain magnetic resonance image (MRI) should be considered. If PRES is confirmed, the patient should permanently discontinue axitinib.
Grade 1	Continue at same dose level
Grade 2	Continue at same dose level
Grade ≥3 non-haematologic treatment-related toxicity or Grade 4 haematologic toxicity (other than those below)	Interrupt dosing; re-start at one lower dose level as soon as improvement to CTCAE Grade≤2. If patient requires dose reduction below 2mg BID, contact SCTRU for discussion prior to implementation.
Grade 4 lymphopaenia	Continue at the same dose level at the discretion of the investigator.

Axitinib dose reduction guidance for hypertension

Degree of Blood Pressure Elevation			Management
Systolic Pressure		Diastolic Pressure	
2 consecutive BP readings show systolic pressure >150 mmHg	OR	2 BP readings separated by at least 1 hour show diastolic pressure >90 mmHg	If not on maximal antihypertensive treatment, institute new or additional antihypertensive medication and maintain dose of axitinib. If on maximal antihypertensive treatment, reduce axitinib to one lower dose level.
2 consecutive BP readings show systolic pressure >160 mmHg	OR	2 BP readings separated by at least 1 hour show diastolic >100 mmHg	Interrupt dosing *; adjust antihypertensive medication; as soon as BP is less than 150/90 mmHg, restart axitinib at one lower dose level.
Recurrent hypertension following previous dose reduction (2 consectutive BP readings show systolic pressure >150 mmHg)	OR	Recurrent dBP>90 mmHg (2 BP readings separated by at least 1 hour) following previous dose reduction.	Repeat axitinib dose reduction by one lower dose level. If a patient requires dose reduction below 2 mg BID, contact SCTRU for discussion.

* If dose is interrupted, patients receiving antihypertensive medications should monitor closely for hypotension.

Axitinib Dose reduction for Proteinuria

- If dipstick shows ≥2+ proteinuria, perform 24 hour urine collection or urinary PCR. Dosing may continue while waiting for test results.
- If < 2g proteinuria/24 hour or urinary PCR < 200mg/mmol is reported, continue dosing at the same dose level.
- If ≥ 2g proteinuria/24 hours or urinary PCR ≥ 200mg/mmol is reported, withhold dose and repeat 24 hour urine collection or urinary PCR (interval at investigator discretion) until proteinuria is < 2 g/24 hours or urinary PCR < 200mg/mmol. Restart axitinib at the same dose or one lower dose level at discretion of the investigator. Monitor renal function in accordance with standard practice.
- If ≥ 2g proteinuria/24 hours or urinary PCR ≥ 200mg/mmol is reported and the investigator deems this to be unrelated to axitinib, patients should restart axitinib at the same dose and continue to escalate as per protocol.

Dose re-escalation

Re-escalation back to the previous dose level is permitted in the absence of grade ≥3 haematologic or grade ≥2 non-haematologic treatment-related toxicity in the previous 4 weeks of treatment.

Axitinib dose interruption for surgery or surgical procedures

If major surgery or an interventional procedure (e.g. endoscopy) which is not part of NAXIVA study is required, treatment with axitinib must be interrupted a minimum of 36 hours and a maximum of 7 days before the procedure and the patient’s blood pressure should be monitored closely for hypotension. Patients may resume axitinib seven days after minor surgery and 2-3 weeks after major surgery, assuming the wounds have completely healed. The decision to resume axitinib therapy after surgery should be based on clinical judgment of adequate wound healing.

4.3. Duration of Treatment

Treatment with study drug should be discontinued if it is considered to be in the best interest of the patient. Reasons for treatment discontinuation include:

- Disease progression
- Occurrence of intolerable side effects
- Pregnancy
- Patient withdrawal of consent or non-compliance

4.4. Concomitant Therapy

Axitinib is metabolised primarily by liver enzymes, in particular CYP3A4. All medication considered necessary for the patients’ welfare and which is not expected to interfere with the evaluation of the study drug may be given at the discretion of the investigator. Concomitant medications must be recorded in the patients’ source documentation, as well as the appropriate pages of the CRF.

Contraindicated concurrent medications include:

Agents known to induce CYP3A4 or CYP1A2 including but not limited to:	Agents known to inhibit CYP1A2, CYP2C19 or CYP3A4 including but not limited to:

○ Amobarbital,<br>○ Carbamazepine,<br>○ Dexamethasone,<br>○ Felbamate,<br>○ Nevirapine,<br>○ Omeprazole,<br>○ Phenobarbital,<br>○ Phenytoin,<br>○ Primidone,<br>○ Rifabutin,<br>○ Rifampicin,<br>○ St John's wort	○ Amitriptyline<br>○ Amprenavir,<br>○ Artemisinin,<br>○ Atazanavir,<br>○ Atazanavir,<br>○ Cimetidine,<br>○ Ciprofloxacin,<br>○ Clarithromycin,<br>○ Delavirdine<br>○ Enoxacin,<br>○ Erythromycin,<br>○ Ethinyl,<br>○ Fluvoxamine,<br>○ Fosamprenavir<br>○ Grapefruit juice,<br>○ Imipramine<br>○ Indinavir,<br>○ Itraconazole,<br>○ Ketoconazole,<br>○ Lopinavir,<br>○ Mexiletine,<br>○ Miconazole,<br>○ Nelfinavir,<br>○ Ritonavir,<br>○ Saquinavir,<br>○ Tacrine,<br>○ Telithromycin,<br>○ Thiabendazole,<br>○ Ticlopidine.<br>○ Verapamil,<br>○ Zileuton

- Other approved or investigational systemic anticancer treatments, including chemotherapy, hormone therapy and immunotherapy.

- Other investigational drugs.

The use of potent antacids such as proton pump inhibitors (except those listed above) and histamine H2 antagonists is permissible, if medically necessary. However, patients requiring chronic antacid therapy should avoid their use for 2 hours before and for 2 hours after taking axitinib tablets.

The contraindicated medications detailed above may only be used in exceptional circumstances and following written confirmation from SCTRU of the CI's approval.

The current version of the SmPC should be used to assess whether any other concomitant medications are permissible.

Other supportive medicines such as thyroid replacement therapy, anti-emetics and anti-diarrhoeal medicines are permitted in accordance with local practice.

4.5. Compliance with Treatment

The study drug must not be used outside the context of the NAXIVA protocol.

Patients must be asked to bring all their trial medication every time they attend the clinic for the purposes of treatment compliance assessment and drug accountability. Every effort should be made to encourage patients to return the unused medication and empty packs. The unused tablets should be collected by the investigator/study nurse and counted to ascertain patient compliance, medication will then be returned to pharmacy for drug accountability prior to destruction according to local practices. Drug accountability and destruction records should be maintained by the local pharmacy.

4.6. Drug Supplies and Labelling

Drug Supply
Axitinib is supplied free of charge by Pfizer to Tayside Pharmaceuticals as 1mg and 5mg immediate release film-coated tablets and supplied in blister packs. Each 1mg and 5mg carton will contain 56 tablets (four blister sheets of 14 tablets per pack).

Tayside Pharmaceuticals will repack 2 intact blister sheets of 14 tablets each into a new carton along with the patient information leaflet (PIL) for each strength of axitinib. Each carton will then be labelled as per approved label. Release of the re-packaged drug will be carried out by a Qualified Person (QP) at Tayside Pharmaceuticals. IMP will then be dispatched to sites. The schedule of responsibilities for this arrangement is detailed in the contract between Tayside Pharmaceuticals and the sponsor.

SCTRU will arrange for a supply of axitinib to be sent from Tayside Pharmaceuticals to the relevant pharmacy department following centre initiation. Local R&D approval and a study agreement must be in place before any drug can be shipped to sites.

Sites will receive an initial supply of axitinib after site activation. Each site will receive; 2x 5mg pack as a starting dose and 3x 1mg packs of axitinib to cover any unexpected dose adjustments within the first 2 weeks of the patient schedule.
SCTRU will closely monitor axitinib supply at sites however sites will be expected to notify SCTRU when stock is required as per the NAXIVA Pharmacy Manual. A trial specific order form will be included in the pharmacy pack. Sites must have a minimum of 1x 5mg pack and 3x 1mg packs of axitinib in stock during the recruitment period.

Labelling
All study drug supplied by Pfizer will be re-packaged and labelled according to Annex 13 of the 'Good Manufacturing Practice'. The drugs will be re-packaged, labelled, QP released and distributed by Tayside Pharmaceuticals to sites.

4.7. Drug Storage Accountability

Storage
Axitinib should be stored in the original package in order to protect from moisture and should be stored at controlled room temperature (between 15°C and 30°C). The local pharmacy is responsible for ensuring that the study medication is stored in an appropriate secured area. Study treatment must be kept out of the reach and sight of children.

Accountability
The investigator or a delegated individual (e.g. pharmacist) must ensure that the study drug is stored and dispensed in accordance with hospital standard operating procedures and applicable regulatory requirements.

The medication provided for this study is for clinical trial use only as directed. Drug distribution and accountability logs will be provided to the site in a pharmacy pack. It is the

investigator's responsibility to establish a system for handling the investigational product to ensure that:

- Deliveries of investigational products from Pfizer via Tayside Pharmaceuticals are correctly received by a responsible person (e.g. pharmacist or suitable pharmacy designee) and are handled and stored correctly and safely.
- Investigational products are dispensed only to study participants, and in accordance with the protocol.
- Participants return any unused investigational product and all empty packs to the investigator.
- A dispensing record (which will include the identification of the participant to whom the investigational product was dispensed, the date of dispensing, the quantity of investigational product dispensed, and the date and quantity of any unused investigational product returned to the pharmacy) is accurately maintained. Any discrepancies must be accounted for on the appropriate form.

In the case that any study drug is damaged, please contact SCTRU for reconciliation and replacement.

At the termination of the study or at the request of the sponsor, all unused drugs will be accounted for and destroyed locally at the study sites. Certificates of delivery and destruction or return must be signed and copies retained in the Investigator Site File.

Accountability records must be completed and any study drug remaining at the end of the trial must be destroyed according to the sites local standard procedures.

5. ASSESSMENT OF EFFICACY

5.1. Treatment and Examination Schedule

Activity	Screening (Up to 4 weeks prior to enrolment)	Pre-treatment Day 1, Week 1	Week 3	Week 5	Week 7	Week 9	Week 9 (min 36 hours after stopping axitinib)	6 week post surgery follow up	12 week post surgery follow up
Confirmation of histological diagnosis of ccRCC via image guided biopsy (5 cores: 1 for diagnosis and 4 cores for research as per the NAXIVA laboratory manual)	X								
Medical History	X								
Physical examination including ECOG & Karnofsky performance status, body weight, height, temperature, blood pressure[2], heart rate, respiratory rate.	X	X[1]	X	X	X	X		X	X
Haematology, coagulation, glucose, renal and liver function, serum calcium, CRP and LDH.	X	X[1]	X	X	X	X		X	X
Urinalysis for protein	X	X[1]	X	X	X	X			
Serum Thyroid Function Tests (Free T4 and TSH)	X	X[1]	X	X	X				
Pregnancy Test (serum or urine) if applicable	X								
CT Chest, Abdomen and Pelvis for IVC VTT and primary tumour assessment using RECIST 1.1 and Mayo classification[3]	X (all)					X (M1 only)			X (all)
CT Chest to check for development of metastasis			X (M0 only)			X (M0 only)			
Contrast enhanced MRI abdomen (as per the NAXIVA MRI SOP)	X		X			X			
Blood samples and urine sample for biomarker analysis		X	X	X	X	X			X
Assessment of compliance with study medication			X	X	X	X			
Dispense axitinib		X	X	X	X				
Dose Escalation (5 to 7 to10mg BID) (depending on toxicities and blood pressure) [4]			X	X	X				
Stop axitinib (minimum 36 hours, maximum 7 days prior to surgery)						X			
Assessment of concomitant medications		X	X	X	X				
Assessment of symptoms (CTCAE V4)	X	X	X	X	X	X		X	X
Nephrectomy and IVC tumour thrombectomy							X		

[1] Laboratory and clinical assessments day 1 week 1 may be omitted if conducted within previous 7 days as part of screening assessment (apart from blood and urine for biomarker analysis).
[2] Blood pressure readings are required at each time point and should be taken by a healthcare professional using an appropriate calibrated machine.
[3] Where the scan has taken place prior to consent but within 28 days prior to enrolment the patient does not require to be re-scanned. See section 5.2.1
[4] Aggressive increasing of axitinib dose at each to ensure effective dose reached in window period.

5.2. Schedule of Assessments

5.2.1. Screening Procedures

Screening procedures within 4 weeks prior to study enrolment.

- Clinical assessment
    - Signed informed consent
    - Medical history
    - ECOG & Karnofsky performance status (appendix 1)
    - Body weight
    - Height
    - Blood pressure
    - Heart rate
    - Respiratory rate
    - Temperature
    - Symptom assessment (CTCAE V4)

- Histological assessment
    - Confirmation of diagnosis of ccRCC via image guided biopsy (5 cores should be taken for diagnosis/research as per the 'NAXIVA laboratory manual')

- Laboratory determinations
    - FBC, coagulation, glucose, renal and liver function, serum calcium, C - reactive protein (CRP) and Lactate Dehydrogenase (LDH).
    - Urinalysis
    - Serum thyroid function tests (Free T4 and Thyroid stimulating hormone (TSH)).
    - Pregnancy Test (serum or urine) if applicable (must be performed a maximum of 14 days prior to receiving 1st dose of axitinib)

- Radiological assessment
    - CT thorax, abdomen and pelvis for IVC VTT and primary tumour assessment using RECIST 1.1 criteria and the Mayo classification
    - Diffusion/ contrast enhanced MRI abdomen

Mayo Classification assessment will be conducted using MRI abdomen (preferentially, or CT abdomen if necessary) at screening and week 9 (prior to surgery). All MRI scans should be carried out as per the NAXIVA MRI SOP.

As per standard of care, patients may have a CT chest, abdomen and pelvis scan prior to consent. Where the scan has taken place within 28 days prior to enrolment this can be accepted and the patient does not require to be rescanned. If the CT scan is carried out >28 days prior to the planned enrolment date, this must be discussed with the Chief Investigator with prior to enrolment.

5.2.2. Study Enrolment

The participant's research nurse and/or doctor will screen the participant to ensure that they meet the trial eligibility criteria, after obtaining participant consent for any additional trial procedures.

Patients will be enrolled centrally with the Scottish Clinical Trials Research Unit (SCTRU). An eligibility and enrolment checklist must be completed prior to enrolment. Enrolment should take place within 72 hours prior to the planned start date of axitinib.

Once consent has been obtained and eligibility confirmed the participant should be enrolled by emailing the NAXIVA team at the Scottish Clinical Trials Research Unit:
**SCTRU enrolment email: NSS.SCTRU@nhs.net**

Referring to the NAXIVA site instructions, the enrolment CRF is required to be sent with the following information complete (as a minimum);

- Name of hospital.
- Confirmation that the patient has given written informed consent for trial participation and the provision of biological samples.
- Confirmation that the patient is eligible for the trial by completion of the eligibility checklist.
- Patients initials, date of birth, sex, NHS/CHI number and date of proven clear cell RCC (metastatic/ non-metastatic).
- Proposed start date of axitinib (must be within 72 hours of enrolment).

The site will inform the participants General Practitioner (GP) of the participants enrolment, if the participant gives consent to do so.

It may be possible for participants to be recruited into other clinical trials, but this should be discussed with the CI via SCTRU before this is considered.

5.2.3. Pre-treatment Assessment day 1, week 1 (within 7 days)

- Laboratory determinations*
  - FBC, coagulation, glucose, renal and liver function, serum calcium, CRP and LDH
  - Urinalysis
  - Serum thyroid function tests (Free T4 and TSH)
  - Blood and urine samples for biomarker analysis (NB. patients should be asked to fast for 6 hours prior to urine analysis, and to not take axitinib until after their bloods have been sampled for the biomarker analyses)

- Clinical assessment*
  - ECOG & Karnofsky performance status (appendix 1)
  - Body weight
  - Height
  - Blood pressure
  - Heart rate
  - Respiratory rate
  - Temperature
  - Symptom assessment (CTCAE V4)
  - Assessment for concomitant medications.

* Laboratory and clinical assessments day 1 week 1 may be omitted if conducted within previous 7 days as part of screening assessment (apart from blood and urine for biomarker analysis).

- Radiological assessment
  - CT thorax, abdomen and pelvis for IVC VTT (all patients) and primary tumour assessment using RECIST 1.1 criteria and the Mayo classification (is not required if CT carried out at screening/diagnosis)
    Imaging to be acquired following intravenous contrast medium assuming no contraindications. Thin slice (at least 2mm) imaging will be acquired as an arterial phase in the throax and as a portal venous phase of the abdomen and pelvis.

  - Diffusion/ contrast enhanced MRI abdomen (all patients), (is not required if contrast enhanced MRI carried out at screening/diagnosis)

- Pharmacy
  - Dispense axitinib

5.2.4. Assessment at day 1, week 3 (within 7 days excluding scans)

- Laboratory determinations (+/- 7 days)
  - FBC, coagulation, glucose, renal and liver function, serum calcium, CRP and LDH
  - Urinalysis
  - Serum thyroid function tests (Free T4 and TSH)
  - Blood and urine sample for biomarker analysis (NB. patients should be asked to fast for 6 hours prior to urine analysis)

- Clinical assessment (+/- 7 days)
  - ECOG & Karnofsky performance status (appendix 1)
  - Body weight
  - Height
  - Blood pressure
  - Heart rate
  - Respiratory rate
  - Temperature
  - Symptom assessment (CTCAE V4)
  - Assessment for concomitant medications
  - Assessment of compliance with study medication
  - Dose evaluation and escalation (where appropriate)

- Radiological assessment (to be carried out between days 14-21 of the start of treatment)
  - CT chest for IVC VTT (M0 patients only) to check for development of metastasis
  - Diffusion/ contrast enhanced MRI abdomen (all patients)

- Pharmacy
  - Dispense axitinib (dose modification as per section 4.2 if applicable)

5.2.5. Assessment at day 1, week 5 (within 7 days)

- Laboratory determinations
  - FBC, coagulation, glucose, renal and liver function, serum calcium, CRP and LDH
  - Urinalysis
  - Serum thyroid function tests (Free T4 and TSH)
  - Blood, urine sample for biomarker analysis (NB. patients should be asked to fast for 6 hours prior to urine analysis)

- Clinical assessment
  - ECOG & Karnofsky performance status (appendix 1)
  - Body weight
  - Height
  - Blood pressure
  - Heart rate
  - Respiratory rate
  - Temperature
  - Symptom assessment (CTCAE V4)
  - Assessment for concomitant medications
  - Assessment of compliance with study medication
  - Dose evaluation and escalation (where appropriate)

- Pharmacy
  - Dispense Axitinib (dose modification as per section 4.2 if applicable)

5.2.6. Assessment at day 1, week 7 (within 7 days)

- Laboratory determinations
  - FBC, coagulation, glucose, renal and liver function, serum calcium, CRP and LDH
  - Urinalysis
  - Serum thyroid function tests (Free T4 and TSH)
  - Blood and urine sample for biomarker analysis (NB. patients should be asked to fast for 6 hours prior to urine analysis)

- Clinical assessment
  - ECOG & Karnofsky performance status (appendix 1)
  - Body weight
  - Height
  - Blood pressure
  - Heart rate
  - Respiratory rate
  - Temperature
  - Symptom assessment (CTCAE V4)
  - Assessment for concomitant medications
  - Assessment of compliance with study medication
  - Dose evaluation and escalation (where appropriate)

- Pharmacy
  - Dispense axitinib (dose modification as per section 4.2 if applicable)

5.2.7. Assessment at day 1, week 9 (within 7 days)

- Laboratory determinations
    - FBC, coagulation, glucose, renal and liver function, serum calcium, CRP and LDH
    - Urinalysis
    - Blood and urine sample for biomarker analysis (NB. patients should be asked to fast for 6 hours prior to urine analysis)

- Clinical assessment
    - ECOG & Karnofsky performance status
    - Body weight
    - Height
    - Blood pressure
    - Heart rate
    - Respiratory rate
    - Temperature
    - Symptom assessment (CTCAE V4)
    - Assessment of compliance with study medication

- Radiological assessment
    - CT thorax, abdomen and pelvis (M1 patients) for IVC VTT and primary tumour assessment using RECIST 1.1 criteria and the Mayo classification

    - CT chest (M0 patients) for IVC VTT to check for development of chest metastasis.

    - Diffusion/ contrast enhanced MRI abdomen (all patients)

Mayo Classification assessment will be conducted using MRI abdomen (preferentially, or CT abdomen if necessary) at screening and week 9 (prior to surgery). All MRI scans should be carried out as per the NAXIVA MRI SOP.

5.2.8. Surgery day 2-7, week 9

Surgery should be carried out a minimum of 36 hours and a maximum of 7 days after the patient's final axitinib dose.

- Surgery
    - Nephrectomy and IVC tumour thrombectomy

The surgeon should complete the surgery CRF on the day of surgery.

5.2.9. Follow up assessment- 6 weeks post surgery (within 7 days)

- Laboratory determinations
    - FBC, coagulation, glucose, renal and liver function, serum calcium, CRP and LDH

- Clinical assessment
    - ECOG & Karnofsky performance status (appendix 1)

  - Body weight
  - Height
  - Blood pressure
  - Heart rate
  - Respiratory rate
  - Temperature
  - Symptom assessment (CTCAE V4)

5.2.10. Follow up assessment- 12 weeks post surgery (within 7 days)

- Laboratory determinations
  - FBC, coagulation, glucose, renal and liver function, serum calcium, CRP and LDH
  - Blood and urine sample for biomarker analysis (NB. patients should be asked to fast for 6 hours prior to urine analysis)

- Clinical assessment
  - ECOG & Karnofsky performance status (appendix 1)
  - Body weight
  - Height
  - Blood pressure
  - Heart rate
  - Respiratory rate
  - Temperature
  - Symptom assessment (CTCAE V4)

- Radiological assessment
  - CT thorax, abdomen and pelvis (all patients) for restaging.

5.2.11. Imaging

- All imaging data will be collated centrally.
- CT and RECIST assessment will be undertaken locally.
- MRI assessment will be undertaken at a single site to ensure consistency.
- All MRI scans must be carried out as per the NAXIVA MRI SOP

<u>Radiological endpoints</u>

Primary
- The VTT position in relation to the IVC for the Mayo classification will be performed using MRI abdomen (preferably) or CT if necessary.

Secondary
- RECIST 1.1 criteria will be used for CT evaluation

Exploratory
- The following exploratory endpoints may also be evaluated on MRI
  - VTT volume will be calculated using the thin slice MRI images reconstructed where appropriate. A small group of readers will undertake this and where possible automated segmentation will be used for consistency.
  - Change in the maximum VTT length using coronal MRI reconstructions

- Apparent Diffusion Coefficient (DWI) changes over time of both the whole tumour and VTT.
- Textural features of the whole tumour and VTT on MRI as a measure of heterogeneity, as well as changes in heterogeneity during treatment. This will be measured using semi-automated proprietary software where possible.

5.3. Withdrawal of Subjects

Patients *discontinue* from the study for reasons such as safety, non-compliance or withdrawal of consent, etc.

Discounted patients will be followed up for efficacy and safety. Nephrectomy, follow up (week 6 and week 12) data and bio-marker translational samples should be captured as per the site instructions (unless patients have withdrawn consent, deceased or are lost to follow up.

All patients who are permanently removed from treatment for intolerable toxicity should receive a post treatment CT Chest, Abdomen and Pelvis scan within 2 weeks of a decision to stop treatment. The CT scan should be assessed according to RECIST 1.1 criteria, and the patient should revert to standard of care.

Patients may *withdraw* from the study at any point; no further data collection will take place, and will be censored at the point of withdrawal this should be indicated on the case report form (CRF) as per completion guidelines.

Patients who consent to the trial but do not start trial treatment will be withdrawn from the trial. These patients will not be followed up as part of the study and will be expedited to surgery as per standard practice.
For any patient that did not start study treatment, a further patient would be enrolled to the trial to maintain the target number of patients for analysis.

6. Translational Research

Tissue, blood and urine will be collected at baseline (all sample types), throughout the 8 week treatment period (blood and urine) and at surgery (all sample types). Sites will be expected to collect all tissue, blood and urine samples as per the NAXIVA laboratory manual. The analysis streams will be as follows, with the aim of identifying molecular correlates to the clinical outcomes of this study.

6.1. Circulating tumour DNA (ctDNA)

In view intratumoural heterogeneity, the use of tumour tissue for the development of prognostic and predictive biomarkers in ccRCC is challenging (20). Circulating tumour DNA (ctDNA) has been established to represent the tumour biology and may be superior to tissue as heterogeneity will be negated (21). Obtaining ctDNA from blood from patients with VTT is the ideal platform on which to validate ctDNA in RCC because of the high likelihood of DNA shedding from the tumour directly into the circulation. Furthermore, the change in ctDNA profiles from baseline to treatment phase to post-treatment phase may be informative regarding markers of tumour response.

6.2. Metabolomics

It is well established that there is a strong metabolic basis to ccRCC. The presence of the Warburg effect has revealed several novel targets for treatment or tumour surveillance (22).

Metabolites will be assayed in urine, blood and tissue across the various time points of the NAXIVA study to assess the use of these molecules in patients with VTT and with axitinib therapy.

6.3. Predictive biomarker studies

There has been previous identification of a prognostic biomarker signature for response to sunitinib. The same biomarker profile will be validated for axitinib using the matched biopsy and nephrectomy tumour tissue samples obtained in this study.

6.4. Comparative study of IVC VTT in untreated and treated patients

Dovetailing in with ongoing studies looking at the invasive front of the IVC VTT in TKI naïve patients, the VTT samples from NAXIVA will be assessed using the same biomarker pipeline specifically searching for evidence in alterations in EMT or invasion profiles. The hypothesis being that treatment with axitinib abrogates some of the invasive features of the VTT.

6.5. Imaging

There is little published data on the best imaging method for assessing the IVC VTT. USS, CT and MRI are all currently used in different centres. Surgical outcome studies have shown that patients with solid VTT will have a superior oncological outcome compared to friable VTT, yet there are no radiological data on how to make this assessment. A non-invasive imaging biomarker of VTT composition would help to guide surgery in patients with borderline fitness. Textural analysis of the serial CT and MRI scans undertaken as part of NAXIVA will be performed and compared to the surgical outcome to assess if VTT composition and the effect of drug on VTT can be determined on imaging. Renal vein and IVC VTT volumes will be measured using MR venography techniques developed in Cambridge and % changes in post-contrast enhancement within the thrombus will be assessed from dynamic contrast-enhanced MRI (DCE-MRI) data. Exploratory analyses will include: changes in total tumour volume in the renal vein and IVC (CT and MRI); changes in the degree of enhancement after intravenous contrast administration (MRI); changes in imaging heterogeneity (texture) of the renal and IVC VTT (CT and MRI); differentiation of solid from friable IVC VTT using MR venography and physiological manoeuvres (Valsalva).

6.6. Immune Analysis

It is known that RCC is a typical immunogenic tumour with immune mechanisms known to play an important role in its natural history. Recent studies have also shown that TKI treatment can influence the immune system and, importantly, changes in the immune response may affect response and survival. The immune aim to identify immune- and cytokine-based signatures in PBMCs, plasma and urine which predict response to axitinib treatment in RCC patients. These studies will also provide important information about the effect of axitinib on the immune system in RCC, and vice versa, including providing comprehensive analysis of how peripheral immune cell populations and cytokines vary during treatment with axitinib, and whether they correlate with other markers of response and resistance in RCC patients. Importantly, it will also provide essential immunological information to inform biologically driven, rational, scheduling of future studies examining combinations of axitinib and immunotherapy.

7. PHARMACOVIGILANCE

7.1. Definitions

Adverse Event (AE): An adverse event (AE) is any untoward medical occurrence in a patient which does not necessarily have a causal relationship with the study treatments or procedures. An adverse event can therefore be any unfavourable and unintended sign (including an abnormal laboratory finding), symptom, or disease temporally associated with a treatment or procedure, whether or not considered related.

Adverse Reaction (AR): All noxious and unintended responses related to a study treatment or procedure should be considered adverse drug reactions.

Serious Adverse Event (SAE): Any untoward medical occurrence in a patient that

a) Results in death
b) Is life-threatening
c) Requires hospitalisation or prolongation of existing hospitalisation
d) Results in persistent or significant disability or incapacity
e) Consists of a congenital anomaly or birth defect
f) Is otherwise considered to be medically significant by the investigator

The term “life-threatening” refers to an event in which the patient was at risk of death at the time of the event; it does not refer to an event which hypothetically might have caused death if it were more severe.

Hospitalisations planned prior to enrolment in the trial or for social reasons should not normally be considered as SAEs unless the hospitalisation has to be prolonged. Treatment in an emergency room of less than 24 hours or on an out-patient basis that does not meet any other serious criteria should not be considered as an SAE.

Hospitalisation for nephrectomy is not considered a SAE where this goes ahead as planned within the trial. Prolonged hospitalisation, however, should be reported as SAE.

Suspected Unexpected Serious Adverse Reaction (SUSAR): A suspected unexpected serious adverse reaction (SUSAR) is an adverse reaction that is classified as serious and it is suspected that it is caused by a study treatment or procedure. The nature, severity or outcome of this adverse reaction also must not be consistent with the current version of the SmPC for the treatment or procedure.

7.2. Recording of Adverse Events

All related Adverse Reactions will be recorded in the Case Report Form. Any Adverse Reaction considered unrelated will not be recorded.
All adverse reactions that occur after the signing of written informed consent and within 30 days after the final study treatment will be recorded on the post 6 week surgery follow up CRF. The patient is not required to be seen at the 30 day post axitinib time point. The exception to this would be any event occurring after signing the informed consent and prior to commencing study treatment that is considered unrelated to trial procedures. In addition any events occurring more than 30 days after final study drug treatment that are deemed to be related to the study drug should be notified to SCTRU as detailed in section 7.3.

Any medical conditions or diseases present prior to signing of informed consent should only be considered an adverse event if there is a worsening of the condition.

Please refer to Appendix 2 for details on the Causality.

7.3. Recording and Reporting of Serious Adverse Events

Contact Details for Reporting SAEs	
**SCTRU Fax:**	+44 131 275 7512 (preferred method)
**SCTRU Telephone:**	+44 131 275 7276 (Mon – Fri 9am-4pm)
**Or:**	+44 131 316 4278 (Mon – Fri 9am-4pm)

All serious adverse events that occur after the signing of written informed consent and within 30 days after the final study treatment will be recorded on the SAE report form. The exception to this would be any event occurring after signing the informed consent and prior to commencing study treatment that is considered unrelated to trial procedures. In addition any events occurring more than 30 days after final study treatment that are deemed to be related to the study drug should be notified to SCTRU as above.

The SAE report form must be signed by the Principal Investigator (PI) of the centre involved and faxed to SCTRU within 24 hours of first becoming aware of the event. All initial SAE reports should contain the following minimum information:

- Reporter information
- At least one subject identifier (trial number/patient initials)
- Event term
- Assessment of relatedness
- Serious criteria

A fax or email receipt will be sent to the relevant centre by SCTRU to acknowledge receipt of the SAE report form, and SCTRU will notify the CI.

All SAEs will be forwarded to the CI by SCTRU for assessment of expectedness against the current SmPC. Any SAE that is deemed to be both related and unexpected (i.e. a SUSAR) will be notified to the appropriate Competent Authorities and Research Ethics Committees within 7 days of becoming aware of the event for fatal or life threatening events and 15 days for all other serious events.
SUSARs should be reported to the Research Ethics Committee (REC) accompanied by the ‘safety reports to the REC covering form’. The coordinator of the REC should acknowledge receipt of the safety report within 30 days by signing and returning a copy of the covering form.
SCTRU will then notify the CI and the PI’s at all of the participating centres of the occurrence of all SUSARs.

Hospitalisations planned prior to enrolment in the trial or for social reasons should not normally be considered as SAEs unless the hospitalisation has to be prolonged. Treatment in an emergency room of less than 24 hours or on an out-patient basis that does not meet any other serious criteria should not be considered as an SAE.

7.4. Developmental Safety Update Report

An Annual Safety Report will be submitted to the appropriate Competent Authorities and Ethics Committees, once a year for the duration of the trial. The time frame for the report starts with the date of first authorisation by a competent authority in an EU member state and the report should be submitted within 60 days of the anniversary of first authorisation.

7.5. Pregnancies

Any pregnancy in a trial participant or their partner that occurs during study participation should be reported to SCTRU within 24 hours of becoming aware of its occurrence, using the contact details in Section 7.3. The pregnancy should be followed up to determine outcome, including spontaneous or voluntary abortion, details of birth and presence or absence of any birth defects, congenital abnormalities or maternal or newborn complications. Any birth defects or congenital abnormalities must be reported as SAEs.

8. DATA MANAGEMENT

All data will be handled, computerised and stored in accordance with the General Data Protection Regulations (GDPR) and NHS National Services Scotland Confidentiality Guidelines.

8.1. Data Collection

Data generated will be collected by SCTRU. SCTRU will be responsible for checking the data, and validating it. The data collected will include:

- initial clinical details at registration
- drug administration (CTIMPs)
- concomitant medications
- adverse events
- survival/ recurrence details
- surgical techniques
- tumour measurements
- vena cava thrombus measurement
- Dose escalation details

8.2. Record Keeping and Archiving

SCTRU will store study documentation until the end of patient follow up. The documentation will then be archived according to current legislative requirements.

9. STATISTICS

9.1. Sample Size

The aim is to recruit 20 patients over a 24 month period. A Simon two stage minimax design to distinguish a <5% from a >25% improvement in the Mayo classification requires 20 patients (90% power, 10% 1-sided). Thirteen patients would be recruited at first stage; if at least 1 patient had an improvement in the Mayo classification a further 7 patients would be recruited (to the final total of 20 patients).

9.2. Analysis Plan

The main analysis will be conducted on the "as-treated" population. All patients who receive study drug will be included in the interim and final analysis. Any patient who withdraws from the study prior to receiving drug will not be included in the analysis. At least 3 patients showing a reduction in Mayo Classification would produce a positive trial.

Interim Analysis
An interim analysis will be performed after thirteen patients have been recruited. If no patients have show an improvement in their Mayo classification the trial will be stopped for futility. If at any point in the study 3 patients have a progression of their thrombus (increase in Mayo classification) but remain surgically resectable the trial will be stopped. (Probability of seeing 3 or more progressions if true progression rate <5% is <7.5%). There is no formal stopping rule for efficacy.

The primary analysis will be made on the "treated" population, with patients being included in the analysis only if they have received at least one dose of the drug. In order to assess the impact of any biases an analysis of the "intention to treat" population will also be carried out. This will include all patients who are registered as eligible for the trial, regardless of whether or not the subsequently received study medication.

Final Analysis
The final analysis will be performed once the end of study has been declared and will include summaries of the % of evaluable patient with improvement in the Mayo classification; % change in surgical approach; the response rates as determined by RECIST criteria V1.1 and an evaluation of morbidity utilising the Clavien-Dindo classification system.

The dose of axitinib delivered will be summarised with the median and range of total dose being presented. Adverse events will be classified using CTCAE V4 and the worst grade for each toxicity will be summarised.

9.3. End of Study

This study will end when the below criteria are deemed complete by the sponsor; Last patient last visit (12 week post surgery follow up) complete and the database is clean and frozen for analysis.

10. ACCESS TO SOURCE DATA/ DOCUMENTS

The investigator, by accepting to participate to this protocol, agrees to co-operate fully with any quality assurance visit undertaken by third parties, including representatives from the Sponsor, SCTRU, national and/or foreign regulatory authorities or company supplying the product under investigation, as well as to allow direct access to documentation pertaining to the clinical trial (including CRFs, source documents, hospital patient charts and other study files) to these authorised individuals.

11. QUALITY CONTROL AND QUALITY ASSURANCE

Quality control will be maintained through adherence to appendix 7, Good Clinical Practice (GCP) and the coordinating centre's SOPs. The coordinating centre will monitor receipt of

CRFs and evaluate incoming CRFs for compliance with the protocol, inconsistencies and missing data.

11.1. Monitoring Visits

We have allowed for site visits in the UK to enable monitoring by SCTRU to check patient consent forms, confirm compliance with the protocol and complete source data verification (SDV) on the patient data as defined in the Data Monitoring Plan. Higher levels of monitoring will be performed, if requested, by the Data Monitoring Committee, or if the investigators, the Trial Management group or Trial Steering Committee identify particular safety issues.

11.2. Data Monitoring and Ethics Committee

An independent Data Monitoring Committee (DMC) will be established and will meet 6 monthly in the first instance and annually thereafter (and at any other time at the committee's discretion). There will be an extra meeting of the committee after 13 patients have been recruited. None of the committee members will be involved in the trial. The committee will receive regular reports from SCTRU. It will submit its comments and recommendations to the Trial Steering Committee and Trial Management Group (TMG).

11.3. Trial Steering Committee

A trial steering committee will be established to provide overall supervision of the trial, in particular; trial progress, adherence to protocol, patient safety, and consideration of new information. The committee will meet 3 monthly in the first instance and then 6 monthly thereafter. The committee will then meet 3 monthly during close out/ final analysis.

12. ETHICAL CONSIDERATIONS

Ethical approval by the East of England – Cambridge Committee will be required before the trial can be started.
The trial will be carried out according to GCP guidelines as defined by paragraph 28 and Schedule 1 Part 2 of the Medicines for Human Use (Clinical Trials) Regulations, 2004, and the Clinical Trials Directive (2001/20/EC) elsewhere in the European Union and follow the principles of research governance.

The use and storage of human tissue will be carried out in accordance with The Human Tissue Act (2004) and The Human Tissue (Scotland) Act (2006). Human Tissues is defined as any material which has come from a human body that consists of, or includes human cells and includes blood and other bodily fluids.

12.1. Patient Confidentiality

The patient's full name, date of birth, hospital number and NHS number (Community Health Index and/or hospital number in Scotland) will be collected to enable tracing through national records. The personal data recorded on all records will be regarded as confidential, and to preserve each patient's anonymity, only their initials and date of birth will be recorded on CRFs. The patients will be identified within the CRFs by the use of a unique trial number allocated to them upon entry into the study.
The PI (or delegate) at each site must keep a log of patients' trial numbers, names, addresses and hospital numbers. The PI must ensure that patient confidentiality is maintained and that all trial documents (e.g. consent forms) are maintained in strict confidence.

SCTRU will maintain the confidentiality of all patient data and will not reproduce or disclose any information by which patients could be identified. Patients will only be referred to by Trial Number, Initials and Date of Birth in any essential trial related correspondence, including Case Report Forms and Serious Adverse Event Reports.

All patient identifiable data will be handled, computerised and stored in accordance with the GDPR and NHS National Services Scotland Confidentiality Guidelines.

12.2. Informed Consent

All patients will be informed of the aims of the study, the possible adverse events, the procedures and possible hazards to which they will be exposed, and the mechanism of treatment allocation. They will be informed as to the strict confidentiality of their patient data, but that their medical records may be reviewed for trial purposes by authorized individuals other than their treating physician. It will be emphasized that the participation is voluntary and that the patient is allowed to refuse further participation in the protocol whenever they want. This will not prejudice the patient's subsequent care.

Documented informed consent must be obtained for all patients included in the study before they are enrolled. This must be done in accordance with the national and local regulatory requirements and must conform to guidelines on Good Clinical Practice. That is, "the written informed consent form should be signed and personally dated by the patient or by the patient's legally acceptable representative".

Copies of the patient information sheets and consent forms are provided in appendices 4 & 5.

All Patient Information Sheets & Informed Consent Forms will be version controlled and dated and this information will always be stated in any communication with ethics committees.

13. RESEARCH GOVERNANCE

Sponsor (CSA) – The sponsor will have overall responsibility for the design, co-ordination and management of the study. These include:

- Trial authorisation including responsibility for the protocol and obtaining approvals
- Ensuring that the trial is conducted according to GCP guidelines (22,23)
- Assessment of SAEs and providing a prompt response as to whether the SAE is a SUSAR.

Clinical Trials Unit – The sponsor has delegated the responsibility for overall project management, data management and monitoring to Scottish Clinical Trials Research Unit, NHS National Services Scotland
Responsibilities include:

a. Assistance with completion of the IRAS form and REC communication
b. Production of trial specific documentation (i.e. CRFs)
c. Facilitating set up of trial centres
d. Data management
e. Monitoring

f. Pharmacovigilance – Reporting of SARs / SUSARs

Statistical Analysis – A Principal Information Analyst, based at SCTRU, Edinburgh will undertake the final analysis arising for this study.

Local Project Teams – These will consist of Surgeons and/or Oncologists (responsible for introducing the patient to the study and ensuring eligibility and consent), Research Nurse (responsible for patient recruitment, obtaining consent and co-ordination of all aspects of data collection), Pathologists (responsible for tissue sample analysis), Radiologists and Radiographers (responsible for completing MRI and CT scans to protocol). Centres are specifically responsible for conducting the trial in accordance with the protocol, Standard Operating Procedures (SOPs), the trial agreement and Good Clinical Practice.

Trial Steering Committee and Data Monitoring and Ethics Committee – The Common Services Agency (CSA) will act as study sponsor. Central study co-ordination, data collection, monitoring and organisation of the data for the statistical analyses will be undertaken by Scottish Clinical Trials Research Unit, NHS National Services Scotland, which has processes in place to ensure that the study will not open to recruitment until appropriate approvals and authorisations have been obtained from the independent research ethics committee, and NHS Research and Development departments (R&D). The Trial Steering Committee (TSC), including members of the research team and an radiologist, oncologist, statistician, and lay members, will be responsible for the progress and conduct of the study and convene annually. A Trial Management Group will meet quarterly. A Data Monitoring and Ethics Committee (DMC) will convene 6 monthly, to review all data including adverse events and review the stopping policy for the trial, if necessary. Specific issues that will be looked at include: tolerability of initial dose, dose reductions, toxicities (including interaction of the mild increased bleeding risk), imaging & review schedules.

Laboratories – Accredited local laboratories shall be used for all on-study tests. Samples from the translational research will be transferred to a central laboratory based at the University of Cambridge. The details of the sampling, storage and shipping protocols are covered within the NAXIVA laboratory manual in accordance with all prevailing regulatory requirements.

14. FINANCING AND INSURANCE

This study is funded by Pfizer Limited and endorsed by Cancer Research UK (CRUK). Indemnity for participating hospitals is provided by the usual NHS indemnity arrangements.

15. PUBLICATION POLICY

All presentations and publications relating to the trial must be authorized by the Trial Management Group. The main trial results will be published in the name of the trial in a peer-reviewed journal, on behalf of all collaborators. The manuscript will be prepared by Trial Management Group, representatives from SCTRU, NHS National Services Scotland and high accruing clinicians. The trials offices and all participating Centers and clinicians will be acknowledged in this publication. Any data that might detrimentally affect the progress of the trial will not be released prior to the end of the trial. No investigator may present or attempt to publish data concerning their patients, which is directly relevant to the questions posed in the trial, until the main results have been published.

REFERENCE LIST

1. Kirkali Z, Van Poppel H. A critical analysis of surgery for kidney cancer with vena cava invasion. *Eur. Urol.* 2007;**52**(3):658–662.

2. Parra J et al. Oncological outcomes in patients undergoing radical nephrectomy and vena cava thrombectomy for renal cell carcinoma with venous extension: a single-centre experience. *Eur. J. Surg. Oncol. J. Eur. Soc. Surg. Oncol. Br. Assoc. Surg. Oncol.* 2011;**37**(5):422–428.

3. Lambert EH et al. Prognostic risk stratification and clinical outcomes in patients undergoing surgical treatment for renal cell carcinoma with vascular tumor thrombus. *Urology* 2007;**69**(6):1054–1058.

4. Rini, B.I., et al., *Axitinib versus sorafenib as second-line therapy for metastatic renal-cell carcinoma (mRCC): Results of phase III AXIS trial*, in *American Society of Clinical Oncology*. 2011, Journal of Clinical Oncology: Chicago, USA. p. Abstr 4503.

5. Schiller, J.H., et al., *Efficacy and safety of axitinib in patients with advanced non-small-cell lung cancer: results from a phase II study.* J Clin Oncol, 2009. **27**(23): p. 3836-41.

6. Cohen, E.E., et al., *Axitinib is an active treatment for all histologic subtypes of advanced thyroid cancer: results from a phase II study.* J Clin Oncol, 2008. **26**(29): p. 4708-13.

7. Kindler, H.L., et al., *Axitinib plus gemcitabine versus placebo plus gemcitabine in patients with advanced pancreatic adenocarcinoma: a double-blind randomised phase 3 study.* Lancet Oncol, 2011. **12**(3): p. 256-62.

8. Fruehauf, J., et al., *Multicenter, phase II study of axitinib, a selective second-generation inhibitor of vascular endothelial growth factor receptors 1, 2, and 3, in patients with metastatic melanoma.* Clin Cancer Res, 2011. **17**(23): p. 7462-9.

9. Eisenhauer, E.A., et al., *New response evaluation criteria in solid tumours: revised RECIST guideline (version 1.1).* Eur J Cancer, 2009. **45**(2): p. 228-47.

10. Rugo, H.S., et al., *Phase I trial of the oral antiangiogenesis agent AG-013736 in patients with advanced solid tumors: pharmacokinetic and clinical results.* J Clin Oncol, 2005. **23**(24): p. 5474-83.

11. Mukohara, T., et al., *Effect of axitinib (AG-013736) on fatigue, thyroid-stimulating hormone, and biomarkers: a phase I study in Japanese patients.* Cancer Sci, 2010. **101**(4): p. 963-8.

12. Rixe, O., et al., *Axitinib treatment in patients with cytokine-refractory metastatic renal-cell cancer: a phase II study.* Lancet Oncol, 2007. **8**(11): p. 975-84.

13. Rini, B.I., et al., *Phase II Study of Axitinib in Sorafenib-Refractory Metastatic Renal Cell Carcinoma.* Journal of Clinical Oncology, 2009. **27**(27): p. 4462-4468.

14. NCICTCAE. *NCI Common Terminology Criteria for Adverse Events (CTCAE) v.4 data files* 2010; Available from: http://evs.nci.nih.gov/ftp1/CTCAE/About.html.

15. Cost NG et al. The impact of targeted molecular therapies on the level of renal cell carcinoma vena caval tumor thrombus. *Eur. Urol.* 2011;**59**(6):912–918.

16. Bigot P et al. Neoadjuvant targeted molecular therapies in patients undergoing nephrectomy and inferior vena cava thrombectomy: is it useful? *World J. Urol.* 2014;**32**(1):109–114.

17. Karam JA et al. Phase 2 Trial of Neoadjuvant Axitinib in Patients with Locally Advanced Nonmetastatic Clear Cell Renal Cell Carcinoma. *Eur. Urol.* 2014;**66**(5):874-880

18. Bex A, Clarke N. Targeted caval cytoreduction: solid foundations or shifting sands? *Eur. Urol.* 2011;**59**(6):919–920; discussion 921–922.

19. Blute, M.L., et al., The Mayo Clinic experience with surgical management, complications and outcome for patients with renal cell carcinoma and venous tumour thrombus. *BJU Int.* 2004;**94**(1):33-41

20. Stewart G et al. Sunitinib Treatment Exacerbates Intratumoral Heterogeneity in Metastatic Renal Cancer. *Clin Cancer Res.* 2015;**21**(18):4212-4223

21. Murtaza M et al. Non-invasive analysis of acquired resistance to cancer therapy by sequencing of plasma DNA. *Nature* 2013;**497**(7447):108–112.

22. Srinivasan R, Ricketts CJ, Sourbier C, Linehan WM. New Strategies in Renal Cell Carcinoma: Targeting the Genetic and Metabolic Basis of Disease. *Clin Cancer Res.* 2015;**21**(1):10–17.

23. Frezza C et al. Haem oxygenase is synthetically lethal with the tumour suppressor fumarate hydratase. *Nature* 2011;**477**(7363):225–228.

Appendix 1 – ECOG and Karnofsky Performance Scores

Karnofsky Status	Karnofsky Score	ECOG Grade	ECOG Status
Normal, no complaints.	100	0	Fully active, able to carry on all pre-disease performance without restriction.
Able to carry on normal activities. Minor signs or symptoms of disease.	90	1	Restricted in physically strenuous activity but ambulatory and able to carry out work of a light or sedentary nature, e.g., light house work, office work.
Normal activity with effort.	80	1	Restricted in physically strenuous activity but ambulatory and able to carry out work of a light or sedentary nature, e.g., light house work, office work.
Care for self. Unable to carry on normal activity or to do active work.	70	2	Ambulatory and capable of all self care but unable to carry out any work activities. Up and about more than 50% of waking hours.
Requires occasional assistance, but able to care for most of his needs.	60	2	Ambulatory and capable of all self care but unable to carry out any work activities. Up and about more than 50% of waking hours.
Requires considerable assistance and frequent medical care.	50	3	Capable of only limited self care, confined to bed or chair more than 50% of waking hours.
Disabled. Requires special care and assistance.	40	3	Capable of only limited self care, confined to bed or chair more than 50% of waking hours.
Severely disabled. Hospitalisation indicated though death nonimminent.	30	4	Completely disabled. Cannot carry on any self care. Totally confined to bed or chair.
Very sick. Hospitalisation necessary. Active supportive treatment necessary.	20	4	Completely disabled. Cannot carry on any self care. Totally confined to bed or chair.
Moribund.	10	4	Completely disabled. Cannot carry on any self care. Totally confined to bed or chair.
Dead.	0	5	Dead.

Appendix 2 – Causality

The assignment of causality should be made by the investigator responsible for the care of the patient, using the definitions in the table below.
If any doubt about the causality exists the investigator should inform the Trials Centre who will notify the Chief Investigator.
In the case of discrepant views on causality between the investigator and others, the case will be discussed by all parties. In the event that no agreement is made, the MHRA will be informed of both points of view.

Description of causality of adverse events

Possibly Related: No	Unrelated	There is no evidence of any causal relationship.
	Unlikely	There is little evidence to suggest there is a causal relationship (e.g. the event did not occur within a reasonable time after administration of the trial medication). There is another reasonable explanation for the event (e.g. the patient's clinical condition, other concomitant treatment).
Possibly Related: Yes	Possible	There is some evidence to suggest a causal relationship (e.g. because the event occurs within a reasonable time after administration of the trial medication). However, the influence of other factors may have contributed to the event (e.g. the patient's clinical condition, other concomitant treatments).
	Probable	There is evidence to suggest a causal relationship and the influence of other factors is unlikely.
	Definitely	There is clear evidence to suggest a causal relationship and other possible contributing factors can be ruled out.

Appendix 3a – Investigator Statement (SCTRU Copy)

NAXIVA

PHASE II NEOADJUVANT STUDY OF AXITINIB FOR REDUCING EXTENT OF VENOUS TUMOUR THROMBUS IN CLEAR CELL RENAL CELL CANCER WITH VENOUS INVASION (NAXIVA)

Principal Investigator Declaration

I acknowledge receipt of version 2.0 dated 6th June 2018 of the NAXIVA trial protocol (REC approved DD/MM/YYYY) and I agree to perform this trial in accordance with this version of the protocol and Good Clinical Practice.

I understand that the safety of the patient is my first concern

Print Name: --

Hospital: --

Signed: --

Date: --

Please return this copy to: NAXIVA Trial Coordinator
Scottish Clinical Trials Research Unit,
Gyle Square,
1 South Gyle Crescent,
Edinburgh,
EH12 9EB

Appendix 3b – Investigator Statement (Investigator Copy)

NAXIVA

PHASE II NEOADJUVANT STUDY OF AXITINIB FOR REDUCING EXTENT OF VENOUS TUMOUR THROMBUS IN CLEAR CELL RENAL CELL CANCER WITH VENOUS INVASION (NAXIVA)

Principal Investigator Declaration

I acknowledge receipt of version 2.0 dated 6th June 2018 of the NAXIVA trial protocol (REC approved DD/MM/YYYY) I agree to perform this trial in accordance with this version of the protocol and Good Clinical Practice.

I understand that the safety of the patient is my first concern

Print Name: ---

Hospital: ---

Signed: ---

Date: ---

Please retain this copy and file in Investigator Site File.

Appendix 4- Associated Documents

1. Main Trial Patient Information Sheet and Consent
2. Translational studies Patient Information Sheet and Consent
3. Pharmacy Manual
4. Laboratory (Translational sampling) Manual
5. MRI SOP
6. CRF Booklet
7. Site Instructions
8. Pharmacovigilance Instructions

Documents can be requested from the NAXIVA Trial Team NSS.NAXIVA@nhs.net

Appendix 5– The Principles of ICH Good Clinical Practice

1. Clinical trials should be conducted in accordance with the ethical principles that have their origin in the Declaration of Helsinki, and that are consistent with GCP and the applicable regulatory requirement(s).
2. Before a trial is initiated, foreseeable risks and inconveniences should be weighed against the anticipated benefit for the individual trial subject and society. A trial should be initiated and continued only if the anticipated benefits justify the risks.
3. The rights, safety, and well-being of the trial subjects are the most important considerations and should prevail over interests of science and society.
4. The available nonclinical and clinical information on an investigational product should be adequate to support the proposed clinical trial.
5. Clinical trials should be scientifically sound, and described in a clear, detailed protocol.
6. A trial should be conducted in compliance with the protocol that has received prior institutional review board (IRB)/independent ethics committee (IEC) approval/ favourable opinion.
7. The medical care given to, and medical decisions made on behalf of, subjects should always be the responsibility of a qualified physician or, when appropriate, of a qualified dentist.
8. Each individual involved in conducting a trial should be qualified by education, training, and experience to perform his or her respective task(s).
9. Freely given informed consent should be obtained from every subject prior to clinical trial participation.
10. All clinical trial information should be recorded, handled, and stored in a way that allows its accurate reporting, interpretation and verification.
11. The confidentiality of records that could identify subjects should be protected, respecting the privacy and confidentiality rules in accordance with the applicable regulatory requirement(s).
12. Investigational products should be manufactured, handled, and stored in accordance with applicable good manufacturing practice (GMP). They should be used in accordance with the approved protocol.
13. Systems with procedures that assure the quality of every aspect of the trial should be implemented.
