# Supplementary material for: A Phase II study of neoadjuvant axitinib for reducing the extent of venous tumour thrombus in clear cell renal cell cancer with venous invasion (NAXIVA)
Source: Br J Cancer. 2022 Jun 23;127(6):1051–60. doi: 10.1038/s41416-022-01883-7 (PMC9470559; doi:10.1038/s41416-022-01883-7)

NAXIVA

Phase II **N**eoadjuvant study of **AXI**tinib for reducing extent of venous tumour thrombus in clear cell renal cell cancer with **V**enous inv**A**sion.

**ISRCTN:**	ISRCTN96273644
**EudraCT Number:**	2017-000619-17
**REC reference:**	17/EE/0240

Laboratory Manual

Version 2.3, 4th March 2020

Approved by;

Dr Sarah Welsh (Translational Lead) Date: 4th March 2020

Mr Grant Stewart (Chief Investigator) Date: 4th March 2020

Tracy McEleney (Clinical Trial Service Manager) Date: 4th March 2020

TABLE OF CONTENTS

1 CONTACT DETAILS 4
2 INTRODUCTION 5
3 TRANSLATIONAL SAMPLE SCHEDULE 5
4 TISSUE ACQUISITION AND PROCESSING AT TRIAL CENTRES 6
4.1 Tissue processing consumables required 6
4.2 screening Primary tumour tissue sample 7
4.2.1 Prioritisation of screening primary tumour tissue samples 7
4.3 nephrectomy specimen 8
4.3.1 Multiregion sampling 8
4.3.2 frozen samples 9
4.3.3 FFPE samples 9
4.3.4 Fresh tumour, tumour venous thrombus and normal kidney sampling 9
4.4 Paraffin Embedded Tissue Sections 9
5 BLOOD SAMPLING 10
5.1 Timing of blood sampling 10
5.2 Blood collection schedule 10
5.2.1 Whole blood (WB) – all centres 11
5.2.2 Plasma (P) – all centres 11
5.2.3 Buffy coat (BC) - for collection ONLY by sites NOT processing samples for PBMCs (as this step removes the peripheral blood mononuclear cells; PBMCs) 11
5.2.4 Peripheral blood mononuclear cells (PBMCs): For collection by SELECTED SITES only. 12
5.3 Blood sample transfer to The University of Cambridge 13
5.3.1 CPDA BLOOD TUBE TRANSFER TO THE UNIVERSITY OF CAMBRIDGE 13
5.3.2 WHOLE BLOOD, PLASMA, BUFFY COAT AND PBMCS – TRANSFER TO THE UNIVERSITY OF CAMBRIDGE 13
6 URINE SAMPLES 14
6.1 Timing of urine sampling 14
6.2 Urine collection schedule 14
6.3 URINE sample processing protocols 14
6.3.1 Processing of urine samples for ctDNA analysis 14
6.4 Urine sample transfer to University of Cambridge 15
7 STORAGE OF SAMPLES 15
8 TRANSFER OF SAMPLES TO THE UNIVERSITY OF CAMBRIDGE 15
8.1 General instructions 15
8.2 Documents required 16

8.2.1 ‘Fresh’ samples 16
8.2.2 All other samples EXCEPT ‘FRESH’ samples 16
8.3 Shipment process 16
8.3.1 ‘Fresh’ samples for transfer within 24 hours of collection 18
8.3.2 Frozen samples for transfer on dry ice 19
8.3.3 FFPE blocks for transfer at room temperature 20
8.3.4 CPDA TUBES for transfer at room temperature (FOR PBMC PROCESSING AT UNIVERSITY OF CAMBRIDGE) 20
Appendix Index 22

1 CONTACT DETAILS

Chief Investigator	**Mr Grant Stewart**<br>University of Cambridge<br>Academic Urology Group,<br>Box 43, Addenbrooke's Hospital,<br>Cambridge Biomedical Campus,<br>Hill's Road,<br>Cambridge, CB2 0QQ<br>Email: grant.stewart@addenbrookes.nhs.uk
Translational Lead	**Dr Sarah Welsh**<br>University of Cambridge<br>Department of Oncology<br>Box 193, Addenbrooke's Hospital<br>Hills Road<br>Cambridge<br>CB2 0QQ<br>Email: sarah.welsh@addenbrookes.nhs.uk

Scottish Clinical Trials Research Unit

Principal Trial Manager	**Kathleen Riddle**<br>Gyle Square,<br>1 South Gyle Crescent,<br>Edinburgh, EH12 9EB<br>Tel: 0131 275 7074<br>Fax: 0131 275 7512<br>Email: NSS.NAXIVA@nhs.net
Senior Trial Coordinator	**Michelle Welsh**<br>Gyle Square,<br>1 South Gyle Crescent,<br>Edinburgh, EH12 9EB<br>Tel: 0131 275 6567<br>Fax: 0131 275 7512<br>Email: NSS.NAXIVA@nhs.net

Trial Sponsor

COMMON SERVICES AGENCY (CSA) a body corporate re-established under Section 10 of the National Health Service (Scotland) Act 1978 as amended and having its principal office at Gyle Square, 1 South Gyle Crescent, Edinburgh, EH12 9EB acting through the Scottish Clinical Trials Research Unit.

All questions relating to the NAXIVA study should be addressed to the NAXIVA Team within the Scottish Clinical Trial Research Unit (SCTRU);

NSS.NAXIVA@nhs.net

2 INTRODUCTION

A key aim of this study is to evaluate changes in multiple molecular factors in relation to tumour response.
The expression of these putative biomarkers of renal cancer response will be evaluated in tissue donated before and following treatment to define therapeutic response to treatment. In addition, novel techniques (including circulating tumour DNA (ctDNA), immunophenotyping, and metabolomics analyses) will be applied to samples to gain a better fundamental understanding of the effect of axitinib on metabolites, immune function, and proteomics and also to assess the potential for these and other markers (such as ctDNA) for predicting or tracking response and/or toxicity. Downstream analysis of tissue for such studies will be performed at the Stewart, Rosenfeld, and Frezza Labs at The University of Cambridge, UK.

This translational sampling manual contains all the details and protocols needed for initial processing and shipment of translational samples for NAXIVA. It is imperative that these protocols are followed to enable high quality samples for reproducible down-stream analysis. These protocols may be different than those used for other studies. If samples cannot be processed as required in this manual, for whatever reason, this MUST be discussed ahead of sample collection. Any deviations from these protocols must be recorded on the sample record.

3 TRANSLATIONAL SAMPLE SCHEDULE

Tissue, blood, and urine samples will be collected for translational studies according to the table below.

<u>Table 1- Translational sampling schedule</u>

	Within 28 days of enrolment	Day 1, Week 1	Day 1, Week 3	Day 1, Week 5	Day 1, Week 7	Day 1, Week 9	Nephrectomy	12 weeks post surgery follow up
**Screening primary renal tumour tissue** Percutaneous image guided biopsy of non-necrotic region of primary renal tumour (5 cores);<br>- 2 Formalin-fixation and paraffin embedded<br>- 1 Frozen and stored at -80°C<br>- 2 Fresh and placed in media for transfer to University of Cambridge within 24 hours	X							
**Nephrectomy specimen** Tumour multi-region sampling (minimum of 5 spatially separate tumour samples including venous invasion tissue with an accompanying map). Additionally (for transfer to University of Cambridge within 24 hours);<br>- 1 Fresh tumour sample placed in media<br>- 1 Fresh VTT sample one 'fresh' tumour sample placed in media<br>- 1 Fresh normal kidney sample paced in media							X	
	Within 28 days of enrolment	Day 1, Week 1	Day 1, Week 3	Day 1, Week 5	Day 1, Week 7	Day 1, Week 9	Nephrectomy	12 weeks post surgery follow up
**Blood** All translational blood (EDTA and CPDA)		X	X	X	X	X		X
**Urine** All translational urine samples		X	X	X	X	X		X

4 TISSUE ACQUISITION AND PROCESSING AT TRIAL CENTRES

4.1 TISSUE PROCESSING CONSUMABLES REQUIRED

Tissue sample kits can be requested from NAXIVA team using the order form in Appendix 1.

Provided by the NAXIVA Trial Team:

- 1.8mL sterile screw-capped cryo-tubes
- 15mL falcon tubes containing 5mL media for fresh samples (NB. These MUST be stored at 4°C immediately on receipt by sites)
- Parafilm for sealing lids of 15mL tubes containing media + fresh samples prior to storage/shipping
- Shipping boxes (2 for shipping fresh samples, plus 1 for each dry ice shipment batch)
- Ice packs for inclusion in shipping box with fresh samples
- Dry ice (this should be requested once a shipment date has been confirmed; see Section 8.3.2 for details)

- NAXIVA multiregion sampling template
- NAXIVA Sample Collection Sheets

Provided by sites:

- Liquid nitrogen for snap freezing samples (where possible). Where liquid nitrogen cannot be obtained samples may be frozen directly in -80°C freezers at site prior to transfer over dry ice.
- All reagents for processing FFPE samples (as per local protocols)

4.2 SCREENING PRIMARY TUMOUR TISSUE SAMPLE

When processing all samples for the NAXIVA trial please note the following:

- Gloves should be worn at all times when handling samples to avoid DNA contamination.
- Pipettes and consumables should be properly disposed of in accordance with institutional/hospital requirements.
- Local rules should be followed regarding hazardous waste inactivation and disposal.

Image guided percutaneous biopsy of the primary tumour should be performed prior to starting treatment (within 28 days of enrolment). Five tissue cores each from one needle puncture should be obtained. If 5 core biopsies are not obtained, follow the prioritisation scheme in section 4.2.1. Core biopsies should be processed for FFPE (2 cores), DNA/RNA (1 core), and 'fresh samples' (2 cores) as follows:

1) **FFPE samples:** Two cores should be formalin-fixed and sent for paraffin-embedding by the local site pathologist according to local protocols; one should be retained by the site and used to establish a histological diagnosis whilst the other 'research block' (labelled with the patient's study ID, biopsy date, and denoted 'Screening FFPE') should be transferred to The University of Cambridge at room temperature with other similar samples in batches after each patient has completed their 12-week post surgery visit (see Table 2).

2) **Frozen sample:** One of the remaining cores should be placed in a screw cap cryo-vial (labelled with the patient's study ID, biopsy date, and denoted 'Screening frozen core') and snap frozen in liquid nitrogen (where possible) within 30 minutes after biopsy and stored at -80°C for DNA and RNA analysis. Samples should be transferred with other samples requiring dry ice transportation to The University of Cambridge in batches after each patient has completed their 12-week post surgery visit (see Table 2).

3) **Fresh samples:** IMMEDIATELY after biopsy, the remaining 2 cores should be placed in a tube (labelled with the patient's study ID, biopsy date, and denoted 'Screening fresh core') containing 5ml media (as provided to the sites by the NAXIVA trial team; NB. These tubes must be stored at 4°C at all times from the point of receiving them), the lids tightly closed and sealed with parafilm to prevent leakage, stored upright at 4°C, and couriered to The University of Cambridge as soon as possible after biopsy (maximum within 24 hours of biopsy). These 'fresh' samples will be processed for immunophenotyping.

   Trial centres MUST e-mail Sarah Welsh (sarah.welsh@addenbrookes.nhs.uk) ,Lauren Wallis (lsgw2@cam.ac.uk), Grant Stewart (grant.stewart@addenbrookes.nhs.uk) copying in SCTRU (NSS.NAXIVA@nhs.net) at least 48 hours (preferably 1 week) BEFORE biopsies are taken so the team know to expect fresh samples - see section 8 for transfer details.

4.2.1 PRIORITISATION OF SCREENING PRIMARY TUMOUR TISSUE SAMPLES

In the event of less than 5 biopsy cores being available please prioritise samples according to the following schema:

Priority 1 - FFPE sample for diagnosis

Priority 2 – FFPE sample for 'research block'
Priority 3 – Frozen sample for DNA and RNA analysis
Priority 4 – Fresh samples for immunophenotyping (ideally 2 core biopsy samples should be placed in 5ml media for immunophenotyping, but send 1 core biopsy if 2 are not available).

4.3 NEPHRECTOMY SPECIMEN

When processing all samples for the NAXIVA trial please note the following:

- Gloves should be worn at all times when handling samples to avoid DNA contamination
- Pipettes and consumables should be properly disposed of in accordance with institutional/hospital requirements
- Local rules should be followed regarding hazardous waste inactivation and disposal

4.3.1 MULTIREGION SAMPLING

Nephrectomy specimens should be dissected by the local pathologist to obtain sufficient material for routine diagnostic purposes according to local protocols. Following this, multiregion sampling of tumour tissue (minimum of 5 areas), venous tumour thrombus and normal kidney should be undertaken with two-thirds of each sample taken for RNA/DNA analysis and one-third for immunohistochemistry (IHC; FFPE). Samples will then be placed into individual cryovials/formalin pots. Sites should aim to collect 5-10 tumour samples, 1-2 phenotypically

'normal' kidney, and 1-2 tumour thrombus samples. A map of the sampling locations must be created. See Appendix 2 for an example of tissue sampling and accompanying mapping, and Appendix 3 & 4 for a blank NAXIVA multiregion sampling template (pre-axitinib and post surgery).

4.3.2 FROZEN SAMPLES

Two-thirds of each sample from multi-region sampling (as described in Section 4.3.1) should be placed into individual 1.8mL screw cap cryovials (provided by STCRU; labelled by the site with the patient's study number, biopsy date, the corresponding multiregion sample number (e.g. Tumour 1 or Thrombus 1 etc), and denoted 'Nephrectomy frozen core') and snap frozen in liquid nitrogen (where possible) within 1 hour after the blood supply to the kidney has been interrupted and stored at -80°C at the local trial centre. Where liquid nitrogen cannot be obtained samples may be frozen and stored directly into -80°C freezers at site. Samples should be transferred in batches with other samples requiring dry ice transportation to The University of Cambridge.
During sample processing, record all details onto a NAXIVA Sample Collection Form (Appendix 3 or 4) including all the required information, any protocol deviations (including if the samples were frozen at -80°C rather than in liquid nitrogen).

4.3.3 FFPE SAMPLES

The remaining one-third of each sample taken during multiregion sampling (as described in Section 4.2.1) should be immediately formalin-fixed and paraffin-embedded according to local protocols. FFPE blocks should be stored room temperature at the local trial centre until they are transferred in batches with other samples requiring room temperature transportation to The University of Cambridge.
During sample processing, record all details onto a NAXIVA Sample Collection Form (Appendix 3 or 4) including all the required information, and any protocol deviations.

4.3.4 FRESH TUMOUR, TUMOUR VENOUS THROMBUS AND NORMAL KIDNEY SAMPLING

In addition to the multiregion sampling, approximately 1cm cubes of tumour, venous thrombus, and normal kidney should be placed in separate tubes containing 5ml media (as provided to the sites by The University of Cambridge; NB. These tubes must be stored at 4°C at all times from the point of receiving them) within 1 hour after the blood supply to the kidney has been interrupted. The lids should be tightly closed and sealed with parafilm to prevent leakage, and tubes stored upright at 4°C. These samples should be couriered to The University of Cambridge as soon as possible after collection (maximum within 24 hours of collection). These 'fresh' samples will be processed for immunophenotyping.
During sample processing, record all details onto a NAXIVA Sample Collection Form (Appendix 3 or 4) including all the required information, and any protocol deviations.
Trial centres should e-mail Sarah Welsh (sarah.welsh@addenbrookes.nhs.uk), Lauren Wallis (lsgw2@cam.ac.uk) and Grant Stewart (grant.stewart@addenbrookes.nhs.uk) copying to the SCTRU at least one week in advance of any planned surgery so the team know to expect fresh samples. These samples will be processed for immunophenotyping, and primary culture of malignant and stromal cells.
For sample shipping details please see section 8.3.

4.4 PARAFFIN EMBEDDED TISSUE SECTIONS

Paraffin embedded core biopsies that are no longer needed by the local pathologist for diagnostic purposes may be requested and transferred to the University of Cambridge as required.

5 BLOOD SAMPLING

Blood sample kits can be requested from the NAXIVA team using the order form in Appendix 5.

5.1 TIMING OF BLOOD SAMPLING

Since axitinib has a relatively short half-life **it is PREFERABLE that blood (and urine) sampling is performed immediately PRIOR to taking axitinib to allow standardisation of sampling.** Patients should ideally (where possible) be booked for blood (and urine samples) to be taken in the morning so that they can be told NOT to take axitinib until AFTER blood (and urine) samples have been collected (though patients should be advised that drinking water is allowable). Note that urine sampling also requires that patients are fasted for 6 hours before sampling (see section 6.1 for full details). However, if morning sampling is not possible then patients should be told to take their morning dose of axitinib (but should still fast for 6 hours prior to urine sampling). Both the time that the patient took their last axitinib tablet and the time that last food/ non-water drink was taken should be recorded in the sample record.

5.2 BLOOD COLLECTION SCHEDULE

Venous blood for biomarker analysis will be collected in two blood tubes (either 2 x 9ml EDTA tubes for sites processing PBMCs on-site; or 1 x 9ml EDTA and 1 x 8.5ml CPDA tube for sites NOT processing PBMCs on-site) according to local site phlebotomy procedures. **Two** tubes will be collected on day 1 week 1 prior to starting axitinib treatment, **two** tubes at the week 3 clinic visit**, two** tubes at the week 5 clinic visit, **two** tube at the week 7 clinic visit, **two** tubes at the week 9 clinic visit and **two** tubes at the

12-week clinic follow-up visit. All EDTA blood samples must be centrifuged for plasma collection **within 1 hour** to avoid fragmentation, degradation and leukocyte lysis. Subsequent isolation of PBMCs on-site should be performed within 4 hours of blood collection. No processing of CPDA tubes is done on-site and these tubes should be shipped the same-day to The University of Cambridge for processing next-day as detailed in Section 5.4.1.

The following samples must be collected at all time points from EDTA tubes:

1. **Whole blood (WB) – all centres**
2. **Plasma (P) – all centres**
3. **Buffy coat (BC) - for collection ONLY by sites NOT processing samples for PBMCs**
4. **Peripheral blood mononuclear cells (PBMCs): For collection by SELECTED SITES processing PBMCs on-site only.**

NB. All processing steps (including removal of plasma from vacutainers) should be carried out under sterile conditions e.g. HEPA filtered CL2-certified tissue culture facilities, if blood samples are being used to isolate PBMCs.

Detailed instructions for blood processing and storage of samples at local sites are contained below. Only EDTA tubes should be processed for whole blood, plasma, and buffy coat or PBMCs

- All EDTA blood samples must be centrifuged **within 1 hour** of blood collection to avoid fragmentation, degradation and leukocyte lysis.
- PBMC isolation ‘on-site’ should be performed **within 4 hours** of blood collection to maintain optimal PBMC viability.
- CPDA tubes should be shipped to The University of Cambridge on the same day as blood is taken, to arrive next day – no processing of these tubes should occur on-site. CPDA tubes should be posted pre-3pm to ensure next-day delivery to The University of Cambridge.

Blood sample kits can be requested from the NAXIVA team using the order form in Appendix 5.

5.2.1 WHOLE BLOOD (WB) – ALL CENTRES

5.2.1.1 Whole blood processing consumables required

Provided by the NAXIVA Trial Team:
- 9mL EDTA tubes
- 1.8mL sterile screw-capped cryo-tubes
- NAXIVA Blood Sample Collection Forms (Appendix 6)
- NAXIVA Blood Sample Kit Request Forms (Appendix 5)

Provided by sites:
- Standard pipettes capable of pipetting 0.5-1.0mL

5.2.1.2 Whole blood processing method

This step must be completed before plasma isolation and **within 1 hour** of blood collection.
1) During sample processing, record all required details on a NAXIVA Blood Sample Collection Form (Appendix 6), including any protocol deviations.
2) From one EDTA tube, remove 0.5ml whole blood and place into a screw cap cryovial (labelled with 'WB, patient's trial number and the date).
3) Store tubes at -80°C prior to transfer to the University of Cambridge over dry ice in batches.

5.2.2 PLASMA (P) – ALL CENTRES

5.2.2.1 Plasma processing consumables required

Provided by the NAXIVA Trial Team:
- 9mL EDTA tubes
- 1.8mL sterile screw-capped cryo-tubes
- NAXIVA Blood Sample Collection Forms (Appendix 6)
- NAXIVA Blood Kit Request Forms (Appendix 5)

Provided by sites:
- Standard pipettes capable of pipetting 0.5-1.0mL
- Microfuge capable of centrifuging microtubes at 1600g at 4ºC
- Bench top centrifuge capable of centrifuging 9mL EDTA tubes at 400g

5.2.2.2 Plasma sample processing method

Important: Blood samples should be centrifuged **as soon as possible** after collection, **within 1 hour** to avoid fragmentation, degradation and leukocyte lysis.
1) During sample processing, record all required details on a NAXIVA Blood Sample Collection Form including any protocol deviations.
2) Following transfer of whole blood (above) from one EDTA blood tube, spin one EDTA tube (sites NOT processing PBMCs on site) or both EDTA tubes (sites processing PBMCs on-site) at 400g in a bench-top centrifuge for 15 minutes at room temperature.
3) Transfer the plasma into multiple microfuge-tubes in equal aliquots
4) Centrifuge the plasma aliquots in a microfuge at 1600g for 10 min at 4° C.
5) Transfer the re-centrifuged plasma into labelled 1.8mL screw-cap cryovials (labelled P/patient's trial ID/date). Take care not to disturb the pelleted cells at the bottom of each tube. Aim to collect 4 cryovials with roughly even amounts of plasma in each.
6) Store the EDTA blood tube with remaining blood at 4° C for collection of buffy coat or PBMCs.
7) Store plasma at -80°C at the trial centre until transfer to University of Cambridge on dry ice in batches.

5.2.3 BUFFY COAT (BC) - FOR COLLECTION ONLY BY SITES NOT PROCESSING SAMPLES FOR PBMCS (AS THIS STEP REMOVES THE PERIPHERAL BLOOD MONONUCLEAR CELLS; PBMCS)

5.2.3.1 Buffy coat processing consumables required

Provided by NAXIVA Trial Team:
- 9mL EDTA tubes
- 1.8mL sterile screw-capped cryo-tubes
- NAXIVA Blood Sample Collection Forms (Appendix 6)
- NAXIVA Blood Sample Kit Request Forms (Appendix 5)

Provided by sites:
- Standard pipette capable of pipetting 0.5mL
- Microfuge capable of centrifuging microtubes at 1600g

5.2.3.2 Buffy coat collection protocol

1) During sample processing, record all required details onto a NAXIVA Blood Sample Collection Form, including any protocol deviations.
2) Following removal of whole blood and plasma from the EDTA blood tubes, remove the buffy coat with about 100ul of plasma taking care not to lift the red cells. The buffy coat layer lies just above the red cells and contains the white blood cells.
3) Label the cryo-tubes with BC/patient's trial ID/date.
4) Store at -80°C at the trial centre until transfer to University of Cambridge on dry ice in batches.

5.2.4 PERIPHERAL BLOOD MONONUCLEAR CELLS (PBMCS): FOR COLLECTION BY SELECTED SITES ONLY.

<u>Do NOT</u> collect buffy coat if processing blood for PBMCs as buffy coat contains PBMCs resulting in very low yield using this protocol.

5.2.4.1 PBMC processing consumables required

Provided by the NAXIVA Trial Team:
- SepMate tube
- Ficoll
- Freezing mix (must be stored at 4ºC or -20 ºC)
- Phosphate buffered saline (must be stored at 4ºC)
- 50mL Falcon tubes
- 1.8mL sterile screw-capped cryo-tubes
- NAXIVA Blood Sample Collection Forms (Appendix 6)
- NAXIVA Blood Kit Request Forms (Appendix 5)

Provided by sites:
- HEPA filtered sterile tissue culture cabinet in CL2 facilities
- Transfer (Fisher Scientific: 2655116) or standard pipette capable of pipetting 4-50mL
- Bench top centrifuge capable of centrifuging 50mL Falcon tubes at 1200g
- Container capable of freezing cells in cryo-tubes slowly (Mr Frosty/similar container)

5.2.4.2 PBMC sample processing method

All processing steps (including removal of plasma from EDTA blood tubes) should be carried out under sterile conditions e.g. HEPA filtered CL2-certified tissue culture facilities, if blood samples are being used to isolate PBMCs.

1) During sample processing, record all details on a NAXIVA Sample Collection Form (Appendix 6) including any protocol deviations.
2) Ideally this step must be completed within 4 hours of blood sampling, but certainly completed same day. If unable to process samples immediately after plasma extraction, store samples at 4°C.

3) Remaining blood from Section 5.3.2.2 (step 6) is used to isolate PBMCs in the following steps.
4) After removing plasma dilute the remaining blood in the tubes 1:1 with pre-warmed (room temperature) sterile phosphate buffered saline (if a full 9ml EDTA tube was taken from the patient the volume of PBS to add will be approximately 4ml).
5) Mix tube well by inverting 3 times.
6) Keeping the SepMate tube upright, pipette 15ml Ficoll through the central hole of the insert in the SepMate tube. The top of the Ficoll will come to ABOVE the level of the insert. Air bubbles may be seen under the insert and are reduced by pipetting the Ficoll more slowly through the insert, but these don't affect the cell separation.
7) Pipette the blood/PBS mix down the side of the SepMate tube (pool blood/PBS from both EDTA tubes into one SepMate tube).
8) Centrifuge tubes at 1200g for 10 minutes at room temperature (using normal brake settings i.e. brake on).
9) Remove tubes from centrifuge and collect the mononuclear cells by inverting the SepMate tube into a 50ml Falcon tube in one smooth movement. Do not hold the SepMate tube inverted for >2 seconds or the red cells (which may be seen on the surface of the SepMate insert before inversion in addition to the bottom of the tube) start to dribble through the insert and will contaminate the mononuclear cells.
10) Dilute mononuclear cells to 45ml with pre-warmed PBS.
11) Centrifuge tubes at 1200g for 10 minutes at room temperature (normal brake settings) to wash the cells.
12) Discard supernatant (carefully as cell pellet may be slimy).
13) Re-suspend cell pellet in 4ml 10% DMSO in fetal calf serum ('freezing mix') and freeze 4 x 1mL aliquots in labelled (PBMC/patient's trial number/date) cryovials of peripheral blood mononuclear cells (aliquot approximately equal volumes into each cryovial).
14) Immediately place at -80°C (in a pre-cooled (4°C) Mr Frosty/ similar container). Store at -80°C for up to a week, and then ideally transfer to liquid nitrogen until transfer on dry ice to University of Cambridge.

5.3 BLOOD SAMPLE TRANSFER TO THE UNIVERSITY OF CAMBRIDGE

5.3.1 CPDA BLOOD TUBE TRANSFER TO THE UNIVERSITY OF CAMBRIDGE

Immediately after sampling, CPDA blood tubes should be inverted gently and stored at room temperature until transfer to The University of Cambridge. Where possible, CPDA blood tubes should not be posted on a Friday. For shipping, the CPDA blood tubes should be placed inside the transfer canisters (provided by the NAXIVA trial team) and the lid screwed on securely. The container and a copy of the NAXIVA Blood Sample Form (Appendix 6) should then be placed into the cardboard shipping boxes (provided by the NAXIVA trial team) and placed inside a jiffy bag (pre-paid and labelled by the NAXIVA team and sent via Royal Mail ON THE DAY OF SAMPLING to The University of Cambridge.

Trial centres should e-mail Sarah Welsh (sarah.welsh@addenbrookes.nhs.uk), Lauren Wallis (lsgw2@cam.ac.uk) and Grant Stewart (grant.stewart@addenbrookes.nhs.uk) copying to the SCTRU at least one week in advance of any planned CPDA blood tube transfer so the team know to expect blood samples.

5.3.2 WHOLE BLOOD, PLASMA, BUFFY COAT AND PBMCS – TRANSFER TO THE UNIVERSITY OF CAMBRIDGE

After processing all EDTA blood samples from all time points for one patient, samples should be transferred in a batch on dry ice with other samples requiring dry ice transportation to The University of Cambridge (see section 8.3.2. for details). A copy of the blood sample collection form (Appendix 6) for every time point should be included when blood samples are transferred to The University of Cambridge.

6 URINE SAMPLES

6.1 TIMING OF URINE SAMPLING

It is ESSENTIAL for successful downstream analysis that urine sampling is performed when the patient has fasted for 6 hours. Patients should ideally (where possible) be booked for blood and urine samples to be taken in the morning so that they can be told NOT to take axitinib until AFTER their urine and blood samples have been collected (though patients should be advised that drinking water is allowable). However, if morning sampling is not possible then patients should be told to take their morning dose of axitinib (but should still fast for 6 hours prior to urine sampling). Both the time that the patient took their last axitinib tablet and the time that last food/ non-water drink was taken should be recorded in the NAXIVA Urine Sample Collection form.

6.2 URINE COLLECTION SCHEDULE

Urine samples will be collected on day 1 week 1, day 1 week 3, day 1 week 5, day 1 week 7, day 1 week 9, and at the 12-week follow-up clinic visit.

6.3 URINE SAMPLE PROCESSING PROTOCOLS

Collect at least 23mL of freshly voided midstream urine from each patient at each time point into a sterile container. If required, pool urine from different containers together.
The urine sample will be processed in two ways as follows:
1. A 20mL aliquot of urine will be treated with EDTA immediately to avoid nuclease degradation, as detailed in Section 6.3.1. This will be used for measurement of ctDNA.
2. A further 3mL aliquot of urine will also be stored and will not be treated with EDTA, as detailed in Section 6.3.1. This will be used for metabolomics and cytokine analyses.

6.3.1 PROCESSING OF URINE SAMPLES FOR CTDNA ANALYSIS

NOTE: If <23mL of urine is collected, then remove 3mL of untreated urine prior to conducting EDTA treatment. Then adjust the amount of EDTA added to the treated urine specimen to gain a final concentration of 10mM EDTA.

6.3.1.1 Consumables required

Urine sample kits can be requested from the NAXIVA team using the order form in Appendix 7.

Provided by the NAXIVA Trial Team:

- 30mL sterilin tubes for urine collection
- 50mL Falcon tubes
- 15mL Falcon tubes
- Ultrapure 0.5M EDTA, pH8.0
- 1.8mL screw-capped cryo-tubes
- NAXIVA Urine Sample Collection Forms (Appendix 8)
- NAXIVA Urine Kit Request Forms (Appendix 7)

Provided by sites:

- Pipettes and tips (capable of pipetting 400µL)
- Bench top centrifuge capable of centrifuging 50mL Falcon tubes at 3000g

6.3.1.2 Method for EDTA-treated urine sample processing (for ctDNA analysis)

IMPORTANT: To inhibit possible nuclease degradation, the sample should be mixed with EDTA and centrifuged **as soon as possible** after collection but certainly within 1 hour of sampling.

1. During sample processing, record all details required on the NAXIVA Urine Sample Collection Form (Appendix 8), including any protocol deviations.
2. Transfer 20mL of urine into a labelled sterile 50mL Falcon tube.
3. Add 400uL of 0.5M EDTA (pH8.0) to 20mL of urine
4. Gently invert tube 8-10 times to obtain a final concentration of 10mM EDTA
5. Centrifuge samples at 3000g for 10 minutes. Urine samples should be centrifuged within 1 hour after collection to remove cellular debris.
6. Transfer 10mL aliquots of supernatant into 2 sterile 15mL Falcon tubes.
7. Label each falcon tube with the site code, patient trial ID, date and time point of sampling (e.g. Screening, Day 1 Week 1 etc).
8. Freeze aliquots upright in a -80°C freezer.
9. After 24 hours, samples can be stored flat if required (at -80°C) until transfer on dry ice to University of Cambridge in batches.

6.3.1.3 Method for untreated urine sample processing (for metabolomics and cytokine analyses

1. During sample processing, record all details on a NAXIVA Urine Sample Collection Form.
2. Take 3 x 1 mL aliquots from the freshly voided urine (no EDTA added) and place each 1mL aliquot into a 1.8mL screw cap cryo-tube.
3. Label each cryo-tube with the site code, patient trial ID, date, and time point of sampling (e.g. Screening, Day 1 Week 1 etc).
4. Transfer cryo-tubes to a -80°C freezer and store until transfer in batches on dry ice to The University of Cambridge.

6.4 URINE SAMPLE TRANSFER TO UNIVERSITY OF CAMBRIDGE

Urine samples should be transferred on dry ice in batches with other samples requiring dry ice transportation to The University of Cambridge. See Section 8 for details. Copies of the urine sample collection forms (Appendix 8) should be included in the package for each urine sample transferred to The University of Cambridge.

7 STORAGE OF SAMPLES

Storage conditions for all samples for NAXIVA translational studies are detailed in the individual sections above.

8 TRANSFER OF SAMPLES TO THE UNIVERSITY OF CAMBRIDGE

8.1 GENERAL INSTRUCTIONS

Trial centres MUST notify SCTRU at least 1 week in advance of transferring samples and notify SCTRU of any tracking details for the samples once sent.

When booking the courier trial centres must ensure they provide the address and phone number of Lauren Wallis (recipient of the samples).

- Storage/transfer boxes should be requested from the NAXIVA team upon reaching the last box to ensure sufficient time for postage.
- Non time-urgent samples should be shipped early in the week (Monday to Wednesday morning) to avoid arrival at the University of Cambridge over the weekend.

- All samples (except FFPE blocks) should be shipped using the courier details provided below.
- Samples should be transported under the following transfer conditions:
  - FFPE blocks should be transferred at room temperature in batches containing all the FFPE samples for a single patient, preferably in a padded ‘jiffy bag’
  - ‘Fresh’ core biopsies, ‘fresh’ tumour/ tumour thrombus/ normal kidney tissue for immunophenotyping and cell line generation should be transferred WITHIN 24 hours of collection, at 4ºC (i.e. in an insulated box with ice packs to maintain 4ºC during transfer – these will be provided by the NAXIVA Trial Team).
  - Fresh frozen core biopsies, fresh frozen tumour/ tumour thrombus/ normal kidney tissue, tumour tissue from multiregion sampling, whole blood, plasma, buffy coat or PBMCs, and urine should be transferred in batches containing all the samples for a single patient after all sampling visits for that patient have been completed. Samples should be sent on dry ice using the courier service detailed below, in an insulated box as provided by the NAXIVA Trial Team.

Sites must ensure that all samples transferred to the University of Cambridge are done so in accordance with the UN3373 biological substance regulation.

8.2 DOCUMENTS REQUIRED

8.2.1 ‘FRESH’ SAMPLES

1) As early as possible (but at least a week prior to biopsy or surgery) e-mail the NAXIVA team at SCTRU to propose a shipment date.
2) The NAXIVA Sample Collection Form (Appendix 3 or 4) with all details completed for samples being shipped should be copied and included in the package containing the samples.

8.2.2 ALL OTHER SAMPLES EXCEPT ‘FRESH’ SAMPLES

1) One week prior to the expected shipment date sites should email the NAXIVA team at SCTRU to inform the translational team of the expected shipment date.
   The completed Blood/ Urine/ Tissue NAXIVA Sample Collection Forms (Appendix 3, 4, 6 & 8) for each sample being shipped should be copied and included in the package containing the samples.

8.3 SHIPMENT PROCESS

Table 2- Sample Shipment Schedule

Shipment Type	Applicable Samples	When are the samples collected	Shipment Method
Samples must be shipped 'overnight' within 24 hours of collection	Fresh Tissue Samples	**Screening** (Image guided biopsy)<br>**Week 9** (nephrectomy)	Eagle Couriers 'OVERNIGHT pre-12:30' (see section 8.3.1)
The NAXIVA team will request samples to be sent in bulk **after the patient has completed trial**	1. Frozen Tissue Samples	**Screening** (Image guided biopsy)<br>**Week 9** (nephrectomy)	Eagle Couriers 'OVERNIGHT' (see section 8.3.2)
	2. Frozen Blood Samples (all EDTA tubes)	**Week 1,3,5,7,9** and **12 weeks** post surgery follow up	
	3. Frozen Urine Samples	**Week 1,3,5,7,9** and **12 weeks** post surgery follow up	
	4. FFPE Tissue Samples	**Screening** (Image guided biopsy)<br>**Week 9** (nephrectomy)	Eagle Couriers 'OVERNIGHT' (see section 8.3.3)
Only where sites aren't processing PBMC at site<br>Samples must be shipped on the same day of collection	1. PBMC Blood Samples (CPDA tubes)	**Week 1,3,5,7,9** and **12 weeks** post surgery follow up	Royal Mail, pre-paid special delivery (see section 8.3.4)

8.3.1 'FRESH' SAMPLES FOR TRANSFER WITHIN 24 HOURS OF COLLECTION

1) As early as possible (but at least a week prior to biopsy or surgery) e-mail the NAXIVA team at SCTRU to propose a shipment date. Plan to ship samples the same day as they are collected such that they are couriered overnight and reach The University of Cambridge in the morning to allow maximum processing time. Samples should be shipped within 24 hours of sampling at the latest.
2) Await e-mail confirmation to ensure that The University of Cambridge will be available to receive the samples
3) Prior to organising shipment check you have the appropriate shipping box and ice packs in stock. Place ice packs in the freezer to cool at least 24 hours in advance of shipment.
4) Within the email confirmation you will be provided with a PO number which should be quoted when contacting the courier company.
5) Contact "Eagle Couriers" on Tel: 08451231230 and provide the following details:
    - o Quote account number 'NHSN1091'
    - o State that the shipment is for the "NAXIVA" study
    - o Quote the PO number (given in the confirmation email from SCTRU) to Request **OVERNIGHT** (pre-12:30) delivery for the shipment
    - o Please ensure you provide telephone details for the **Lauren Wallis** to the courier (01223 348441). The telephone details should also be added to the address label (as below).
    - o The delivery address details are:

Lauren Wallis (Urological Malignancies Sample Handler)
Oncology Outpatient Reception
Addenbrooke's Hospital
Hills Road
Cambridge
CB2 0QQ
Tel: 01223 348441

6) Before packing, the tubes containing media and sample should be tightly closed and parafilm should be used to seal around the lids to prevent leakage. The tubes should be placed inside a plastic bag containing absorbent material in case of spillage then into the polystyrene box provided by the NAXIVA team with an ice pack, such that the samples are likely to remain cold (but not frozen) for up to 24 hours (in case of delays).
7) Include a copy of the NAXIVA Sample Collection Form (either Appendix 3 or 4) inside the package.
8) Place a biological hazard logo (Appendix 9) on the box, plus the address above.
9) On sending the package email the NAXIVA team to inform the team that samples are in transit.
10) Advise the NAXIVA team of tracking details for the package.
11) Once samples have arrived at The University of Cambridge, a confirmation of receipt email will be issued.

8.3.2 FROZEN SAMPLES FOR TRANSFER ON DRY ICE

1) All frozen samples from a single patient (e.g. whole blood, plasma, buffy coat or PBMCs, urine, and frozen tissue) should be transferred as a batch after ALL samples have been processed for that patient. Ensure that the relevant NAXIVA Sample Collection Forms have been completed and copies are included in the shipment with the samples.
2) As early as possible (but at least a week prior to expected transfer of samples) e-mail the NAXIVA team at SCTRU to propose a shipment date.
3) Await e-mail confirmation to ensure that The University of Cambridge will be available to receive the samples.
4) Prior to organising shipment check you have the appropriate shipping box in stock.
5) Within the email confirmation you will be provided with a PO number which should be quoted when contacting the courier company (see point 7 below).
6) To order the appropriate amount of dry ice required for transfer of frozen samples to The University of Cambridge, please email: Alison Warrington (acw39@cam.ac.uk) or Lila Tran (nmtt2@medschl.cam.ac.uk). In the email please specify the following details:

    1. How much dry ice is required (we suggest 10kg per box may be appropriate to order)
    2. Contact Details
    3. Delivery Address
    4. Time Frame required
    5. Alison/Lila will order the dry ice directly from ‘Dry Ice UK’ and cc you into all correspondence
    6. Please file all orders and emails within the Investigator Site File.

7) Contact “Eagle Couriers" Courier Services on Tel: 08451231230 and provide the following details:
    - o Quote account number ‘NHSN1091’
    - o State that the shipment is for the “NAXIVA” study
    - o Quote the PO number (given in the confirmation email from SCTRU)
    - o Request **OVERNIGHT** delivery for the shipment
    - o Please ensure you provide telephone details for the **Lauren Wallis** to the courier (01223 348441). The telephone details should also be added to the address label (as below).
    - o The delivery address details are:

Lauren Wallis(Urological Malignancies Sample Handler)
Oncology Outpatient Reception
Addenbrooke’s Hospital
Hills Road
Cambridge
CB2 0QQ
Tel: 01223 348441

8) When packing samples for transfer, samples should be placed inside waterproof bags containing similar types of samples e.g. all PBMCs in one bag, all plasma samples in another bag etc, to aid sample logging on arrival at The University of Cambridge. Include an absorbent material in each bag in case of spillages. Include copies of the relevant NAXIVA Blood, Urine and Tissue Sample Collection Forms (Appendix 3, 4, 6 & 8).
9) Add sufficient dry ice to the box to last 48 hours (in case of delays).
10) Place a biological hazard logo (Appendix 9) on the box, plus the address above
11) Advise the NAXIVA team at SCTRU of tracking details for the package.
12) Once samples have arrived at The University of Cambridge, a confirmation of receipt email will be issued.

8.3.3 FFPE BLOCKS FOR TRANSFER AT ROOM TEMPERATURE

1) All FFPE research blocks from a single patient i.e. both the pre-treatment core biopsies and the FFPE blocks collected at nephrectomy, should be transferred as a batch after ALL samples have been processed for that patient. Ensure that the relevant NAXIVA Sample Collection Forms have been completed (Appendix 3&4) and are included in the shipment with the samples.
2) As early as possible (but at least a week prior to transferring samples) e-mail the NAXIVA team at SCTRU to propose a shipment date.
3) Await e-mail confirmation to ensure that The University of Cambridge will be available to receive the samples.
4) FFPE blocks can be transferred at room temperature according to local protocols. It is suggested that these are transferred in padded 'jiffy bags' using 'tracked and signed for' mail services. No other samples are transferred at room temperature. Advise the NAXIVA team at SCTRU of tracking details for the package.
5) Contact "Eagle Couriers" Courier Services on Tel: 08451231230 and provide the following details:
   - o Quote account number 'NHSN1091'
   - o State that the shipment is for the "NAXIVA" study
   - o Quote the PO number (given in the confirmation email from SCTRU)
   - o Request **OVERNIGHT** delivery for the shipment
   - o Please ensure you provide telephone details for the **Lauren Wallis** to the courier (01223 348441). The telephone details should also be added to the address label (as below).
6) The delivery address details are:

Lauren Wallis (Urological Malignancies Sample Handler)
Oncology Outpatient Reception
Addenbrooke's Hospital
Hills Road
Cambridge
CB2 0QQ
Tel: 01223 348441

7) Once samples have arrived at The University of Cambridge, a confirmation of receipt email will be issued.

8.3.4 CPDA TUBES FOR TRANSFER AT ROOM TEMPERATURE (FOR PBMC PROCESSING AT UNIVERSITY OF CAMBRIDGE)

1) As early as possible (but at least a week prior to expected transfer of samples) e-mail the NAXIVA team at SCTRU to inform them of a shipment date.
2) CPDA tubes containing whole blood should be shipped on the same day as blood is taken from the patient. CPDA tubes should be posted pre-3pm to ensure next-day delivery to The University of Cambridge,
3) CPDA tubes should be placed inside the transfer canisters (provided by the NAXIVA trial team) and the canister lid screwed on securely. The canister and a copy of the NAXIVA Blood Sample Form (Appendix 6) should then be placed into the cardboard shipping boxes (provided by the NAXIVA trial team) and placed inside a jiffy bag (pre-paid, addressed and labelled by the NAXIVA trial team) and sent via Royal Mail ON THE DAY OF SAMPLING to The University of Cambridge.

4) Once samples have arrived at The University of Cambridge, a confirmation of receipt email will be issued.
5) Please ensure you provide telephone details for the **Lauren Wallis** to the courier (01223 348441). The telephone details should also be added to the address label (as below).
6) The delivery address details are:

Lauren Wallis (Urological Malignancies Sample Handler)
Oncology Outpatient Reception
Addenbrooke's Hospital
Hills Road
Cambridge
CB2 0QQ
Tel: 01223 348441

7) Assembly instructions for shipping boxes:

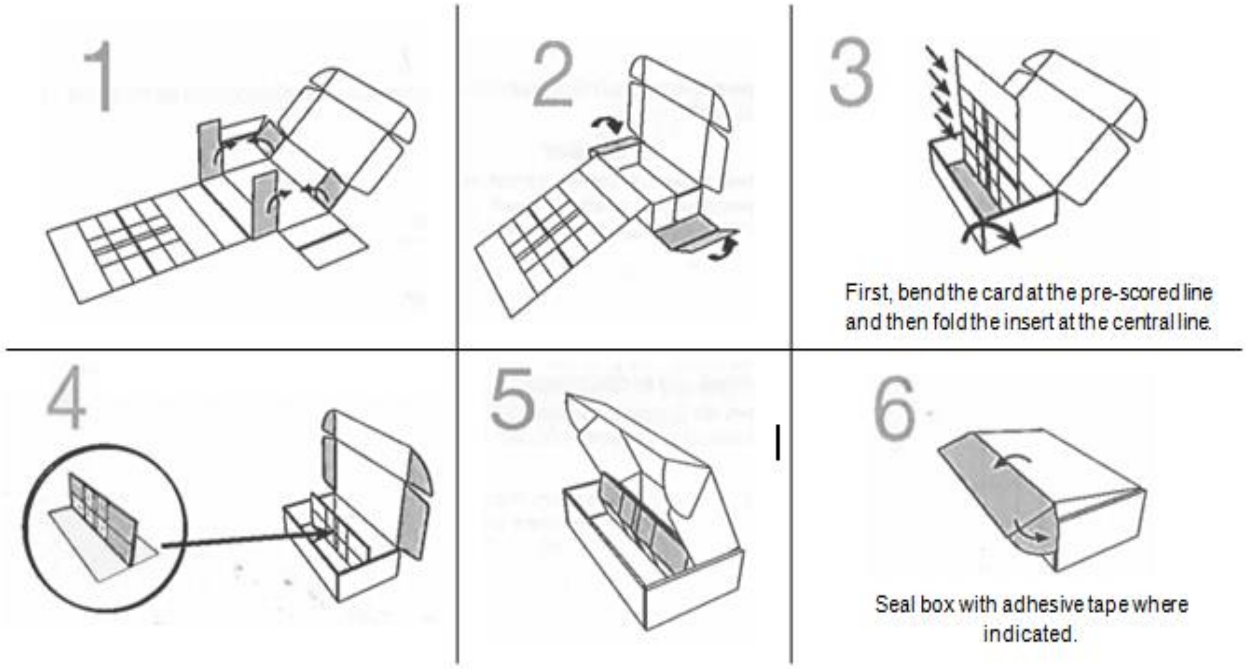

APPENDIX INDEX

Appendix 1- Tissue Sample Kits Request Form

Appendix 2- Tissue Sampling and Accompanying Mapping Example

Appendix 3- Kidney Pre-Axitinib Sampling Form

Appendix 4- Nephrectomy Sampling Form

Appendix 5- Blood Sample Kit Request Form

Appendix 6- Blood Sample Collection Form

Appendix 7- Urine Sample Kit Request Form

Appendix 8- Urine Sample Collection Form

Appendix 9- Biological hazard shipping label

APPENDIX 1

NAXIVA TRANSLATIONAL STUDY

TISSUE SAMPLE KIT REQUEST FORM

TO ORDER 2 KITS (1 patient pack)

When a new patient is registered you will receive 2 tissue sample kits (one for kidney screening samples and one for nephrectomy samples). If you do not receive these, please **copy** this form, **complete** and **email** to:-

FAO: NAXIVA Trial Team

Email: NSS.NAXIVA@nhs.net

(PLEASE PRINT DETAILS)

Contact name ________________________________ Date ______________

Email address ____

Hospital ____

Address ____

____

____

____

For office use: - Tissue Kits despatched

Comments:

If you experience any difficulties or have any questions please email the NAXIVA Trial Team; NSS.NAXIVA@nhs.net

APPENDIX 2

NAXIVA TRANSLATIONAL STUDY

<u>EXAMPLE OF MULTIREGION SAMPLING AND DOCUMENTAITON</u>

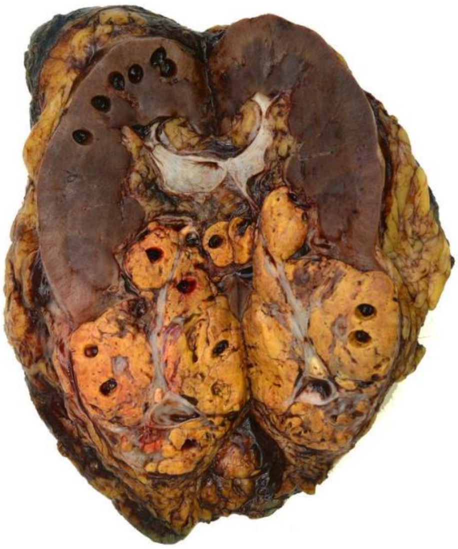

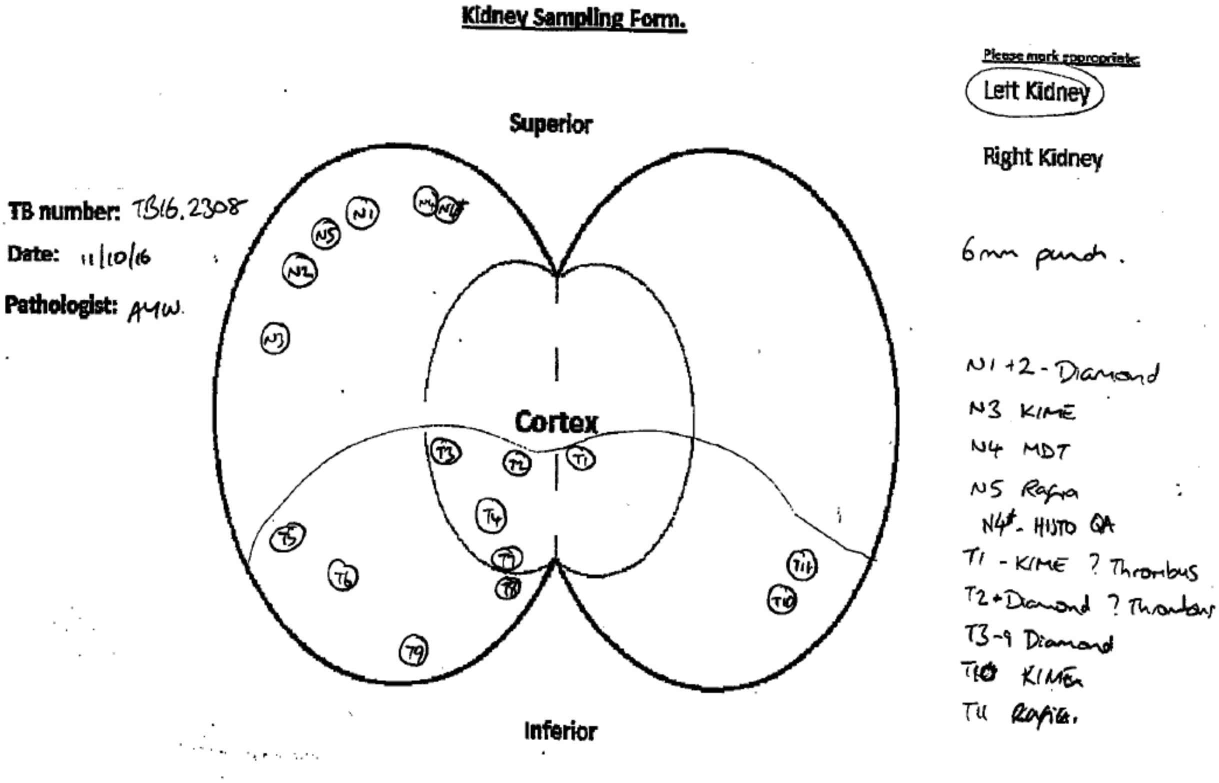

AFFIX DATA MATRIX LABEL

FOR LAB USE ONLY

APPENDIX 3

NAXIVA TRANSLATIONAL STUDY

KIDNEY PRE-AXITINIB SAMPLING FORM

Patient initials: | | |

Date of birth: | | | | | | | |

Trial ID: | | | | |

Centre/Hospital:

Informed consent checked (Translational Study) by;

Name:

Date: D D M M Y Y Y Y

Kidney Tumour Biopsy

Tumour Biopsy Date: D D M M Y Y Y Y

Tumour Biopsy Number: ___________________________________

Pathologist:____

Tumour Side *(please tick)*: Right Kidney ☐ Left Kidney ☐

Please annote where tumour located and where the research tissue samples taken from in tumour and normal kidney;

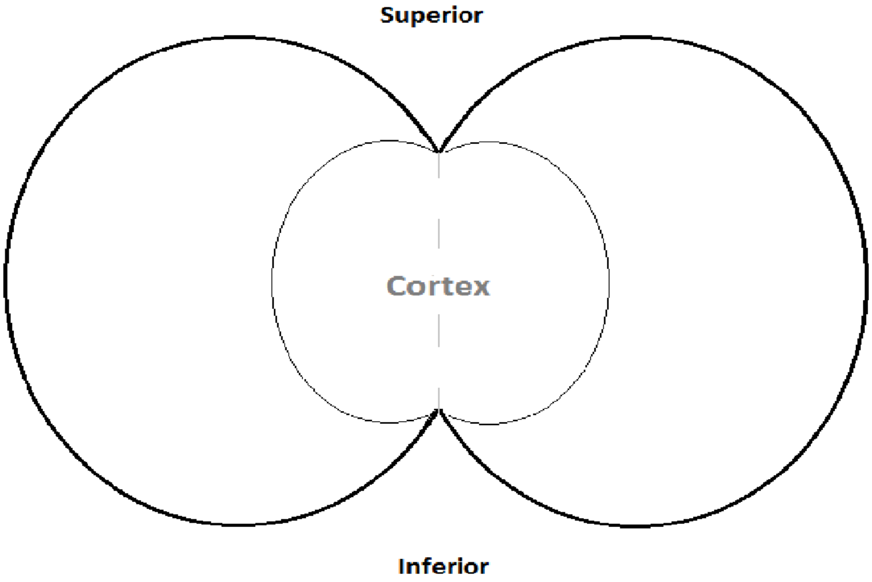

Completion Details

Date form completed: D D M M Y Y Y Y

Signature: _____________________________

Print Name: ____________________________

APPENDIX 4

NAXIVA TRANSLATIONAL STUDY

NEPHRECTOMY SAMPLING FORM- page 1

AFFIX DATA MATRIX LABEL

FOR LAB USE ONLY

Patient initials: | | | |

Date of birth: | | | | | | | | |

Trial ID: | | | | | |

Centre/Hospital: ____

Informed consent checked (Translational Study) by;

Name: ____ Date: | D | D | M | M | Y | Y | Y | Y |

<u>Nephrectomy Samples</u>

Nephrectomy Date: | D | D | M | M | Y | Y | Y | Y |

Nephrectomy Sample Number: ___________________________________

Pathologist:____

Tumour Side *(please tick)*: Right Kidney ☐ Left Kidney ☐

Please annote where tumour located and where the research tissue samples taken from in tumour and normal kidney;

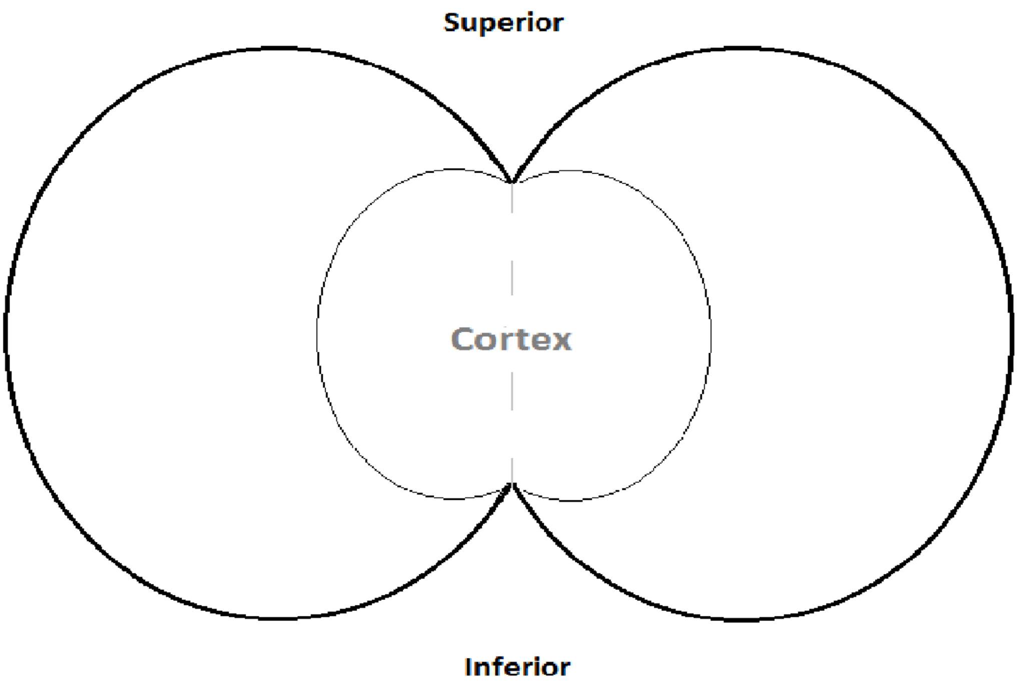

NAXIVA TRANSLATIONAL STUDY

NEPHRECTOMY SAMPLING FORM- page 2

Patient initials: ☐☐☐ Date of birth: ☐☐☐☐☐☐☐☐

Trial ID: ☐☐☐☐☐ Centre/Hospital: ☐

Tumour Thrombus Samples

Appearance of thrombus, *please tick*

Solid ☐ Friable/necrotic ☐ Mixed ☐

Location of thrombus tissue sampling (please sample solid, non necrotic tissue if present):

Leading edge ☐

Middle of thrombus ☐

Unclear due to state of thrombus ☐

Additional Comments: ____

____

____

____

____

Completion Details

Date form completed: D D M M Y Y Y Y Signature: ____________________

Print Name: ____________________

APPENDIX 5

NAXIVA TRANSLATIONAL STUDY

BLOOD SAMPLE KIT REQUEST FORM
(1 full patient pack)

When a new patient is registered you will receive blood sample kits. If you do not receive these, please **copy** this form, **complete** and **email** to:-

FAO: NAXIVA Trial Team

Email: NSS.NAXIVA@nhs.net

(PLEASE PRINT DETAILS)

Contact name ______________________________ Date ______________

Email address ____

Hospital ____

Address ____

____

____

____

For office use: - Blood Kits despatched

Comments:

If you experience any difficulties or have any questions please email the NAXIVA Trial Team; NSS.NAXIVA@nhs.net

AFFIX DATA MATRIX LABEL

FOR LAB USE ONLY

APPENDIX 6

NAXIVA TRANSLATIONAL STUDY

BLOOD SAMPLE COLLECTION FORM

Patient initials: | | |

Date of birth: | | | | | | | |

Trial ID: | | | | |

Centre/Hospital:

Informed consent checked (Translational Study) by;

Name:

Date: D D M M Y Y Y Y

Blood Sample(s) Information

Blood sample (s) have been taken from visits; *(please tick)*	
Week 1 (Screening)	
Week 3	
Week 5	
Week 7	
Week 9	
12 Week post surgery	

→

Type of blood sample taken; *(please tick)*	
Whole Blood	
Plasma	
Buffy Coat	
PBMCs	

Date sample taken: D D M M Y Y Y Y

Time last axitinib taken	*HH:MM*		
Time Sample Taken	*HH:MM*		
Time Centrifuged *(Within 1 hour)*	*HH:MM*		
Time PBMCs Isolated *(Within 4 hours)*	*HH:MM*	N/A	
Time Sample Frozen	*HH:MM*		

Comments: ____

____

Completion Details

Date form completed: D D M M Y Y Y Y

Signature: ______________________

Print Name: ______________________

APPENDIX 7

NAXIVA TRANSLATIONAL STUDY

URINE SAMPLE KIT REQUEST FORM

(1 patient pack)

When a new patient is registered you will receive urine sample kits. If you do not receive these, please **copy** this form, **complete** and **email** to:-

FAO: NAXIVA Trial Team

Email: NSS.NAXIVA@nhs.net

(PLEASE PRINT DETAILS)

Contact name ______________________________ Date ______________

Email address ____

Hospital ____

Address ____

____

____

____

For office use: - Urine Kits despatched

Comments:

If you experience any difficulties or have any questions please email the NAXIVA Trial Team; NSS.NAXIVA@nhs.net

AFFIX DATA MATRIX LABEL

FOR LAB USE ONLY

APPENDIX 8

NAXIVA TRANSLATIONAL STUDY

URINE SAMPLE COLLECTION FORM

Patient initials: [ ][ ][ ] Date of birth: [ ][ ][ ][ ][ ][ ][ ][ ] Trial

ID: [ ][ ][ ][ ][ ] Centre/Hospital: [ ]

Informed consent checked (Translational Study) by;

Name: [ ]

Date: D D M M Y Y Y Y

<u>Urine Sample(s) Information</u>

Urine sample(s) included have been taken from visit(s);

	Week 1	*Week 3*	*Week 5*	*Week 7*	*Week 9*	*12 weeks post surgery*
Date sample taken	*DD/MM/YYYY*	*DD/MM/YYYY*	*DD/MM/YYYY*	*DD/MM/YYYY*	*DD/MM/YYYY*	*DD/MM/YYYY*
Time last axitinib taken	*HH:MM*	*HH:MM*	*HH:MM*	*HH:MM*	*HH:MM*	*HH:MM*
Time food (or non-clear fluids) last taken	*HH:MM*	*HH:MM*	*HH:MM*	*HH:MM*	*HH:MM*	*HH:MM*
Time sample taken	*HH:MM*	*HH:MM*	*HH:MM*	*HH:MM*	*HH:MM*	*HH:MM*
Time Centrifuged	*HH:MM*	*HH:MM*	*HH:MM*	*HH:MM*	*HH:MM*	*HH:MM*
Time sample frozen	*HH:MM*	*HH:MM*	*HH:MM*	*HH:MM*	*HH:MM*	*HH:MM*

SAMPLES NEED TO BE FROZEN AS SOON AS POSSIBLE AFTER PROCESSING.

Comments: ____

____

____

<u>Completion Details</u>

Date form completed: D D M M Y Y Y Y Signature: ____________________

Print Name: ____________________

APPENDIX 9

NAXIVA TRANSLATIONAL STUDY

BIOLOGICAL LABEL FOR ATTACHING TO SHIPMENTS OF SAMPLES

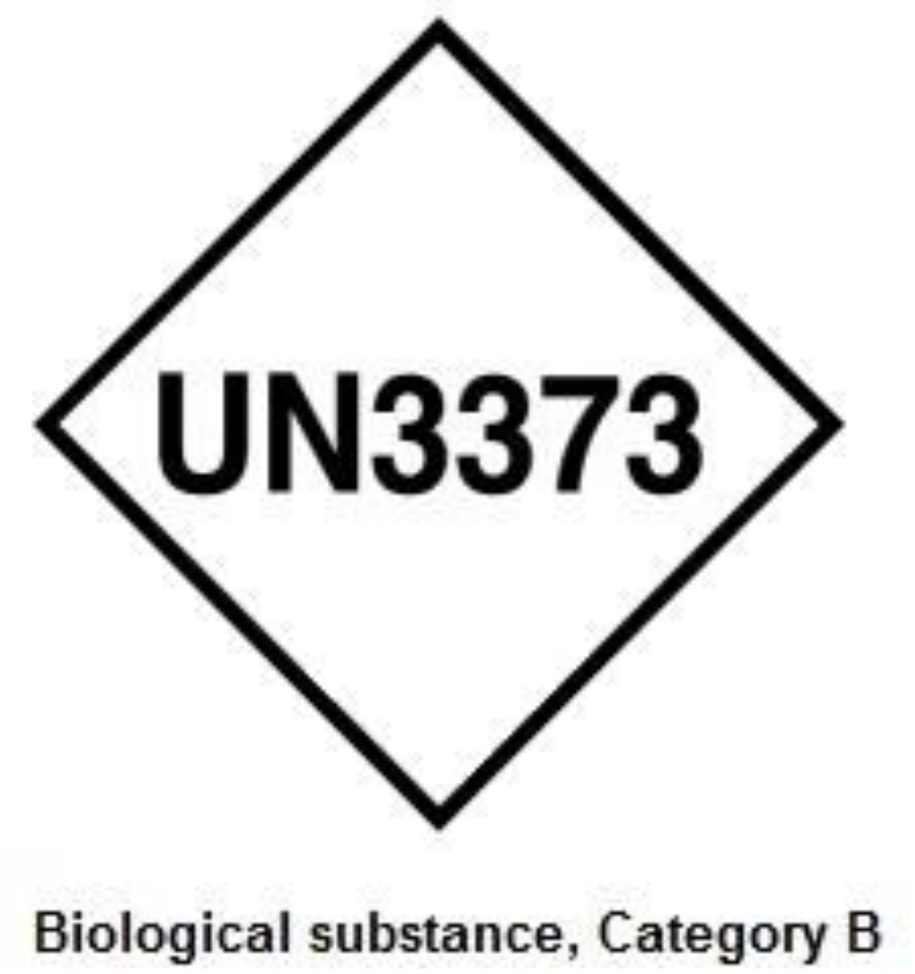

Supplement: Supplementary file 4 — Sampling handling manual [file 41416_2022_1883_MOESM4_ESM.pdf]
